# GTPase GBP7 promotes HSV-1 infection by interacting with and activating eEF1A1 to accelerate the protein translation process

**DOI:** 10.1371/journal.ppat.1014652

**Published:** 2026-10-12

**Authors:** Jinshen Wang, Liangbing Man, Binhao Rong, Kunyu Lu, Luping Cheng, Jianfu He, Haowei Li, Hao Su, Xinxin Lin, Ru Li, Min Zou, Chan Yang, Shuwen Liu

**Affiliations:** 1 Guangdong Provincial Key Laboratory of New Drug Screening, Guangdong-Hong kong-Macao Joint Laboratory for New Drug Screening, School of Pharmaceutical Sciences, Southern Medical University, Guangzhou, China; 2 Department of Pharmacy, Third Affiliated Hospital of Sun Yat-sen University, Guangzhou, China; 3 NMPA Key Laboratory for Research and Evaluation of Drug Metabolism, Department of Pharmacy, Pingshan Hospital, Southern Medical University, Shenzhen, China; 4 State Key Laboratory of Multi-organ Injury Prevention and Treatment, MOE Key Laboratory of Infectious Diseases Research in South China, MOE Innovation Center for Medical Basic Research on Inflammation and Immune Related Diseases, Southern Medical University, Guangzhou, China; Shenzhen University, CHINA

## Abstract

Guanylate-binding proteins (GBPs) are immune GTPases induced by interferons (IFNs) and comprise GBPs1-7. In this study, GBP7, a less studied member of the GBPs, was identified to possess Herpes simplex virus I (HSV-1) promoting effects on replication both in vitro and in vivo. Knockdown of GBP7 or loss of its GTPase activity demonstrated significant antiviral effects. Consistently, GBP7^-/-^ mice infected with HSV-1 showed higher survival and milder symptoms. Mechanistically, we discovered GBP7 directly binds to eukaryotic translation elongation factor 1α1 (eEF1A1), which was validated through immunoprecipitation mass spectrum (IP-MS) and binding affinity assays. GBP7 utilizes its GTPase activity to assist eEF1A1 in GTP hydrolysis, activating its role in promoting protein translation, thereby increasing viral replication and infection. Moreover, through high-throughput virtual screening, S-Adenosylhomocysteine (SAH) was identified as a novel GBP7 targeting inhibitor, which demonstrated potent antiviral activity against HSV-1infection and broad-spectrum potentiality. Collectively, these findings uncover the critical role of the GBP7-eEF1A1 axis in viral infection and provide theoretical basis for host-targeted antiviral therapy.

## Author summary

Guanylate-binding proteins (GBPs) are recognized as integral components of the IFN-γ-induced defense system against pathogenic microbial infections, particularly human GBPs1-7. In our study, it was discovered that GBP7 was highly expressed in virus-infected cells and to a certain extent promoted the infection process which indicated a host factor facilitating viral infection and a potential antiviral drug target. Protein interaction research and enzyme activity detection demonstrated that GBP7 was highly probable to facilitate the translation elongation function of eEF1A1 by leveraging its GTPase activity. The interaction mode and key binding sites between eEF1A1 and GBP7 in this study provided insights of the protein structure of the complex. Additionally, we discovered the broad antiviral activity of the compound SAH and identified GBP7 as a novel host target for the rational design of broad-spectrum antiviral inhibitors.

## Introduction

Herpes simplex viruses (HSV) have a high prevalence in the human population. After primary infection, the virus remains latent in sensory ganglia but can sporadically reactivate typically causing lesions at or near the primary infection site [1]. To date, the majority of useful herpesvirus antivirals are nucleoside analogues that block herpesvirus DNA replication by targeting the DNA polymerase. The escalating issue of drug resistance to anti-herpesvirus agents has become increasingly prominent, encompassing varying degrees of resistance observed in HSV-1 and HSV-2 infections against FDA-approved medications such as acyclovir, valacyclovir, famciclovir (the oral prodrug of penciclovir), as well as second-line agents including foscarnet and cidofovir [2–4]. Most approved drugs primarily target the genetic material or enzymes (direct-acting antivirals, DAAs) of the viruses [5], making it easy for such drugs to select drug-resistant viral variants [6]. In contrast, HDT approaches, such as DAS181 and leflunomide [7,8], offer advantages in overcoming drug resistance and effectively combating multiple viruses (including emerging ones), presenting a promising strategy for developing antiviral drugs [9].

GBPs, a family of dynamin-related large GTPases, are expressed in response to IFNs or pathogen infection [10]. Previous studies have identified seven human and eleven mouse GBPs, highlighting their central involvement in bacterial and pathogen inflammasome activation [11]. GBP1 has been demonstrated to restrict the replication of various RNA viruses through its GTPase activity [12], whereas GBP2 inhibits EMCV and MNV replication by binding to GTP [13]. Additionally, GBP5 impairs envelope glycoprotein processing, thereby inhibiting the infection of respiratory syncytial virus (RSV) and human immunodeficiency virus type I (HIV-1) [14]. Collectively, these studies suggest that GBPs play a significant role in the host antiviral immune system. The novel roles of GBPs in viral infection necessitate a more comprehensive understanding. In this study, we identified GBP7, a less studied type of GBP present [15], which differs from other members with already studied antiviral effects but exhibits unique virus-promoting effects on viral replication and infection [16,17]. We discovered a novel biological function of GBP7 in the protein translation process within cells and applied it to the infection processes of a wider variety of viruses.

Eukaryotic translation elongation factors (eEFs) play an essential role in protein biosynthesis during the elongation step of translation [18]. Human eukaryotic translation elongation factor 1α (eEF1A) is responsible for delivering aminoacylated tRNAs to the A site of the ribosome for mRNA decoding via codon-anticodon interactions [19,20]. In addition, accumulating evidence suggests that eEF1A has multifunctional roles, including its role as a protein chaperone and its ability to bind RNA and actin, which enable it to function as a cofactor in viral replication [21–23]. Notably, eEF1A has emerged as an indispensable host factor across multiple viral pathogens, such as infectious gastroenteritis coronavirus, severe acute respiratory syndrome coronavirus (SARS-CoV), and RSV [24,25]. Furthermore, plitidepsin has demonstrated acceptable safety in clinical Phase I/III trials, indicating the inhibition of eEF1A pathway has emerged as a promising avenue for achieving antiviral efficacy [26–28]. Using IP-MS and complementary biochemical approaches, we identified eukaryotic translation elongation factor 1α 1 (eEF1A1) as a novel GBP7-interacting protein that functions as a critical cofactor for viral replication.

The present study reveals that the crucial role of GBP7-eEF1A1 complex in promoting viral infection through a positive feedback mechanism. To observe the performance of GBP7 in antiviral immunity, we employed HSV-1 together with the nucleic acid analogs polyinosinic-polycytidylic acid (poly I:C) and poly (dA:dT), which mimic viral RNA and DNA, respectively. The functional role of GBP7 in HSV-1 infection was explored through gene knockout, both *in vivo* and *in vitro*. Through IP-MS and binding affinity assays, we identified and validated eEF1A1 as a direct interactor of GBP7. Moreover, the importance of GTPase activity and the structural information of the interaction between eEF1A1 and GBP7 were further elucidated by constructing a series of truncated and mutant GBP7 proteins. These findings demonstrate that viruses exploit host immune responses to activate GBP7-eEF1A1 axis, thereby enhancing protein translation to promote viral replication and infection. In addition, we investigated the role of GBP7 in a broader spectrum of viral infection processes and highlighted the advantages of GBP7 as a potential target for broad-spectrum antiviral drugs.

## Results

### HSV-1 infection activates the transcription and expression of GBP7

Our previous studies have revealed that GBP7 inhibits the signaling pathways NF-κB and JAK-STAT during influenza virus infection [17]. To investigate whether GBP7 functions similarly across distinct viral pathogens, we established HSV infection models. We firstly observed a substantial increase in GBP7 in cells infected with HSV-1, which was positively correlated with the duration of infection, as indicated by the increase in mRNA levels (Fig 1A), protein content (Fig 1B), and fluorescence intensity (Fig 1C). Similar GBP7 gene expression activation was observed in Vero and HeLa cells (S1A Fig), as well as in A549 cells infected with a gradient of viral multiplicities of infection (MOI; low, medium, and high) (S1B Fig). *In vivo* studies (Fig 1D) further revealed a consistent phenotype, in which elevated protein expression (Fig 1E) and higher mRNA levels (Fig 1F) of GBP7 were detected in the trigeminal nerve of mice infected with HSV-1, compared to control samples. The immunofluorescence (IF) assessment yielded concordant results (Fig 1G). The massive activation of GBP7 both *in vivo* and *in vitro* suggests that it may play a critical role in HSV-1 infection.

**Fig 1:**
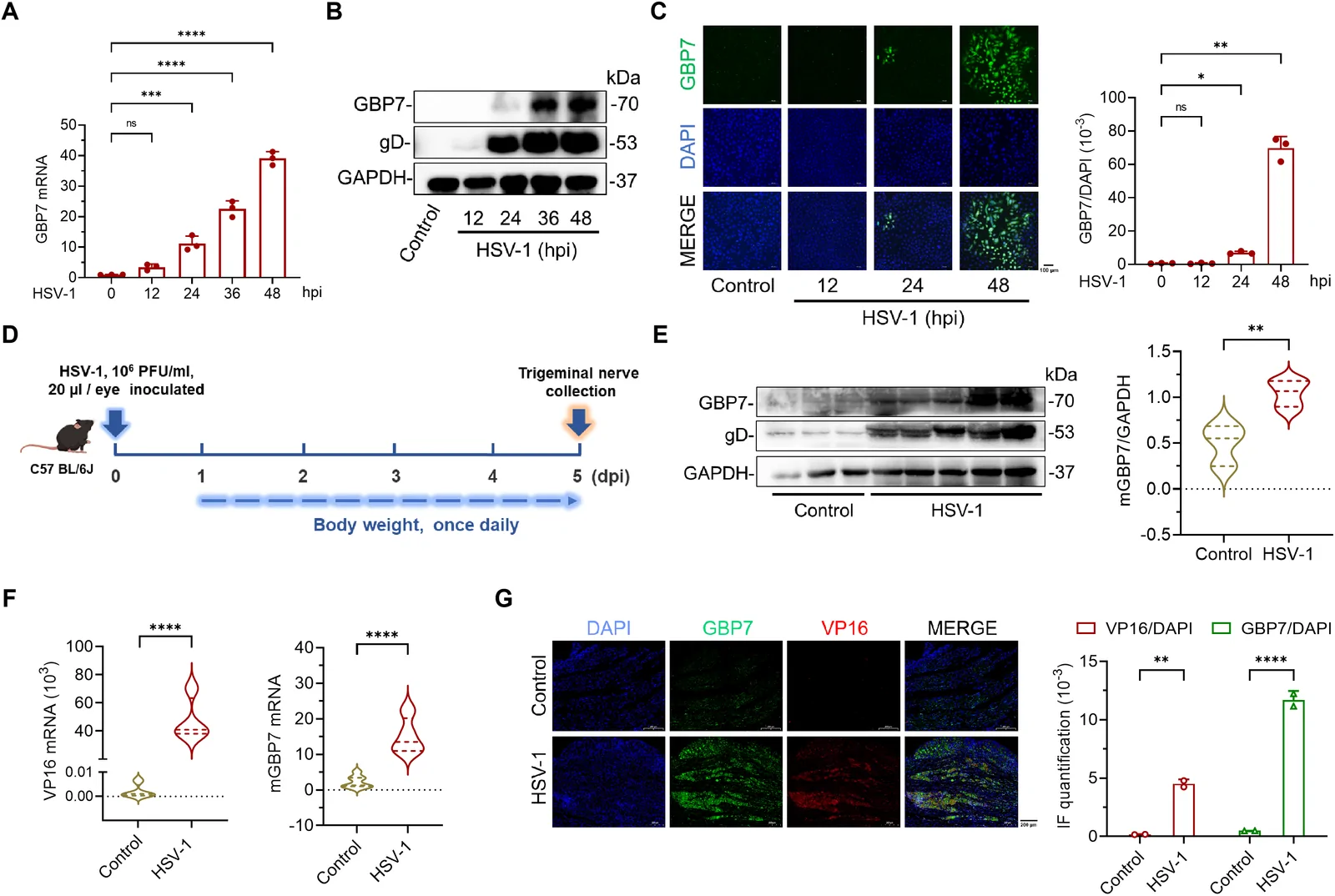
Infection with HSV-1 activates the transcription and expression of the GBP7 gene. **(A, B)** RT-qPCR and western blotting analysis of GBP7 in A549 cells infected with HSV-1 at 0, 12, 24, 36 and 48 h, MOI = 0.1. **(C)** Immunofluorescence staining of GBP7 (green) and DAPI (blue) in A549 cells infected with HSV-1 virus. Scale bars, 100 μm. **(D)** Flowchart of HSV-1 infection model. C57BL/6J mice were inoculated with 10⁶ PFU/ml HSV-1 (20 μl per eye) on scratched corneas. **(E, F)** Reverse transcription-PCR (RT-PCR) and western blotting (WB) analysis of GBP7 in trigeminal nerve of mice on Day 5 (Control group, n = 3; HSV-1 group, n = 5) and quantification. **(G)** The trigeminal nerve sections of mice infected with HSV-1 for 5 days were stained by immunofluorescence. Red: VP16; Green: GBP7; Blue: DAPI. Scale bars, 200 μm. All data are shown as means ± SEM. Three or more independent experiments were performed. Statistical significance was assessed by one-way ANOVA in **A, C** and t-test in **E**, **F**. *ns*, not significant; **P < 0.01; ***P < 0.001; ****P < 0.0001.

### GBP7 promotes HSV-1 replication and infection *in vitro*

To elucidate the role of GBP7 in HSV-1 infection, we overexpressed GBP7 in target cells and displayed the expression effect with Flag tags (Fig 2A). GBP7 overexpression markedly increased HSV-1 gD protein levels over the same infection course (Fig 2B). Consistently, viral mRNA transcripts, including those encoding VP16, ICP8, and UL30, were significantly upregulated (Fig 2C). We then quantified the viral load in both cell lysates and culture supernatants, along with the determination of IF analysis (S2 Fig). GBP7 overexpression resulted in a considerable accumulation of viruses and a higher number of progeny viruses (Plaques assay) released into the supernatant (Fig 2D). Furthermore, we observed similar outcomes when GBP7 was overexpressed in HSV-1- and HSV-2-infected epithelial Hela and Hacat cells (Figs 2E and 2F).

**Fig 2:**
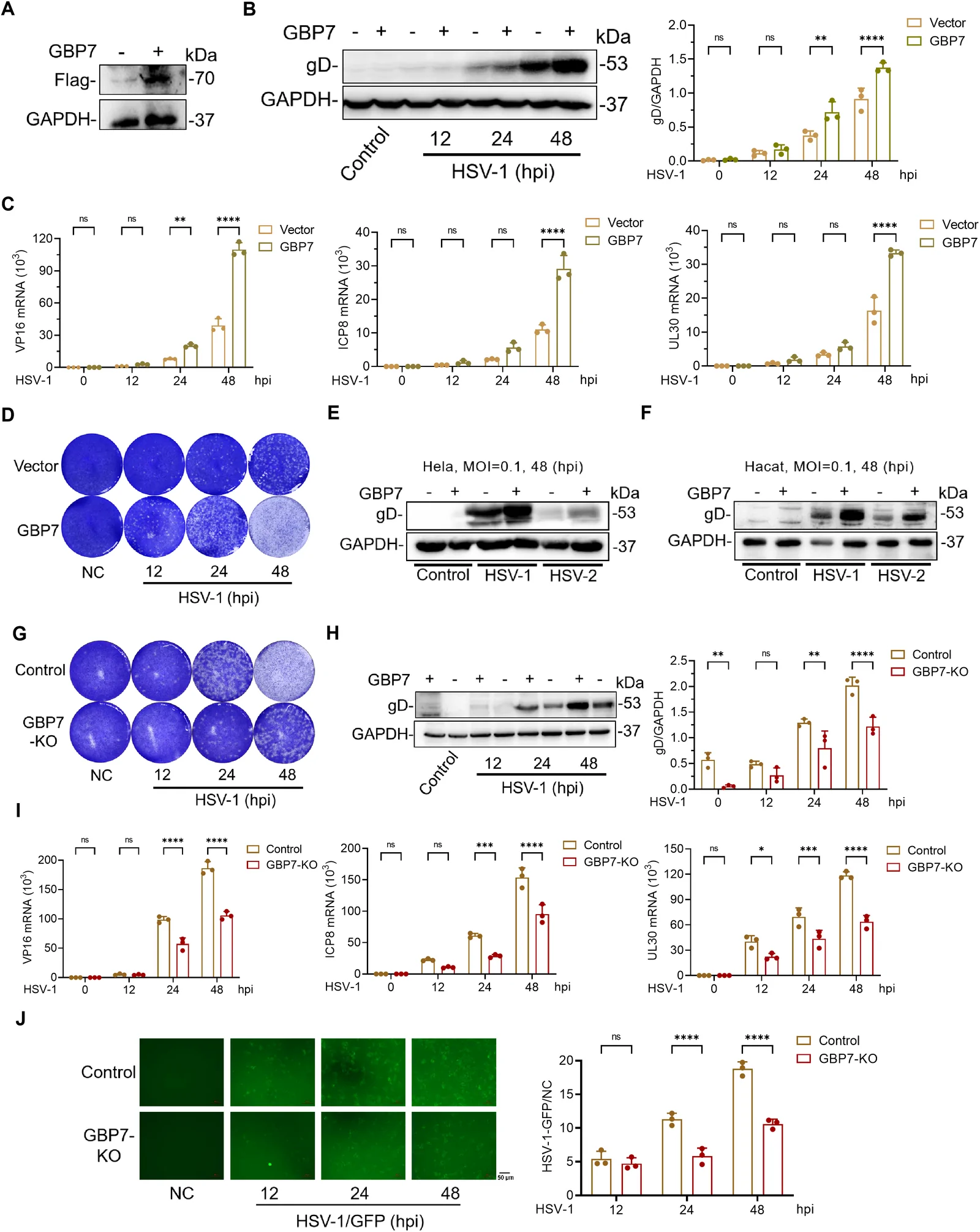
GBP7 acts as a key promoter of HSV-1 viral replication and infection. **(A)** The GBP7 protein level in A549 cells transfected with the Flag-tagged overexpression plasmid for 24 h. **(B, C)** A549 cells overexpressing GBP7 or vector control were infected with HSV-1 (MOI = 0.1) for 12, 24, and 48 **h.** Western blotting analysis of gD protein **(B)**. RT-qPCR analysis of VP16, ICP8 and UL30 mRNA levels **(C)**. **(D)** Plaque assays of progeny virus titers in culture supernatants from **(B, C)**. **(E, F)** Western blotting analysis of gD protein in Hela and Hacat cells overexpressing GBP7 at 48 h after HSV-1 infection. **(G)** Plaque assays in cell culture supernatant at 12, 24 and 48 h after infection between GBP7-KO and control group. **(H, I)** GBP7-KO and control cells were infected with HSV-1 (MOI = 0.1) for 12, 24, and 48 h. Western blotting analysis of gD protein levels **(H)**. RT-qPCR analysis of VP16, ICP8 and UL30 mRNA levels **(I)**. **(J)** Fluorescence image of GBP7-KO cells infected with GFP-tagged HSV-1 at 12, 24 and 48 h. Scale bars, 50 μm. Data are presented as means ± SEM from three independent experiments. Statistical significance was assessed by two-way ANOVA. *ns*, not significant; *P < 0.05; **P < 0.01; ***P < 0.001; ****P < 0.0001.

Subsequently, the antiviral effect of GBP7 deficiency was confirmed. We designed three distinct sg-RNAs to establish and generated monoclonal cell lines with GBP7 knockout (GBP7-KO) (S3A Fig). The GBP7-KO cells exhibited comparable cell proliferation rates to wild-type (WT) cells (S3B Fig), confirming that GBP7 ablation does not affect cell growth. In contrast to the findings observed in the overexpression results, GBP7-KO significantly reduced progeny virus production, viral protein expression and mRNA levels in infected cells (Figs 2G-2I). Furthermore, at various time points post-infection, GBP7-KO resulted in a reduction in viral accumulation in cells, as evidenced by IF assays (S4 Fig) and infection with green fluorescent protein (GFP)-tagged HSV-1 (Fig 2J).

### Mice deficient in GBP7 gene exhibited milder symptoms and higher survival rates during HSV-1 infection

To evaluate the potential antiviral effect of GBP7-KO *in vivo*, we generated a GBP7-knocked out C57BL/6J mice (GBP7^-/-^) via CRISPR/Cas-mediated genome engineering [29] (S5A Fig) and screened out the homozygosity through the self-crossing of heterozygous F1 generations via DNA page (S5B Fig). Compared with WT mice, four-week-old GBP7^-/-^ mice exhibited deletion of the GBP7 gene in the cornea, trigeminal nerve, and periocular skin tissues, with no differences in body posture and organs such as the heart, liver, spleen, lungs and kidneys were observed (S5C to S5E Figs).

GBP7^-/-^ and WT mice were subsequently subjected to corneal infection with the HSV-1 M strain [30] (Fig 3A). As shown in Figs 3B and 3C, despite comparable body weights between genotypes, GBP7^-/-^ mice exhibited significantly improved survival following HSV-1 infection. Notably, GBP7 knockout effectively reduced viral infection and eye injuries of infected mice (Fig 3D), as indicated by HSV-1 viral titers of eye irrigation solution which was quantified by plaque assays (Figs 3E and 3F). Viral loads in the trigeminal nerves were further quantified by western blotting and RT-PCR, revealing substantially reduced viral protein and mRNA levels in GBP7^-/-^ mice relative to WT controls over the same infection course (Figs 3G and 3H). Histological analysis by immunohistochemistry (IHC) revealed attenuated inflammatory cell infiltration in the cornea, trigeminal nerve, and periocular skin tissues in GBP7^-/-^ mice at 5 or 7 days after infection (Figs 3I to 3K). Furthermore, IF were employed to localize the expression of viral proteins in infected tissues. It was indicated the VP16 protein levels in the trigeminal nerve were greater in WT mice than in GBP7^-/-^ mice (S6 Fig). Collectively, these findings suggest that GBP7 knockout confers resistance to HSV-1 infection and improves survival, prompting further investigation into its precise role.

**Fig 3:**
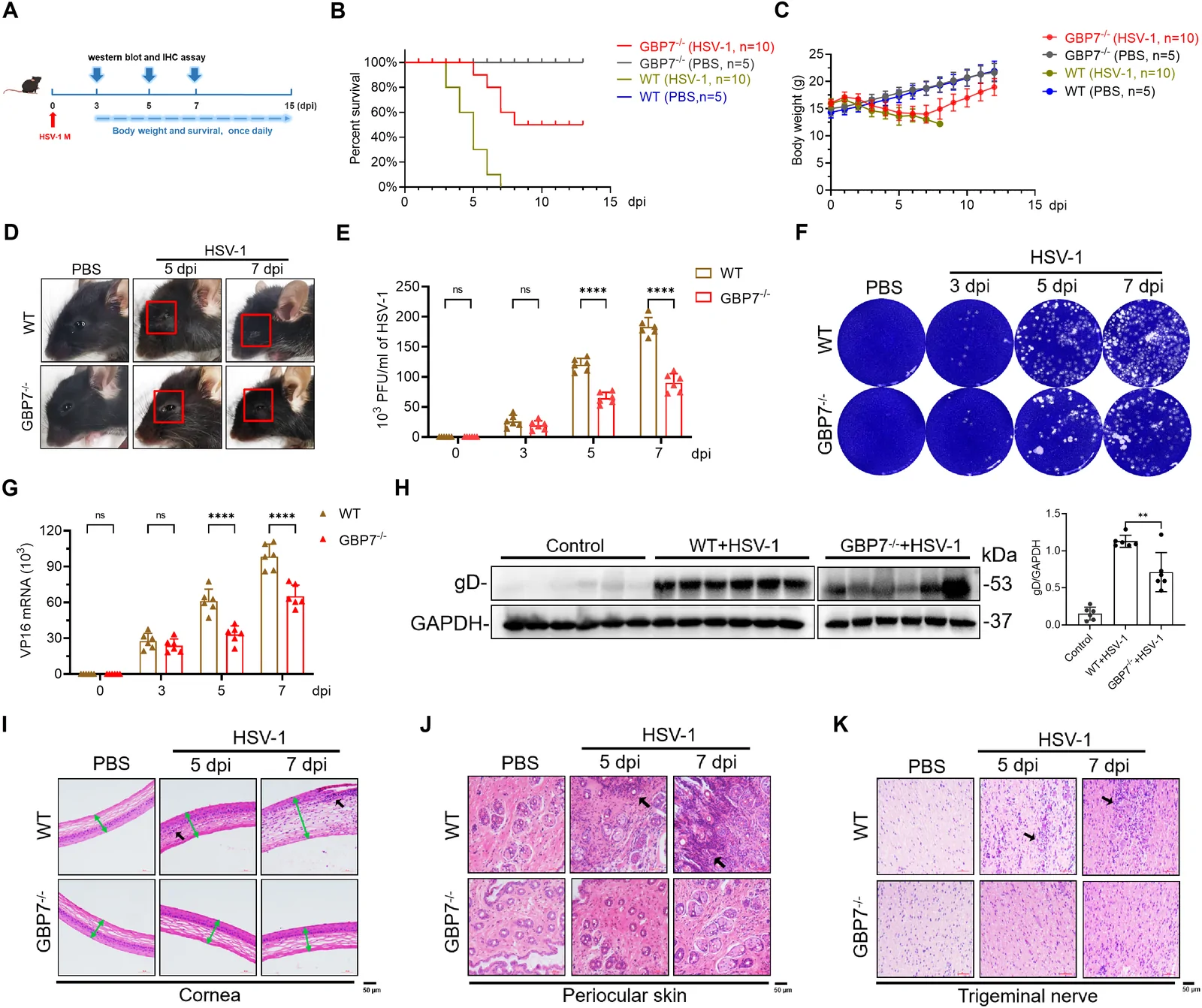
GBP7 knockout mice showed stronger resistance to HSV-1 infection *in vivo.* **(A)** WT and GBP7^-/-^ mice were inoculated with mice adapted strain HSV-1 M (10^6^ PFU/ml). HSV-1 group, n = 10; PBS control group, n = 5. **(B, C)** Body weight were measured daily. Survival was analyzed by the Kaplan–Meier method with log-rank test. **(D)** Representative images of periocular herpes-like lesions on 5 and 7 dpi. **(E, F)** The eyeballs of infected mice were repeatedly rinsed with 100 μl sterile PBS and the virus titer was calculated by plaque assay. **(G)** RT-qPCR to quantitatively determine the viral loads in the trigeminal nerve of HSV-1 M infected mice on 3, 5, and 7 dpi. **(H)** Western blotting was used to detect the gD protein in trigeminal nerves at 7 dpi and quantification was analyzed by Image **J. (I-K)** H&E staining of cornea, periocular skin, and trigeminal nerves of HSV-1 M infected WT and GBP7^-/-^ mice, scale bars: 50 µm. All data are shown as means ± SEM. Three or more independent experiments were performed. Statistical significance were assessed by two-way ANOVA in **E**, **G** and t-test in **H**. *ns*, not significant; **P < 0.01; ****P < 0.0001.

### Identification of eEF1A1 interacted with GBP7 to collaboratively promote HSV-1 infection

To investigate the mechanism by which GBP7 regulates virus infection, we performed co-immunoprecipitation (CO-IP) combined with mass spectrometry (MS) analyses (Fig 4A). This proteomic approach identified 318 potential GBP7-Flag binding partners, of which 163 were nonspecific associated with the IgG control (Figs 4B and 4C). As a result of the enriched Gene Ontology (GO) terms in the biological process (BP) group and KEGG pathway analysis, we focused on candidate proteins related to translation in 155 proteins specificly interacted with GBP7 (Figs 4C and 4D, S7A Fig). The top 28 proteins ranked by peptide-spectrum matches (PSMs) (S1 Table) were mapped onto a protein-protein interaction (PPI) network, revealing no previously reported interactions involving GBP7 (S7B Fig). Notably, eEF1A1, an essential elongation factor that delivers aminoacyl-tRNA to the ribosome and has been implicated in the life cycles of multiple viruses [26,31], emerged as a prominent candidate. Further immunofluorescence staining revealed extensive colocalization of eEF1A1 and GBP7, with increased protein levels observed in virus-infected cells (Fig 4E). Additionally, CO-IP assays in HSV-1-treated cells revealed that eEF1A1 and GBP7 coimmunoprecipitated with each other, and that viral infection upregulated the complex formed by both proteins (Figs 4F and 4G). Similar observations were made for exogenous eEF1A1 in complex with GBP7 (S7C and S7D Figs).

**Fig 4:**
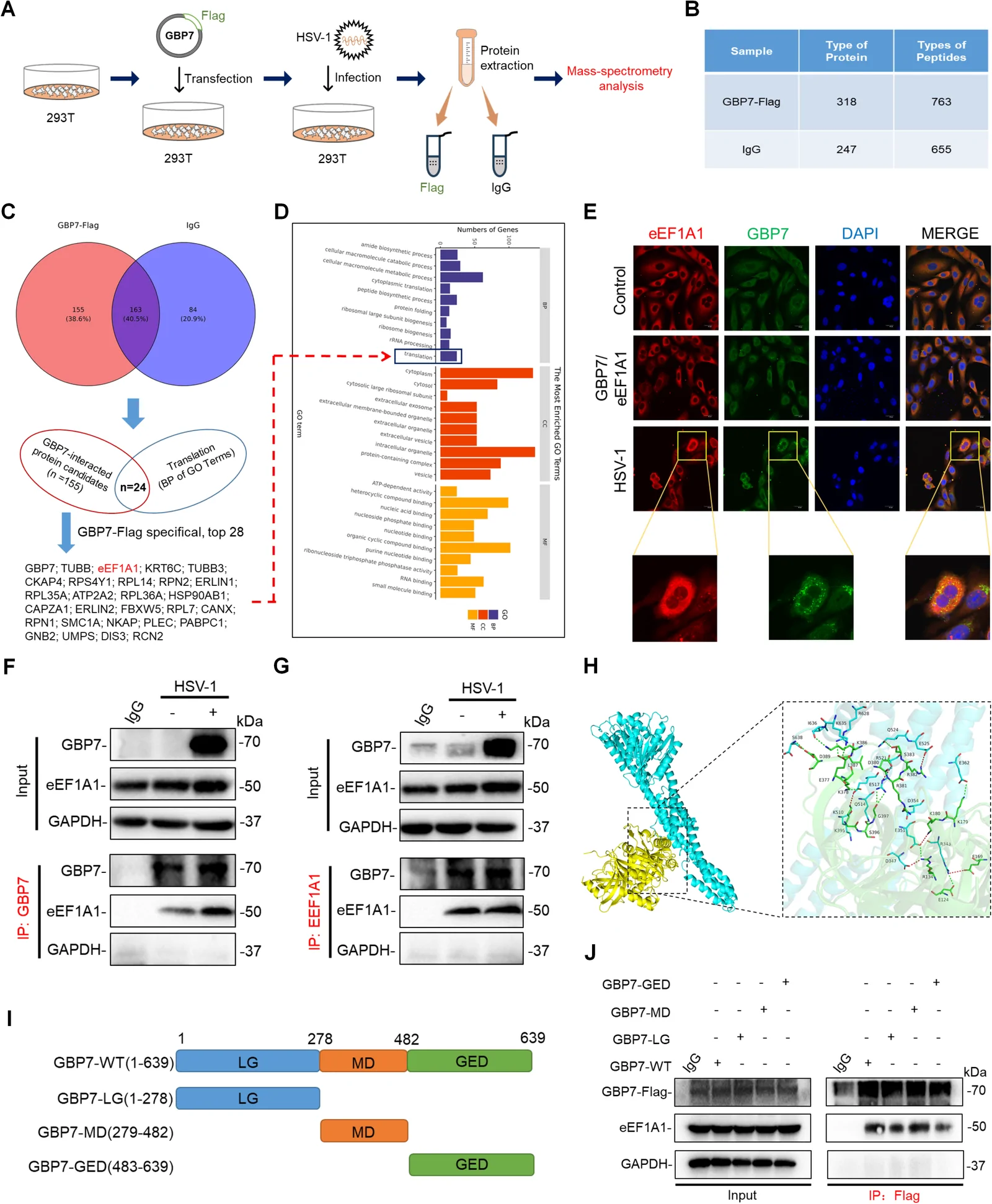
Identified the binding and interaction between GBP7 and eEF1A1. **(A, B)** Experimental flowchart for the identification of 318 proteins interacted with GBP7-Flag through IP-MS. **(C)** 155 proteins interact with GBP7 excluded from non-specific results. **(D)** Gene Ontology (GO) analysis of the appraised results. **(E)** Fluorescence images of GBP7, eEF1A1 and DAPI labeled in Vero cells transfected with plasmids or infected with HSV-1. **(F, G)** Co-immunoprecipitation analysis of eEF1A1 and GBP7 proteins in HSV-1 infected cells. **(H)** Molecular docking by HDOCK to predict the interaction model and binding site between eEF1A1 and GBP7. **(I, J)** Design of the LG, MD, GED truncated GBP7 proteins and the CO-IP analysis of their interaction with eEF1A1.

Molecular docking of eEF1A1 and GBP7 (AF2 homology modeling) was performed using the HDOCK program (S7E and S7F Figs), resulting in a reliable complex model with a docking score of -221.50 kcal/mol and a confidence score of 0.8069 (Confidence score higher than 0.7: highly likely to bind) [32,33] (Fig 4H). To determine which domain of GBP7 binds to eEF1A1, three truncated forms of GBP7, including the large G domain (LG, residues1–278), middle domain (MD, residues 279–482), and GTPase effector domain (GED, residues 483–639), were constructed on the basis of previous studies on the structure [34–37] and molecular docking models of GBPs (Fig 4I). Consistent with the docking model, the MD region exhibited the strongest binding affinity for eEF1A1 (Fig 4J). Consequently, proteins with simultaneous mutations of 12 amino acids and single-point mutations were employed to investigate the key residues for the binding of GBP7 and eEF1A1 (S7G and S7H Figs). The results demonstrated that residues R343, E362, and K510 were critical for eEF1A1-GBP7 complex formation (S7I Fig).

We further elucidated the mechanism of action of GBP7-eEF1A1. Overexpression of eEF1A1 or GBP7 individually enhanced viral replication, whereas their co-expression produced a synergistic proviral effect, as evidenced by increased HSV-1 gD protein accumulation from 12 h post-infection (S8A Fig). Since eEF1A1 has been shown to promote virus infection via different mechanisms [26,38,39], we silenced the eEF1A1 gene through si-RNA in cells and reintroduced GBP7 (S8B Fig). Notably, interference with eEF1A1 expression not only directly suppressed viral infection but also abolished the original ability of GBP7 to promote viral infection (S8C Fig). The above results suggest a HSV-1 viral infection regulatory axis composed of eEF1A1 and GBP7.

### The GTPase GBP7 activates eEF1A1 to accelerate protein translation during HSV-1 infection

The hydrolysis of GTP is an essential process for eEF1A1 to transport tRNA, which requires the participation of a GTPase [40–42]. Consequently, as GBP7 is an interacting protein of eEF1A1, its GTPase activity has drawn our attention. Some earlier investigations have suggested that the R48 and K51 sites are crucial elements for the GTPase activity of GBPs [36,43–45]. To validate their functional importance, we generated alanine-substitution mutants at these positions (GBP7^R48A^ and GBP7^K51A^) (Fig 5A). Recombinant GBP7 and its two mutant proteins were expressed and purified, and verified via SDS-PAGE (Fig 5B). By using an ATPase/GTPase activity assay kit, we found that the GTPase activities of the GBP7^R48A^ and GBP7^K51A^ were significantly lower than that of the GBP7^WT^ (Fig 5C). During HSV-1 viral infection, a reduction in the GTP content was observed with exogenous overexpression of GBP7, whereas no such phenomenon was detected in the R48 and K51 mutants (S9A Fig). Conversely, GBP7^WT^ significantly elevated intracellular GDP and GMP levels compared with the mutants (S9B and S9C Figs). In an infection with a temporal gradient, differences were observed in the contents of GTP, GDP, and GMP in GBP7-KO cells compared to those in the controls, indicating a higher concentration of GTP and lower concentrations of GDP and GMP (S9D to S9F Figs). These data indicate that GBP7 possesses relatively high GTPase activity to promote the hydrolysis of GTP in infected cells, thereby increasing GDP and GMP production.

**Fig 5:**
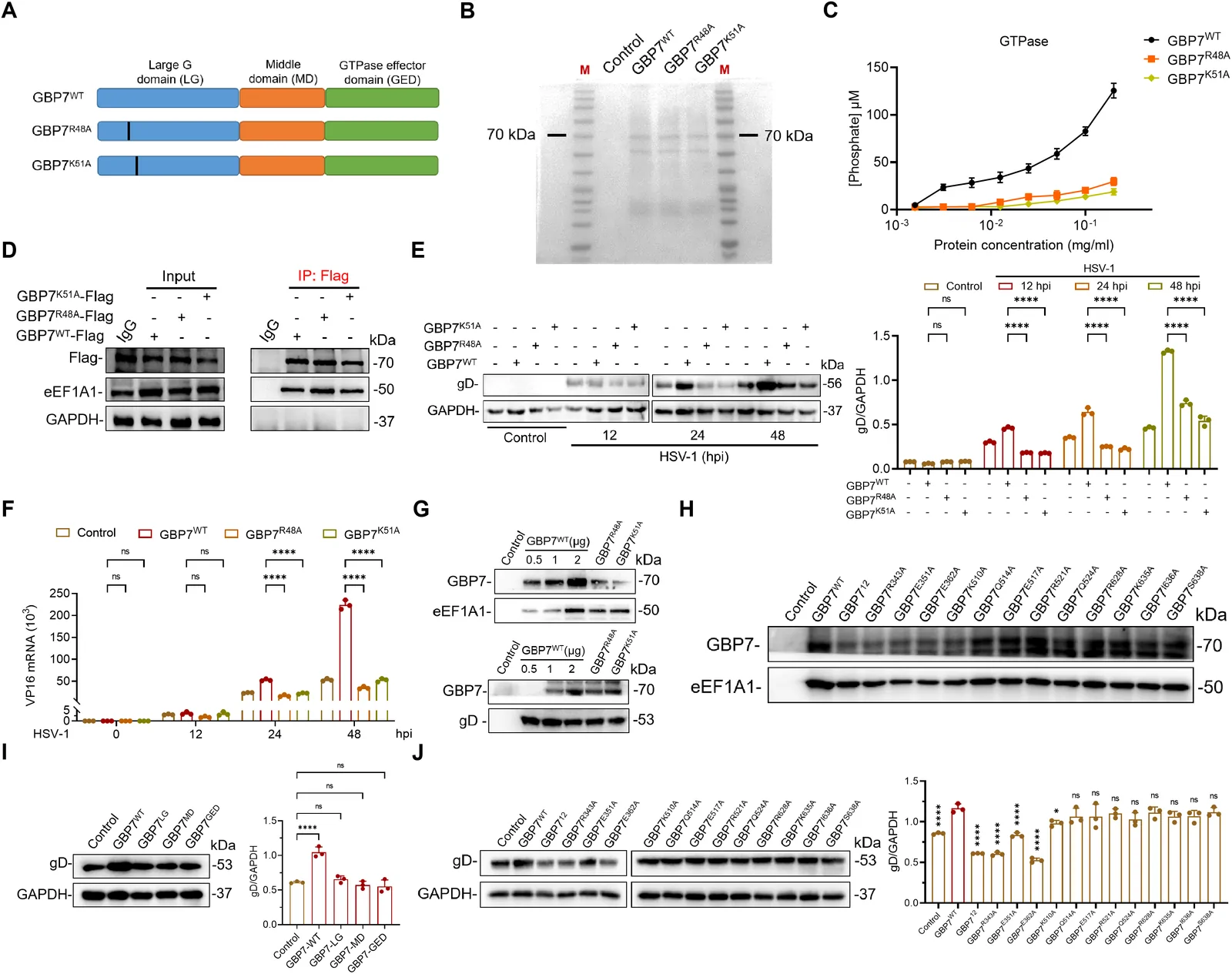
GTPase GBP7 activates eEF1A1 to promote protein translation in HSV-1 infection. **(A, B)** Expression of GBP7 and its R48, K51 mutant proteins were verified by SDS-PAGE. **(C)** GTPase activity of the GBP7^WT^, GBP7^R48A^ and GBP7^K51A^ measured by ATPase/GTPase Activity Assay Kit. **(D)** CO-IP analysis of the interaction between GBP7 ^WT^, GBP7^R48A^, GBP7^K51A^ and eEF1A1. **(E, F)** GBP7 and its R48, K51 mutants were expressed in A459 cells and their effects on HSV-1 infection were compared through western blotting and RT-qPCR. Quantified by Image **J. (G)** Different amounts of GBP7 plasmids (0.5; 1; 2 μg) or mutant plasmids (1 μg) with 1µg eEF1A1 or gD plasmids were added to the TnT Quick Coupled Transcription/Translation System for in vitro translation, assessed by Western blotting. **(H)** Effect of GBP7 mutants on eEF1A1-mediated in vitro translation. GBP7^WT^, GBP7^12^ (12-sites mutant), and single-site mutants (R343A, E351A, E362A, K510A, Q514A, E517A, R521A, Q524A, R628A, K635A, I636A, S638A) were introduced into the translation system, along with eEF1A1 (1 μg). **(I, J)** Western blot analysis of HSV-1 gD in cells expressing truncated and mutant GBP7 at 24 h post-HSV-1 infection. All data are shown as means ± SEM. Three independent experiments were performed. Statistical significance was assessed by two-way ANOVA in **E**, **F** and one-way ANOVA in **I**, **J**. *ns*, not significant; *P < 0.05; ****P < 0.0001.

Based on the mutations at R48 and K51, which suppress the GTPase activity of GBP7 in cells, we investigated whether these mutations affected its ability to promote viral infection. Under the premise of not interfering with its binding to eEF1A1 (Fig 5D), the results indicated that only GBP7^WT^ increased the HSV-1 gD levels (Fig 5E). RT-qPCR assays also revealed that GBP7 protein with mutations at the R48 and K51 sites was no longer capable of promoting HSV-1 viral infection (Fig 5F). Next, we used an *in vitro* protein translation system to investigate the effects of GBP7, including its mutants, and eEF1A1 on the protein translation process. The concurrent addition of the GBP7^WT^ and eEF1A1 plasmids resulted in increased protein translation within the same time frame, as detected by western blotting. However, when both GBP7 and the gD plasmid were added but eEF1A1 was absent, the translation-promoting function of GBP7 could not be achieved. (Fig. 5G). When certain amino acid mutations of GBP7 result in a reduction of a weakened binding capacity with eEF1A1, the influence on translation function is also mitigated (Fig 5H).

Co-IP analysis further confirmed that GBP7^R48A^ and GBP7^K51A^ retained eEF1A1-binding capacity, revealed that GTPase activity was the key factor by which GBP7 promoted viral infection (Figs 5D and 5E). Consistently, it was also confirmed that the GBP7 LG, MD and GED truncated protein, which bound to eEF1A1 but lacked functional GTPase activity, failed to affect viral infection (Fig 5I). Furthermore, GBP7 point mutants with attenuated eEF1A1 binding, including R343A, E362A, and the 12-site combinatorial mutant, exerted minimal effects on viral infection (Fig 5J). Collectively, GBP7, which binds to eEF1A1, harnesses its intrinsic GTPase activity to stimulate the biological function of eEF1A1, thereby promoting protein translation and ultimately facilitating viral replication and infection. These findings also position GBP7 as a potential target for the development of broad-spectrum antiviral drugs.

### Identification of SAH as a GBP7-targeting agent that inhibits HSV-1 viral infection

To elucidate the therapeutic potential of targeting GBP7, we performed high-throughput virtual screening for small-molecule inhibitors. Leveraging the structure prediction model of GBP7 (AlphaFold ID: AF-Q8N8V2-F1), we defined the GTPase active site encompassing residues 45–52, 67–69, and 97–101 as the docking pockets (Fig 6A). From a comprehensive library of approximately 20,000 compounds (TargetMol active and natural product libraries), 1280 conformations were screened out followed by an evaluation of the correlation between scoring and molecular weight (S10A Fig), as well as an analysis of Protein Ligand Interaction Fingerprints (PLIF) (S10B Fig). Following structural diversity analysis and activity comparison, we focused on a promising small molecule S-Adenosylhomocystein (SAH; TQ0208) (S2 Table, S10C and S10D Figs). To validate the interaction between SAH and GBP7, drug affinity responsive target stability (DARTS) assays demonstrated that SAH exhibited preferential binding affinity for GBP7^WT^ over the GBP7^R48^ or GBP7^K51^ mutants (Fig. 6B and S11 Fig). In parallel, surface plasmon resonance (SPR) assays confirmed the direct binding of SAH to GBP7 *in vitro* (Fig. 6C).

**Fig 6:**
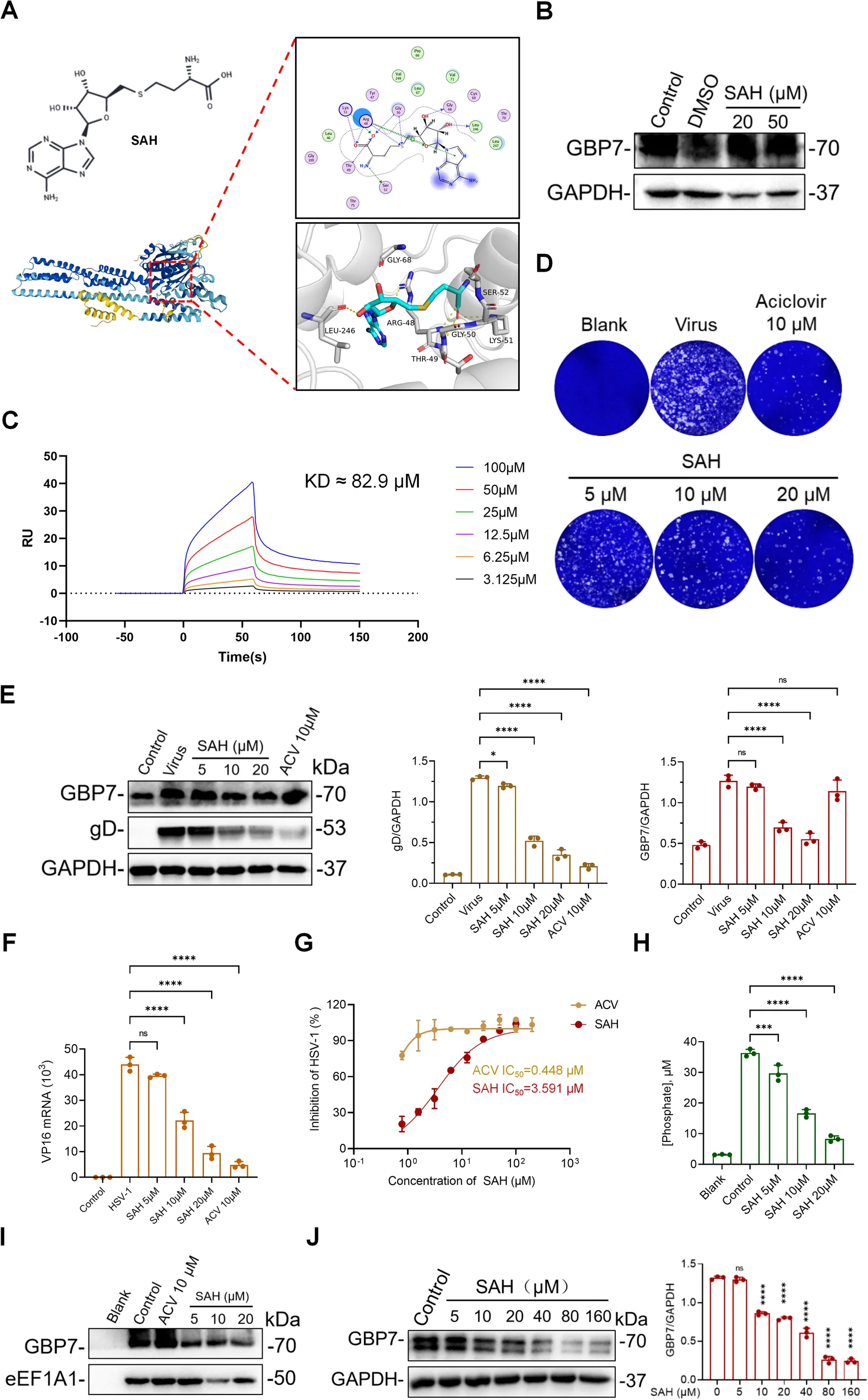
Screening of anti-HSV-1 virus compounds targeting GBP7. **(A)** Molecular docking structure image and binding site information of compound SAH and GBP7 protein (AlphaFold ID: AF-Q8N8V2-F1). **(B)** Western blotting detection of affinity between SAH and GBP7 protein through DARTS assay. **(C)** SPR detection results of SAH compounds with concentration gradient and GBP7 protein. **(D)** Plaque assay was used to quantify the viral titers in the supernatant of A549 cells infected with HSV-1 under 5, 10, 20μM concentrations of SAH treatment, and as Acyclovir was used as positive control. **(E, F)** Western blotting and RT-qPCR were used to determine the effect of SAH (5, 10, 20 μM) and ACV (10 μM) on the viral protein and mRNA levels in cells at 24 hours after HSV-1 infection. **(G)** CCK8 assay was used to detect the IC_50_ of SAH and ACV on A549 cells against HSV-1 infection (ACV: 0.448 µM; SAH: 3.59 µM). **(H)** ATPase/GTPase Activity Assay Kit to detect the GTPase activity of the GBP7. **(I)** SAH inhibited protein translation in TnT Quick Coupled Transcription/Translation System. **(J)** Western blotting analysis of expression of GBP7 in A549 cells. Quantification was analyzed by Image **J.** All data are shown as means ± SEM. Three independent experiments were performed. Statistical significance was assessed by one-way ANOVA. *ns*, not significant; *P < 0.05; ****P < 0.0001.

In HSV-1 infected cells, SAH exhibited antiviral activity comparable to that of acyclovir (ACV) at the concentration of 10 μM (Figs 6D and 6E), as evidenced by reduced VP16 mRNA levels (Fig 6F) and the IC₅₀ values of 3.59 µM against HSV-1 infection (Fig 6G). Importantly, SAH exhibited minimal cytotoxicity across A549, Vero and Hela cells at concentrations below 200 µM (S12 Fig). To further verify the effect of SAH on the biological functional activity of GBP7, we found that SAH reduces protein translation efficiency by suppressing the GTPase activity of GBP7 in a concentration-dependent manner (Figs 6H and 6I). This is consistent with our previous results from molecular docking. Notably, we also observed that as the concentration of SAH gradually increased, the content of GBP7 protein showed a significant downward trend (Fig 6J). However, ACV did not exhibit this effect (S13 Fig). These findings indicated that as a targeted inhibitor of GBP7, SAH not only downregulates the expression of GBP7 protein in cells but also impairs the GTPase activity of GBP7, thereby abrogating its function to active eEF1A1 to facilitate protein translation during HSV-1 infection.

Furthermore, in mouse model of HSV-1 infection, we evaluated the therapeutic effect of high (200 mg/kg), medium (50 mg/kg) and low (5 mg/kg) doses of SAH (Fig 7A). After virus infection, mice were intragastrically administered (IG) with PBS and 50 mg/kg ACV as negative and positive controls, respectively. By daily recording the weight and survival of mice, it was found that the survival rate of mice infected with HSV-1 was significantly improved by SAH at medium and high doses, which were increased to 50% and 70%, respectively (Figs 7B and 7C). HE staining results of periocular skin, cornea and trigeminal nerve infected with HSV-1 virus for five days also showed that SAH (200mg/kg) could significantly reduce the inflammatory infiltration and physiological lesions (Figs 7D to 7F). Further detection of the viral load in eye lavage fluid (Fig 7G) and trigeminal nerve showed that in tissues of mice treated with medium or high doses of SAH decreased to varying degrees through RT-qPCR and Plaque Assays (Figs 7H and 7I). The in vivo experimental results indicate that SAH has the potential to serve as a host targeted therapeutic agent for HSV-1 infection.

**Fig 7:**
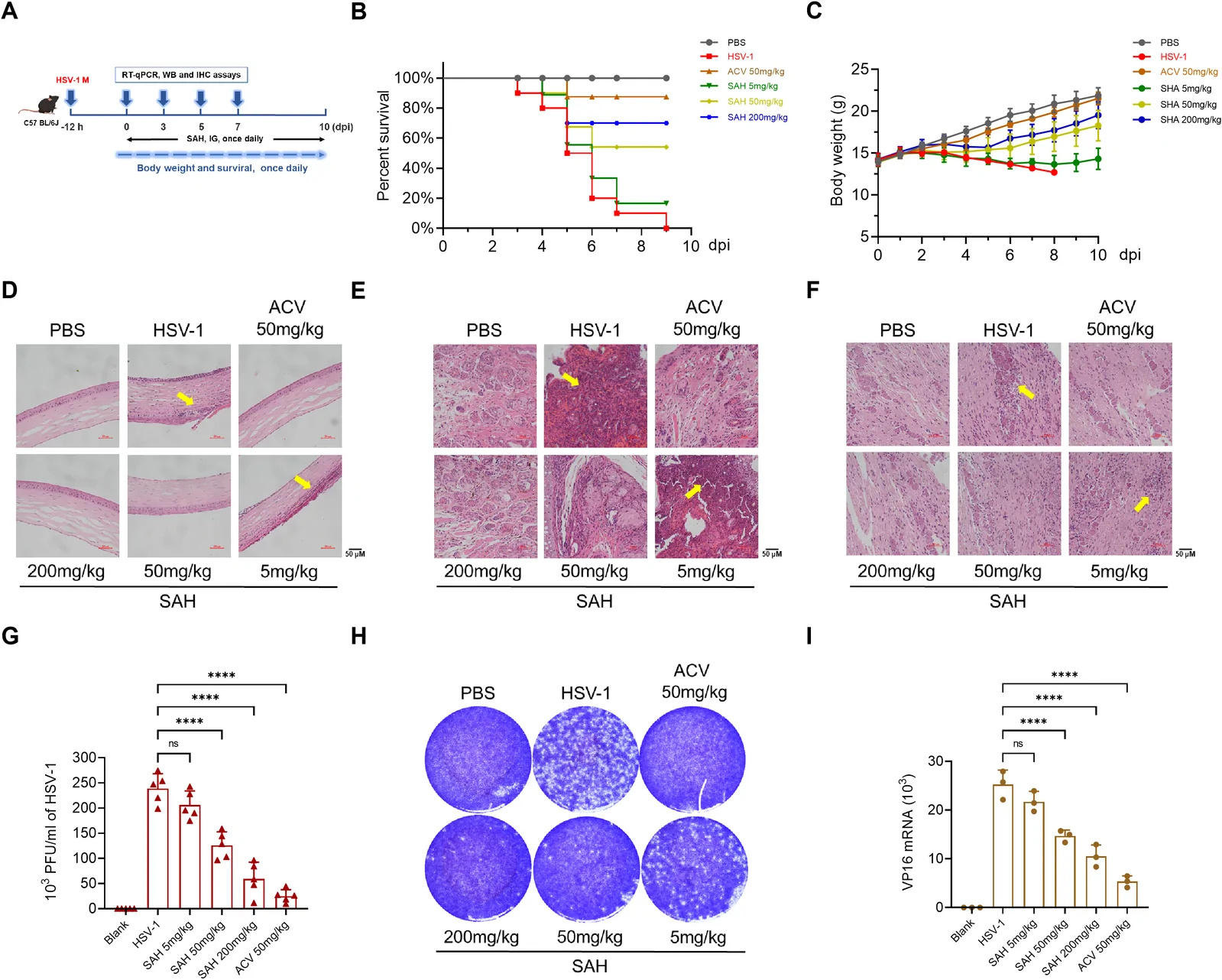
Intragastrical SAH effectively alleviated symptoms and improved survival rate of mice in HSV-1 infection. **(A)** Flowchart for treating HSV-1 M infected C57BL/6J mice with SAH by IG, with acyclovir serving as the positive control drug. **(B, C)** Survival curves and body weight changes of HSV-1–infected mice treated with SAH (5, 50 or 200 mg/kg), n = 10 (5 males, 5 females). **(D-F)** H&E staining of the cornea, periocular skin, and trigeminal nerves at day 5, scale bars: 50 µm. **(G, H)** The infected mice eyeballs of different groups were repeatedly rinsed with 100 μl sterile PBS and the virus titer was quantified by plaque assay. **(I)** VP16 mRNA level was used to reflect the inhibitory of SAH on viral load in the trigeminal nerve of HSV-1 infected mice. Data are shown as means ± SEM from three or more independent experiments. Statistical significance was assessed by one-way ANOVA. Survival analyses were performed using the Kaplan–Meier method with statistical comparisons performed using the two-sided log-rank test. *ns*, not significant; ****P < 0.0001.

### Targeting GBP7 confers broad-spectrum antiviral activity

The mechanism study suggests that GBP7 acts on the protein translation stage of virus replication cycle, indicating that its effect may not be limited to HSV-1. Accordingly, we incorporated Vesicular Stomatitis Virus (VSV), Influenza A virus (IAV), Dengue Virus (DENV), and Zika virus (ZIKA) to conduct further investigations. In A549 cells individually infected with VSV, IAV, DENV, or ZIKV, the transcription of the GBP7 gene was significantly upregulated, and the GBP7 mRNA levels increased with time-dependent upward trend (Fig 8A). We also compared the effects of GBP7 gene presence versus absence on the infection processes of several viruses. The results demonstrated that the viral mRNA levels of VSV-P, IAV-NP, ZIKV-E, and DENV-E were remarkably lower in GBP7-KO cells (Fig 8B), indicating that GBP7 deficiency exerts a broad-spectrum inhibitory effect on viral infection. To evaluate the therapeutic potential of pharmacological GBP7 inhibition, SAH was employed to assess its antiviral activity against VSV, IAV, ZIKV, and DENV infections. It can be seen that the transcription levels of the aforementioned viral proteins exhibited a significant downward trend following SAH intervention (Fig 8C), highlighting the considerable potential of GBP7 as a broad-spectrum antiviral drug target.

**Fig 8:**
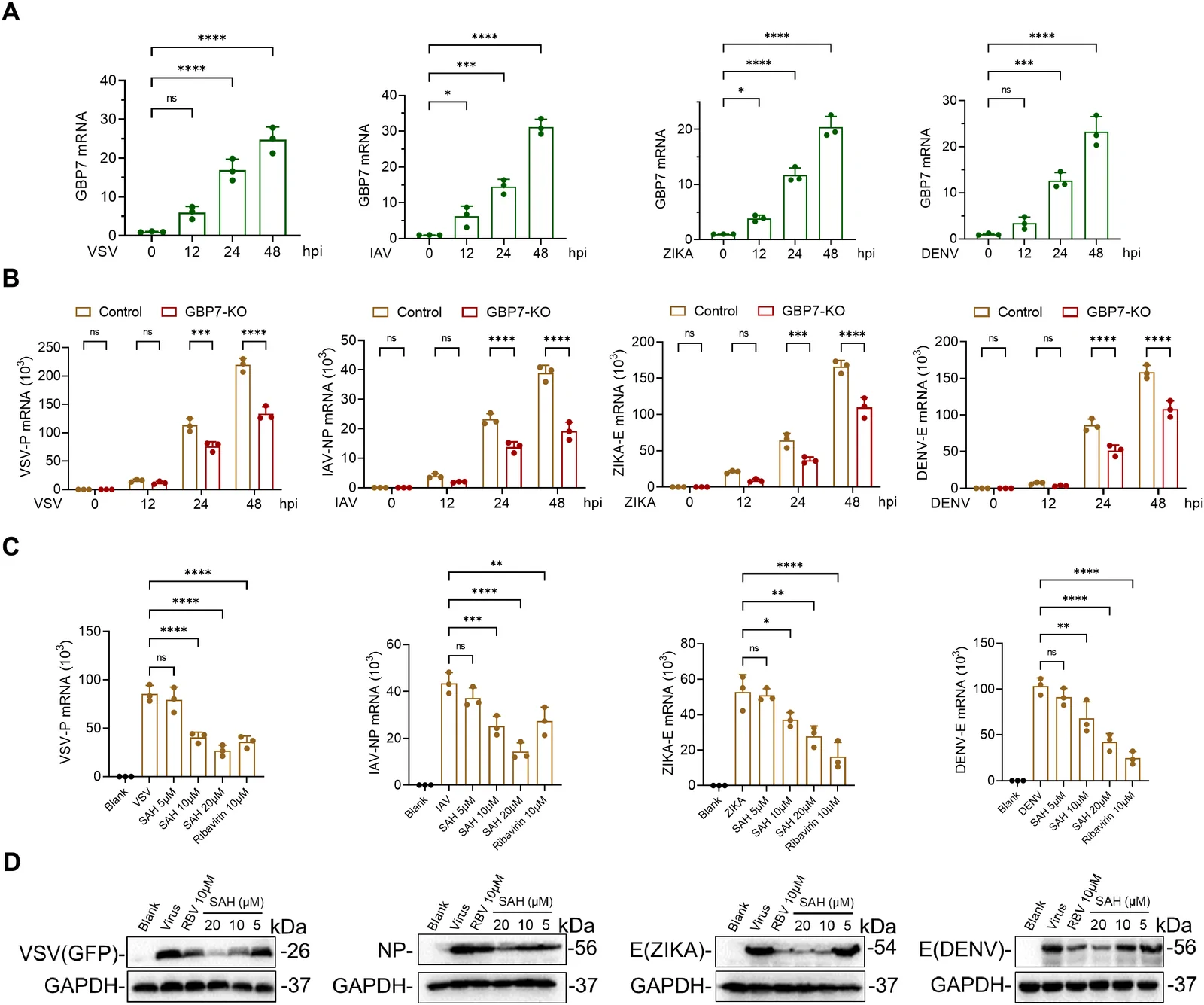
SAH was validated for its broad-spectrum antiviral efficacy as an inhibitor targeting GBP7. **(A)** RT-qPCR assay was used to quantify the increasing levels of GBP7 mRNA in A549 cells infected with VSV, IAV, ZIKA, and DENV viruses. **(B)** RT–qPCR analysis of viral replication in GBP7-KO versus WT cells at 12, 24, and 48 h post-infection with VSV, IAV, ZIKV, or DENV. **(C, D)** RT-qPCR and western blotting analysis of SAH against VSV, IAV, ZIKA, and DENV viral infections, ribavirin was used as the broad-spectrum antiviral drug control. All data are shown as means ± SEM. Three independent experiments were performed. Statistical significance was assessed by one-way ANOVA in **A**, **C** and two-way ANOVA in **B**. *ns*, not significant; *P < 0.05; **P < 0.01; ***P < 0.001; ****P < 0.0001.

## Discussion

As an interferon-stimulated gene (ISG), GBP7 exhibits robust upregulation in tissues and cells during viral infection; however, unlike other GBP family members that exert antiviral effects, GBP7 plays an important role in promoting HSV-1 infection. In terms of the mechanism, GBP7 is likely to serve as a “menace” concealed in the host antiviral immune pathway to promote the replication and infection of the invading virus. Under such circumstances, the host also lacks an effective approach for recognizing and degrading the GBP7 protein. We also focused on the potential impacts of GBP7 gene deletion on physiological functions. Notably, neither the GBP7-KO cells nor the mice exhibited obvious issues with proliferation and growth, associated with the typically low expression level of endogenous GBP7 in tissue cells which means that GBP7 is less likely to disturb the normal physiological activities of cells. We separately examined the effects of overexpression and knockout on the cells or tissues infected by HSV-1 virus. The results consistently indicated that the accumulation of GBP7 is conducive to viral replication and infection. As an interferon-stimulated gene, it has an unusual function in facilitating viral infection. This mechanistic distinction has important therapeutic implications: targeting GBP7 may selectively disrupt viral replication without broadly suppressing interferon-mediated host defense.

Another major finding of our study was that eEF1A1 is a partner that interacts with GBP7. Through mass spectrometric analysis of the proteins that interact with GBP7, we focused on another host protein with a high PSM value, eEF1A1, which has been reported to promote viral infection [39,46,47]. From the perspective of viral infection, particularly in promoting viral infections, we eventually selected eEF1A1 as the research subject and promptly verified a protein-protein interaction between eEF1A1 and GBP7. Additionally, extensive colocalization of the two proteins was observed in the cells. We also found that viral infection not only increased the protein level of GBP7 but also enhanced the expression of eEF1A1, suggesting a possible correlation between the two proteins in virus infections. We further investigated the mode of binding and interaction between eEF1A1 and GBP7, aiming to clarify the specific mechanisms that facilitate viral infection. Protein structure analysis of the eEF1A1 and GBP7 complexes will undoubtedly provide the greatest assistance to our research. Therefore, we attempt to analyze some key structural information of the eEF1A1 and GBP7 complex from other perspectives. The molecular docking results indicated that the interaction was likely associated with the GED domain of the GBP7 protein. When GTP binds to GBPs, GBPs assemble into dimers through contact between the LG domain and the GED domain, thereby stimulating their enzymatic activity for the hydrolysis of GTP [48,49]. Therefore, we tested the binding ability of the three truncated proteins and found that the MD domain had a stronger binding ability to eEF1A1 than the other two domains, which did not affect the formation of the dimer. We also mutated the predicted binding sites to further clarify the key binding sites of GBP7 with eEF1A1, such as R343 and E362, and to elucidate the interaction mode between eEF1A1 and GBP7, providing some structural information on the eEF1A1-GBP7 complex.

While the eEF1A1 gene is deficient in cells, not only is viral infection directly inhibited but also the ability of GBP7 to promote viral infection vanishes accordingly. These data indicate that there is a “synergy” between eEF1A1 and GBP7, in which eEF1A1, regulated by GBP7, assumes the role of directly promoting infection. eEF1A1 has been proposed as a target for antiviral research due to its role in translation elongation [50–52]. However, the translation elongation process is indispensable for intracellular protein production. Interference with the host protein eEF1A1 does indeed produce cytotoxicity, but its toxicity is dose-dependent and tissue-specific [53]. The GTPase activity is essential for eEF1A1 in decoding mRNA [54], suggesting us that the eEF1A1-GBP7 axis can promote viral infection by facilitating the translation of viral proteins through the GTPase of GBP7. A series of experiments was conducted to investigate the role of the eEF1A1-GBP7 complex in protein translation. The results indicate that host factors, specifically GBP7, positively regulate viral infection by binding to eEF1A1 and utilizing its GTPase activity to promote eEF1A1-mediated translation elongation. Although plitidepsin can selectively inhibit SARS-CoV-2 virus translation and preserve cell viability at low concentrations [20], its clinical efficacy has not been ultimately confirmed. Plitidepsin is also associated with dose limiting toxicity (DLT) in the study of relapsed/refractory multiple myeloma [55]. Inhibiting GBP7, an interferon-stimulated gene “concealing” within the immune signaling pathway, may present a more feasible strategy to combat viral infections. We identified SAH, an inhibitor targeting GBP7, which exhibited excellent antiviral efficacy in HSV-1 infection. SAH reduces the translation of viral proteins by interfering with the expression and GTPase activity of GBP7, eventually fulfilling the function of inhibiting infection, which shows considerable promise as a broad-spectrum antiviral target. In infections with VSV, IAV, ZIKV or DENV viruses, elevated expression of the GBP7 protein was consistently observed. Concomitantly, infection by these viruses was suppressed to varying degrees in GBP7-KO cell lines. By targeting GBP7, SAH also exhibits broad-spectrum antiviral activity. However, several limitations necessitate further investigation. First, it is imperative to validate whether GBP7 can be effectively activated across diverse tissue types. This validation is crucial for determining the broader applicability of this mechanism to a wider spectrum of viral infections, which has significant implications for the development of broad-spectrum antiviral drugs targeting GBP7. While SAH has been identified as a potential targeted inhibitor of GBP7, additional direct experimental evidence is required to validate this targeting relationship. Furthermore, future studies elucidating the full-length structure of the GBP7 protein in a complex with eEF1A1 would provide valuable molecular insights into the interaction between GBP7 and eEF1A1.

In conclusion, the evidence presented in this manuscript substantiates the positive regulatory effect of the interferon-stimulated gene GBP7 on the infection processes of HSV-1, which is depended upon the interaction with eEF1A1 to facilitate the translation process of proteins in HSV-1infection. GBP7 holds promise as a safe and effective host target for disrupting the life cycle of HSV-1, thereby exhibiting potential utility in the development of host-targeted antiviral agents. Additionally, its demonstrated broad-spectrum antiviral activity warrants further investigation.

## Materials and methods

### Cells and culture

The Vero (African green monkey kidney cell), HEK-293T cell lines were obtained from ATCC (Manassas, VA, USA) and cultured in Dulbecco’s modified Eagle’s medium (GIBCO, Cat# C11995500BT) containing 10% fetal bovine serum (FBS, ExCell, Cat# FSP500) with 1% penicillin/streptomycin (P/S, GIBCO, Cat# 15140122) at 37°C and 5% CO_2_. A549 human non-small cell lung cancer cells (ATCC, Manassas, VA, USA) were cultured in RPMI-1640 medium (GIBCO, Cat# C11875500BT) supplemented with 10% FBS and 1%P/S at 37°C and 5% CO_2_.

GBP7-knockout A549 cells were generated via lentiviral transduction. These cells were transduced with lentivirus expressing Cas9 and specific small guide RNAs (sgRNAs) (S3 Table) for 48 h, then centrifuged for 2 h at 1000/rcf and selected with 5 µg/ml puromycin (Beyotime, Cat# ST551). The knockout was verified at the protein level and monoclonal cell lines were screened out.

### Bacterial strains

*E. coli* Stbl3 Chemically Competent Cells (TSINGKE, Cat# TSC-C06) and BL21 (DE3) Chemically Competent Cells (TSINGKE, Cat# TSC- E01) were used for molecular cloning and cultured in Luria-Bertani (LB) medium at 37°C.

### Ethics statement

All of the experimental procedures involving animals were conducted in accordance with the ethical guidelines established by the Institutional Animal Care and Use Committee (IACUC) of Southern Medical University, reviewed and approved by this ethics committee of experimental animals (Approval Number: SMUL202405024). And the experiments complied with the World Medical Association (WMA) Statement on animal use in biomedical research.

### Animal experiments

The wild-type (WT) mice used in this study were 4-week-old female and male mice with a C57BL/6J genetic background purchased from the Animal Center of Southern Medical University (China). All mice were reared in the specific pathogen-free animal facility of the Experimental Animal Center of Southern Medical University. The external environment of the mice consisted of a 12-h light/dark cycle, with an ambient temperature ranging from 20°C to 26°C and a relative humidity ranging from 40% to 70%.

Conventional GBP7 knockout mice with a C57BL/6J genetic background were obtained from Cyagen (Cyagen Biotechnology Co., Ltd., China) and genotyped by PCR to select homozygous animals. The primers used for genotyping were as follows:

Primers1 F1: GAACTTATTTTGGAGGACAAGGCT
Primers1 R1: ATAAGCAGAAGAAATTGCCAGGTC
Primers2 F1: GAACTTATTTTGGAGGACAAGGCT
Primers2 R1: TTTTCCACATCTCCTAAGCCCTC

### Virus and viral infections

The HSV-1 F and HSV-1/GFP strain was employed for cellular infection. The HSV-1 M strain was used for mouse infection. After the induction of anesthesia via isoflurane, the corneas of both eyes of the mice were topically inoculated with a suspension containing the HSV-1 M virus. The weight changes of the mice were recorded by performing daily weight measurements. Vesicular Stomatitis Virus (VSV), Influenza A virus (A/WSN/1933), Dengue Virus (DENV-2) and Zika virus (ZIKA) were utilized for infecting cells.

### Biosafety statement

All experiments involving infectious agents were performed in a certified Biosafety Level 2 (BSL-2) laboratory at Southern Medical University, in compliance with the guidelines outlined in the CDC/NIH Biosafety in Microbiological and Biomedical Laboratories. The four viruses used in this study, including herpes simplex virus type 1 (HSV-1), Zika virus (ZIKV), dengue virus (DENV), and influenza A virus (IAV) are classified as Risk Group 2 (RG2) agents by the NIH and the World Health Organization (WHO), and all procedures were performed in compliance with biosafety level 2 (BSL-2) laboratory requirements.

### Plaque assays

The supernatants from cells infected with HSV-1 were diluted and added to Vero cells. After incubation for 1 h at 37°C, the supernatants were discarded and replaced with the corresponding maintenance culture medium: DMEM containing 2% FBS and 1% methylcellulose for Vero cells. The mixture was incubated for 48–72 h at 37°C and 5% CO_2_. The maintenance medium was then discarded, and 4% paraformaldehyde (LEAGENE, Cat# DF0135) was added to fix the cells for 10 min. Following staining with crystal violet, the viral plaques were imaged via an enzyme-linked immunospot analyzer.

### Immunofluorescence (IF)

The tissues were fixed in 4% paraformaldehyde for 15 min, and the mouse paraffin-embedded tissue sections were dewaxed, subjected to antigen retrieval, and then permeabilized in 0.1% Triton X-100 (Cat# T109026) for 10 min. Following washing with PBS, the sections were blocked with 3% FBS at room temperature for 1 h. Primary antibodies against HSV-1 VP16 (Abcam, Cat# ab110226), eEF1A1 (Santa Cruz Biotechnology, Cat# sc-377439), or GBP7 (Abcam, Cat# ab104293) were used for the fluorescence staining of proteins, while the nuclei were subjected to DAPI dilution. Goat anti-rabbit IgG (Alexa Fluor 488) (CST, Cat# 4412S) or goat anti-mouse IgG (Alexa Fluor 594) (CST, Cat# 8890S) served as the secondary antibodies. Fluorescence images were captured via an Axio Observer microscope (Zeiss).

### Hematoxylin and eosin (H&E) staining

Eye, trigeminal nerve, and periocular skin were collected from infected mice and fixed in 4% paraformaldehyde before being embedded in paraffin. Paraffin-embedded tissue sections were deparaffinized, rehydrated, and subjected to heat-induced antigen repair prior to being stained with primary antibodies against HSV-1 VP16 to observe viral infection in the tissues. After incubation at 4°C overnight and incubation with secondary antibodies at RT for 1 h, the sections were washed and visualized via DAB (ZSGB-BIO) with hematoxylin (ZSGB-BIO) counterstaining.

Sections were stained with H&E to compare the degree of inflammation at the virus-infected sites and the degree of histopathological changes. All brightfield images were acquired using a Nikon ECLIPSE 90i microscope.

### Plasmid constructs and transfection

The GBP7 (NM_207398.2) ORF expression clone (C-flag tagged) and the vector plasmid were purchased from GeneCopoeia, USA. The pCMV-eEF1A1 human plasmid was obtained from MIAO LING BIO (China). The R48A, K51A and other mutants were constructed using the CloneExpress II One-Step Cloning Kit (Vazyme, Cat# C112-02) with the GBP7 plasmid as a template. Other truncated and mutant plasmids were synthesized by Tsingke Biotechnology Co., Ltd. (China) based on the GBP7 template.

Short interfering RNA (siRNA) was transfected into cells using Lipofectamine 2000 (Thermo, Cat# 11668019) in Opti-MEM reduced-serum medium (GIBCO, Cat# 31985070), and the interference efficiency was assessed 24 h post-transfection. siRNAs targeting eEF1A1 were purchased from GenePharma Co., Ltd. (China), respectively. The sequences of the siRNAs used are listed in S4 Table.

### Immunoprecipitation coupled with mass spectrometry (IP-MS)

HEK-293T cells were transfected with GBP7-Flag while being simultaneously infected with HSV-1. After incubation for 24 h, the cells were lysed with RIPA lysis buffer (Beyotime, Cat# P0013) and subsequently centrifuged at 12,000 rpm and 4°C for 15 min. The supernatant was incubated overnight at 4°C with Proteo G Resin (GenScript, Cat# L00209-50), which had been preincubated with either GBP7-Flag or IgG antibodies (CST, Cat# 2729S). The beads were washed with IP buffer and sent to Wayen Biotech (Shanghai, China) for proteomic mass spectrometry (MS) analysis of protein-protein interactions. HPLC-MS data acquisition was performed on an EASY-nLC 1000 system (Thermo Scientific, USA) and an Orbitrap Fusion Lumos instrument (Thermo Scientific, USA). Finally, the acquired data were analyzed via Proteome Discoverer 2.4.

### AlphaFold2 prediction and docking analysis

The structures of human eEF1A1 and human GBP7 were constructed using AlphaFold2(v2.2.0, Jumper et al, Nature 2021 [56]). The molecular docking of the GBP7-eEF1A1 complex was subsequently performed via the HDOCK program (v1.0, Yan et al, 2017 [57]), and the structure with the best docking score was selected as the reference for subsequent interaction analyses. The docking score was calculated based on the iterative scoring functions of ITScorePP or ITScorePR (Confidence score higher than 0.7: highly likely to bind; Confidence score between 0.5 and 0.7: likely to bind; Confidence score lower than 0.5: unlikely to bind).The calculation method is presented as follows:


$$\begin{array}{c} Confidence\_score=1.0/[1.0+e0.02*(Docking\_Score+150)] \end{array}$$

### Protein expression and purification

The proteins GBP7^WT^, GBP7^R48A^, and GBP7^K51A^, each with a Flag tag, were expressed in HEK-293T cells. After cell lysis, the supernatant was collected via centrifugation (12,000 rpm, 4°C), and the proteins were purified via Anti-DYKDDDDK G1 Affinity Resin (GenScript, Cat# L00432). Specifically, bound proteins carrying the DYKDDDDK tag in the cell lysate were selectively captured by a monoclonal antibody against the DYKDDDDK tag conjugated to the resin. Nonspecifically bound impure proteins were eliminated through rigorous washing steps, resulting in high-purity elution of the target protein.

### Drug affinity responsive target stability (DARTS) and Western blot

48 h post-transfection with GBP7^WT^, GBP7^R48^ or GBP7^K51^, HEK-293T cells were lysed using NP-40 lysis buffer (Beyotime) and subsequently incubatedovernight at 4 °C in the presence or absence of SAH (20 μM or 50 μM). Following this treatment,the samples were subjected to digestion with pronase E (5 μg/ml) for 30 min at 37 °C in reactionbuffer containing 50 mM Tris-HCI (pH 8.0), 50 mM NaCl and 10 mM CaCl. The expression of protein was then analyzed by western blotting after the addition of a five-fold loading buffer and boiling.

### Surface plasmon resonance

Surface plasmon resonance (SPR) measurements were performed on a Biacore 1K instrument (Cytiva). GBP7 protein was immobilized onto a CM5 sensor chip via amine coupling according to the manufacturer’s protocol. Serially diluted compounds in running buffer (1 × PBS-P ⁺ , pH 7.4, 5% DMSO) were injected over the GBP7-immobilized and reference flow cells at 30 µL/min for 60 s, followed by 60 s dissociation. The chip surface was regenerated with 10 mM glycine-HCl (pH 2.0). Sensorgrams were processed by double referencing and fitted to a 1:1 Langmuir binding model using Biacore Insight Evaluation software to calculate the equilibrium dissociation constant (KD).

### GTPase activity assay

The purified protein was quantified, and the GTPase activity was determined at the same concentration via the ATPase/GTPase Activity Assay Kit (Sigma-Aldrich, Cat# MAK113). The GBP7 protein in a concentration gradient catalyzes the hydrolysis of GTP to yield GDP and free phosphate. The malachite green reagent forms a stable dark green color when it reacts with the free phosphate liberated by the enzymes. This colorimetric product is measured at 620 nm and is proportional to the enzyme activity present.

### Protein translation assay

The T7 promoter-bearing circular DNA was incorporated into the TnT Quick Coupled Transcription/Translation System (Promega, Cat# L1170) for in vitro transcription and translation. The TnT Quick reaction system, comprising RNA polymerase, nucleotides, salts, recombinant RNasin ribonuclease inhibitor, and reticulocyte lysate mixture, effectively interacts with the template DNA at 30°C to synthesize protein. Western blotting analysis was performed to compare the effects of eEF1A1 and GBP7 on the translation process.

### Western blotting analysis

Cells or mouse tissue homogenates were lysed in RIPA lysis buffer. The whole lysates were boiled at 105°C for 15 min, and 5 × loading buffer was added. Proteins were subsequently separated via sodium dodecyl sulfate-polyacrylamide gel electrophoresis (SDS-PAGE) and transferred onto a PVDF membrane (ROCHE, Cat# 03010040001). After blocking with 5% skim milk, the protein bands were incubated with specific primary antibodies, followed by incubation with the corresponding horseradish peroxidase-conjugated secondary antibodies. The protein bands were detected via an enhanced chemiluminescence (ECL) kit (NCM, Cat# P10300) with a chemiluminescence analyzer. The source, catalogue number and lot for commercial antibodies are shown in S5 Table.

### Quantitative reverse-transcription PCR (RT-qPCR)

The RNA was extracted via a total RNA isolation kit (Vazyme, Cat# RC202–01) and quantified. Equivalent amounts of mRNA were reverse-transcribed via the HiScript III RT SuperMix for qPCR Kit (Vazyme, Cat# R323-01). RT-qPCR amplification was performed via the Roche LightCycler 480 and Taq Pro Universal SYBR qPCR Master Mix assays (Vazyme, Cat# Q712-02). The mRNA expression values were normalized to the level of GAPDH, and the data were analyzed via the Livak method (2^-ΔΔCt^). RT-qPCR cycling conditions: 95 °C for 30 s, followed by 40 cycles of 55°C for 30 s and 75°C for 30 s, with melting curve analysis.The custom designed primer sequences are shown in S6 Table.

### Statistical analysis

All the data were analyzed via GraphPad Prism 9.0. All statistical details of the experiments, including the exact values of n and the corresponding representation, are provided in the figure legends and presented as means ± SEMs. Two-tailed unpaired or paired Student’s t-tests were used for comparisons between two groups. For multiple-group comparisons, one-way ANOVA with Tukey’s or Dunnett’s multiple-comparisons tests, or two-way ANOVA with Tukey’s multiple-comparisons test were used. Categorical variables were analyzed using chi-square tests. Correlations were assessed using Pearson’s correlation (two-tailed).

Survival analyses were performed using the Kaplan–Meier method, and differences between groups were compared using the two-sided log-rank (Mantel-Cox) test. For other continuous variables, normality of distribution was assessed using the Shapiro-Wilk test, and homogeneity of variances was evaluated using Levene’s test. For data meeting normality and homogeneity assumptions, comparisons between two groups were conducted using the two-tailed Student’s t-test; for comparisons among multiple groups, one-way ANOVA followed by Tukey’s post-hoc test was applied.

## Supporting information

S1 Fig HSV-1 infection activates GBP7 protein expression in different target cells in time- and titer-dependent manners. (A) Expression of GBP7 protein at different time points after HSV-1 infection of Vero and Hela cells. (B) Under different MOI of HSV-1 infection conditions, the expression of GBP7 protein in A549 cells. Protein was measured by using western blotting.
(TIF)

S2 Fig GBP7 overexpression upregulates the expression of HSV-1 VP16 protein. Immunofluorescence images indicated that more viral VP16 protein accumulated in GBP7-overexpressed A549 cells at 24 and 48 hours after HSV-1 infection. Scale bars, 50 μm.
(TIF)

S3 Fig Construction of GBP7 gene knockout cell line and detection of cell replication function. (A) Three sgRNAs edited by CRISPR-Cas9 for silencing GBP7-gene expression. (B) CCK8 detection of WT and GBP7-KO A549 cells (sgRNA-2) at different time points.
(TIF)

S4 Fig The replication and infection of HSV-1 were inhibited in A549 cells with GBP7 gene knockout. Representative immunofluorescence images of HSV-1 VP16 in GBP7-KO and control cells at the indicated time points post-infection. Scale bars, 50 μm.
(TIF)

S5 Fig GBP7-KO has no negative effect on the growth and development of mice. (A) Strategy of CRISPR-Cas technology to target and knockout the GBP7 gene in C57BL/6J mice. (B) Genotyping by PCR amplification of toe tissue DNA, followed by gel electrophoresis. (C) Verification of gene knockout effect of mice cornea, trigeminal nerve and periocular skin tissue cells via western blotting. (D) Morphology of 4-week-old GBP7-/- and WT mice. (E) H&E staining of the heart, liver, spleen, lung and kidney tissues to support that GBP7 knockout did not affect organ development in mice. Scale bars, 200 μm.
(TIF)

S6 Fig GBP7 knockout reduces HSV-1 VP16 protein in the trigeminal nerve. VP16 protein in trigeminal nerve of GBP7-/- and WT mice infected with HSV-1 was detected by IF staining at 5 and 7 days post-infection. Scale bars, 200 μm.
(TIF)

S7 Fig Identification of the binding sites between GBP7 and eEF1A1. (A) KEGG pathway enrichment analysis of GBP7-specific interactors implicating protein synthesis. (B) Protein-Protein Interaction (PPI) network of the interacting proteins of GBP7. (C, D) Co-IP validation of the GBP7–eEF1A1 interaction in 293T cells co-transfected with GBP7-Flag and eEF1A1-His. (E, F) The three-dimensional structure models of GBP7 and eEF1A1 for protein-protein docking are in AlphaFold2. (G, H) The binding site information predicted for the docking between GBP7 and eEF1A1, as well as the construction of single-point mutant proteins at the corresponding sites. (I) CO-IP analysis of different single-point mutations of GBP7 protein binding to eEF1A1.
(TIF)

S8 Fig Identification of GBP7-eEF1A1 axis promoting HSV-1 infection. (A) Western blotting analysis of gD protein levels in cells transduced with GBP7 and eEF1A1 individually or co-transduced at the points of 12, 24 and 48 h after HSV-1 infection. (B) Three small interfering RNAs were transduced into cells to interfere with the expression of eEF1A1 protein. (C) Western blotting results showed that the inhibition of eEF1A1 expression would lead to the weakening or even disappearance of the HSV-1 infection-promoted function of GBP7. Quantification was analyzed by Image J. All data are shown as means ± SEM. Statistical significance was assessed by two-way ANOVA. ns, not significant; *P < 0.05; ***P < 0.001; ****P < 0.0001.
(TIF)

S9 Fig Effect of GBP7 on the contents of GTP, GDP and GMP in HSV-1 infected cells. (A to C) ELISA quantitative analysis was conducted to measure the concentration of GTP, GDP, and GMP in cells transduced with GBP7WT, GBP7R48A and GBP7K51A mutants during HSV-1 infection. (D to F) ELISA was used to detect the contents of GTP, GDP, and GMP in GBP7-KO cells at different time points after HSV-1 infection with the comparison of the control. All data are shown as means ± SEM. Three independent experiments were performed. Statistical significance were assessed by one-way ANOVA in A to C and two-way ANOVA in D to F. ns, not significant; *P < 0.05; **P < 0.01; ***P < 0.001; ****P < 0.0001.
(TIF)

S10 Fig Virtual screening and structural information of SAH. (A) The linear correlation coefficient between the scoring value of molecular docking results and molecular weight was used to evaluate the reliability of scoring function and screening method. (B) Fingerprint analysis of the protein ligand interaction (Protein Ligand Interaction Fingerprints, PLIF). (C, D) The compound SAH was identified by nuclear magnetic resonance (HNMR) spectrum and high-performance liquid chromatography coupled with mass spectrometry (LC-MS).
(TIF)

S11 Fig DARTS assays to detect the interaction between SAH and GBP7 mutant protein. Western blotting detection of affinity between SAH and GBP7R48, GBP7K51 proteins through Drug Affinity Responsive Target Stability (DARTS) assay.
(TIF)

S12 Fig CCK8 to detect the toxicity of SAH in cells. Survival rates of A549, Vero, and Hela cells under the gradient concentration of SAH using CCK8 detection.
(TIF)

S13 Fig The effect of compound ACV on GBP7 protein expression determined by western blotting. ACV can not influent the expression of GBP7 protein in a concentration dependent manner in AS49 cells.
(TIF)

S1 Table Peptide-spectrum matches of the interacting proteins with GBP7-Flag from IP-MS (PSM > 1). (DOCX)

S2 Table MMGBSA scoring for the molecular docking of compounds with GBP7. (DOCX)

S3 Table SgRNA primer sequence. (DOCX)

S4 Table SiRNA primer sequence. (DOCX)

S5 Table The source, catalogue number and lot for commercial antibodies. (DOCX)

S6 Table Primers used for quantitative real time PCR analysis. (DOCX)

S1 Raw Images The original uncropped and unadjusted images underlying all blot or gel results reported in Figures and Supporting Figures files. (PDF)

S1 Data Raw data. All raw data required to replicate the results of the study.
(ZIP)

S1 File This schematic illustrates the proposed mechanism by which GBP7 promotes viral protein translation and the inhibitory effect of SAH on this process. Following viral invasion, activation of host immune pathways induces IFN-γ expression, which subsequently upregulates GBP7. Within the host cell, the virus undergoes uncoating, nuclear import, replication, and transcription, culminating in the production of progeny virions. GBP7 interacts with eukaryotic translation elongation factor 1α1 (eEF1A1) and, through its GTPase activity, facilitates viral protein translation. The small-molecule compound SAH specifically targets GBP7 by suppressing both its GTPase activity and protein expression, thereby disrupting the GBP7–eEF1A1 interaction and consequently inhibiting viral protein synthesis, ultimately reducing the yield of progeny virus.
(DOCX)
