# Supplementary material for: GTPase GBP7 promotes HSV-1 infection by interacting with and activating eEF1A1 to accelerate the protein translation process
Source: PLoS Pathog. 2026 Oct 12;22(10):e1014652. doi: 10.1371/journal.ppat.1014652 (PMC13759050; doi:10.1371/journal.ppat.1014652)

Full and uncropped western blot for Fig 1B, Chemiluminescence (ECL)

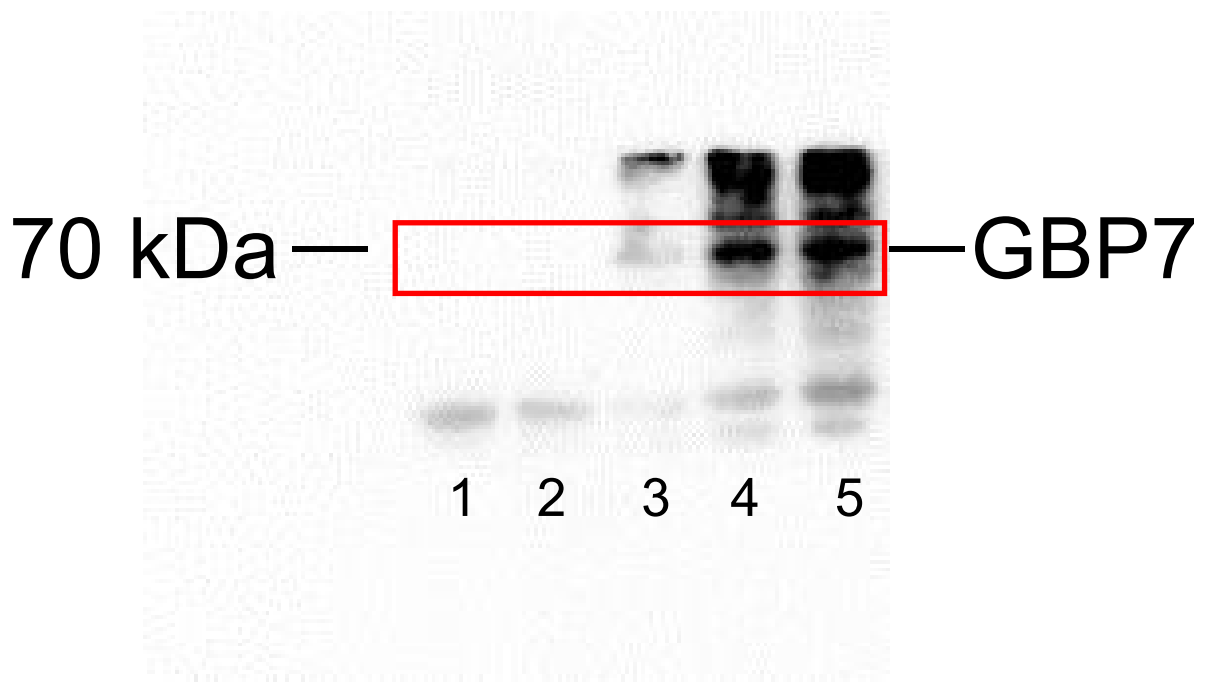


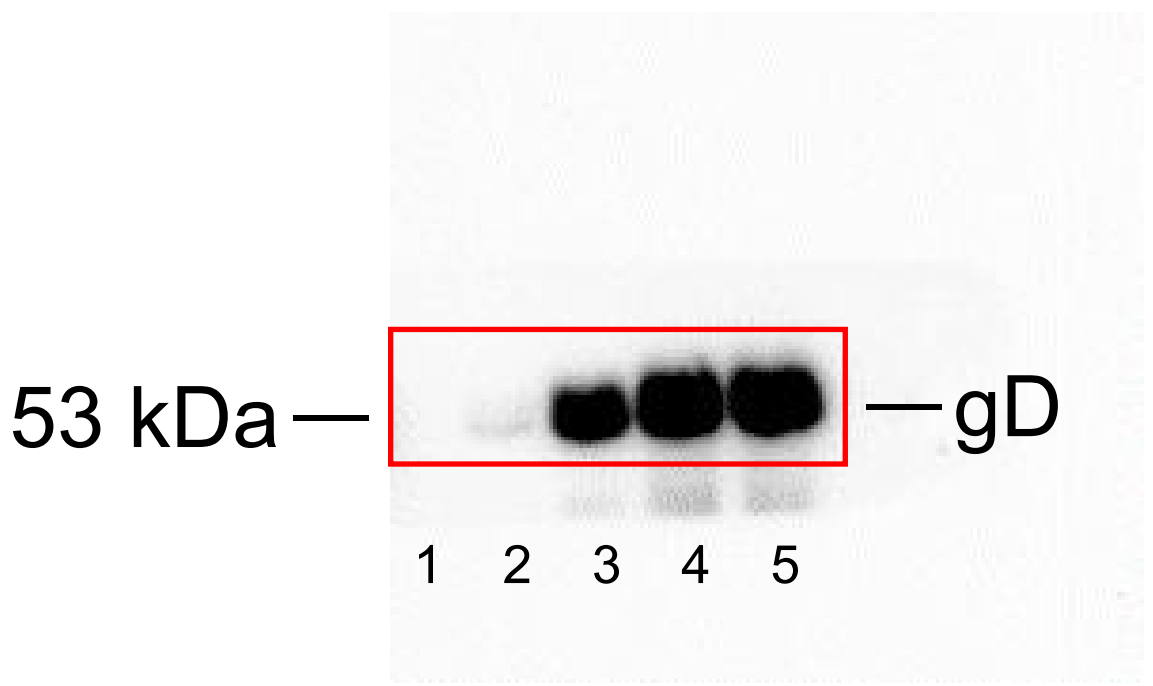


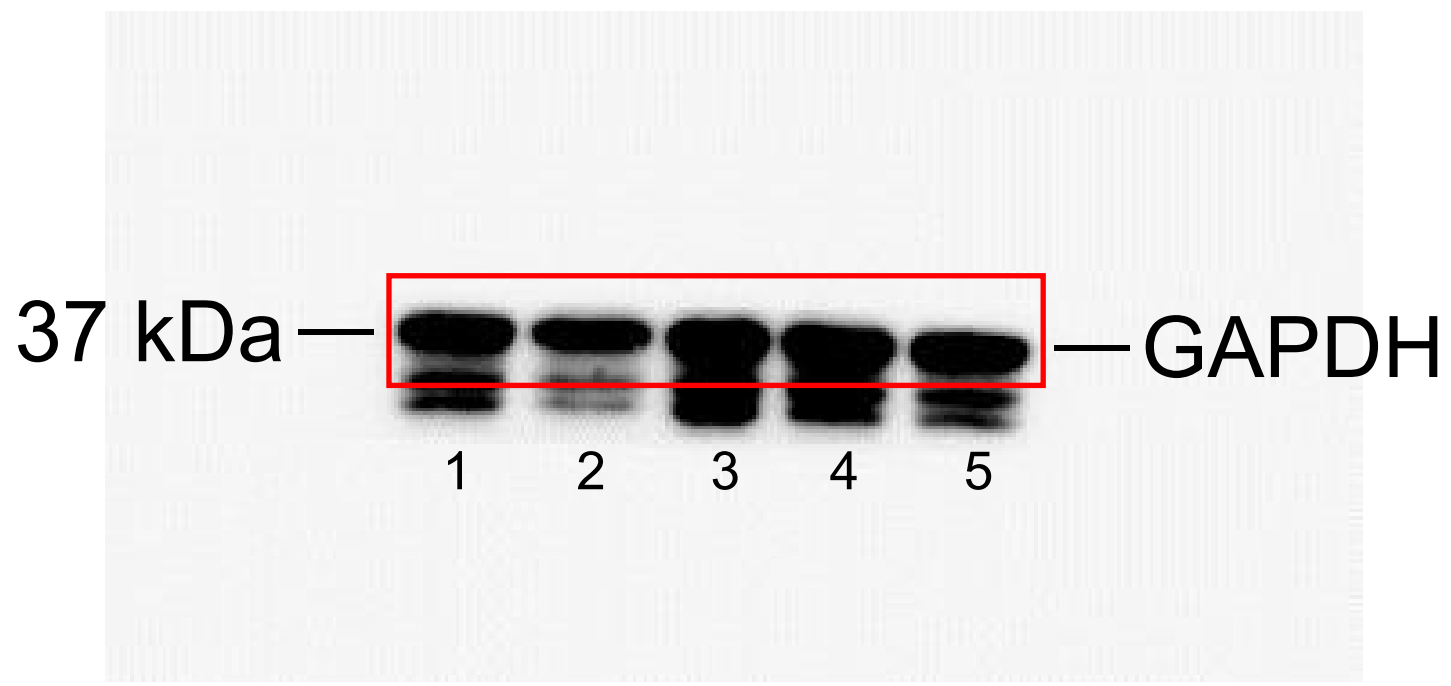

# Full and uncropped western blot for Fig 1E, Chemiluminescence (ECL)

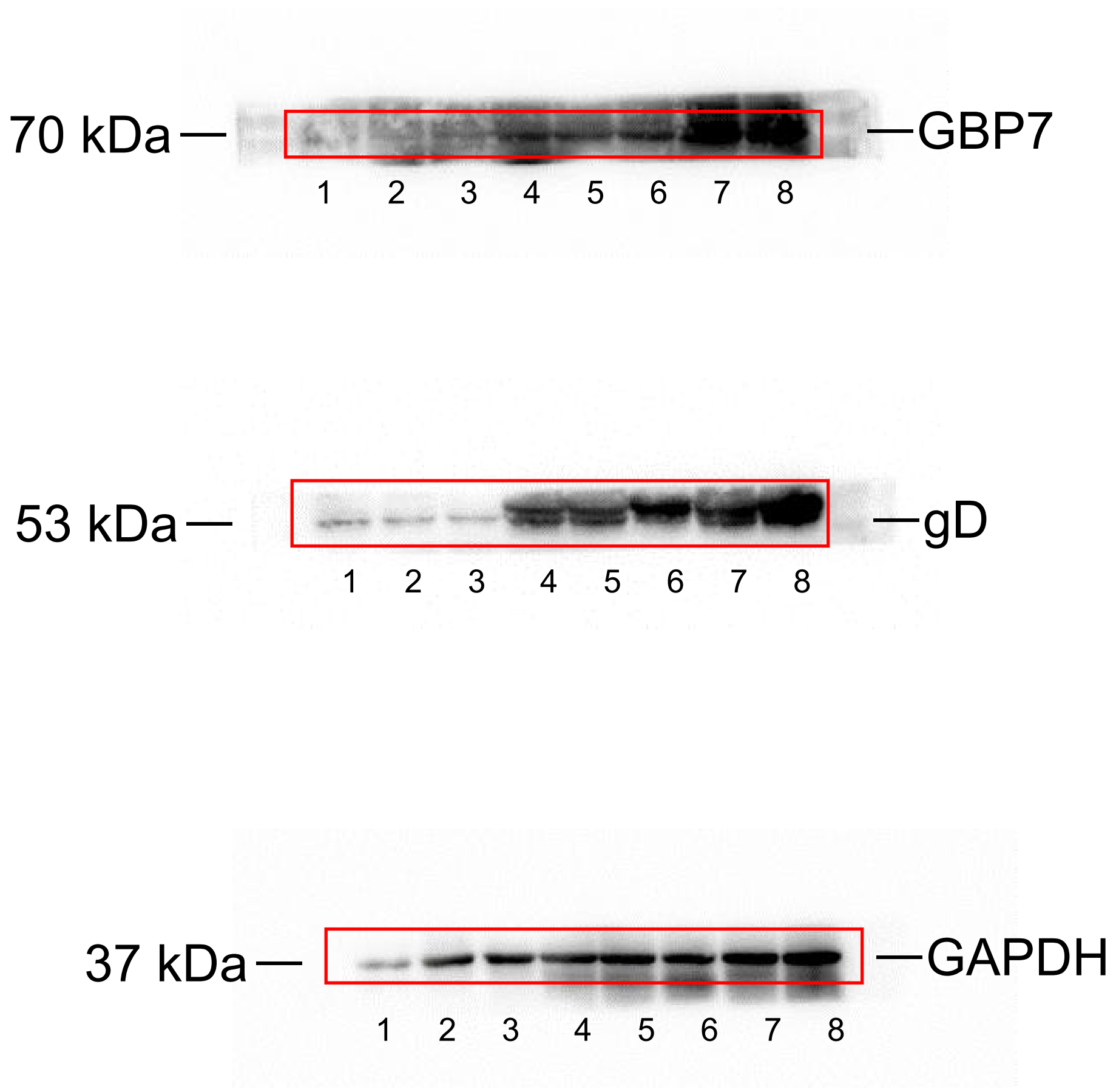

Full and uncropped western blot for Fig 2A, Chemiluminescence (ECL)

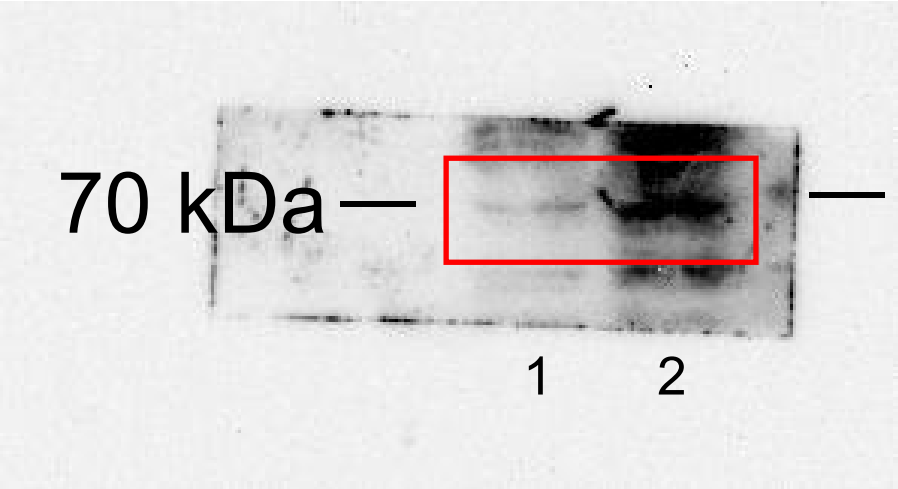


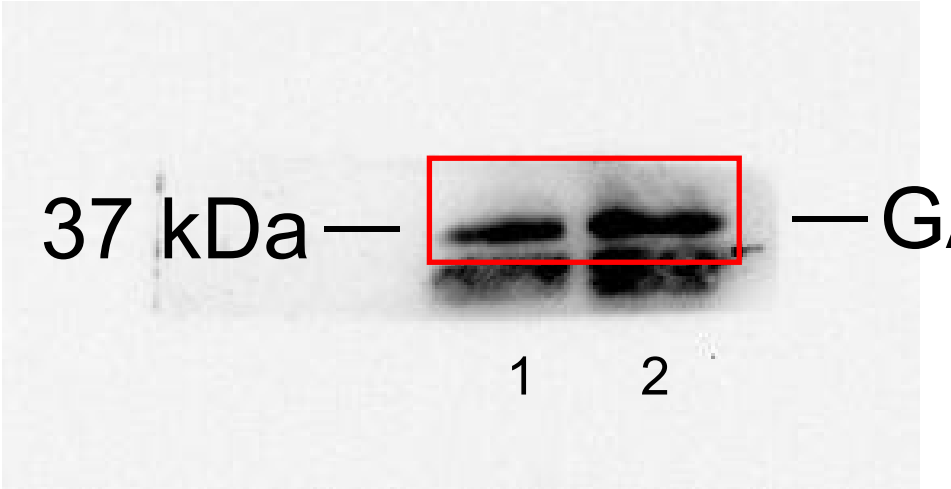

Full and uncropped western blot for Fig 2B, Chemiluminescence (ECL)

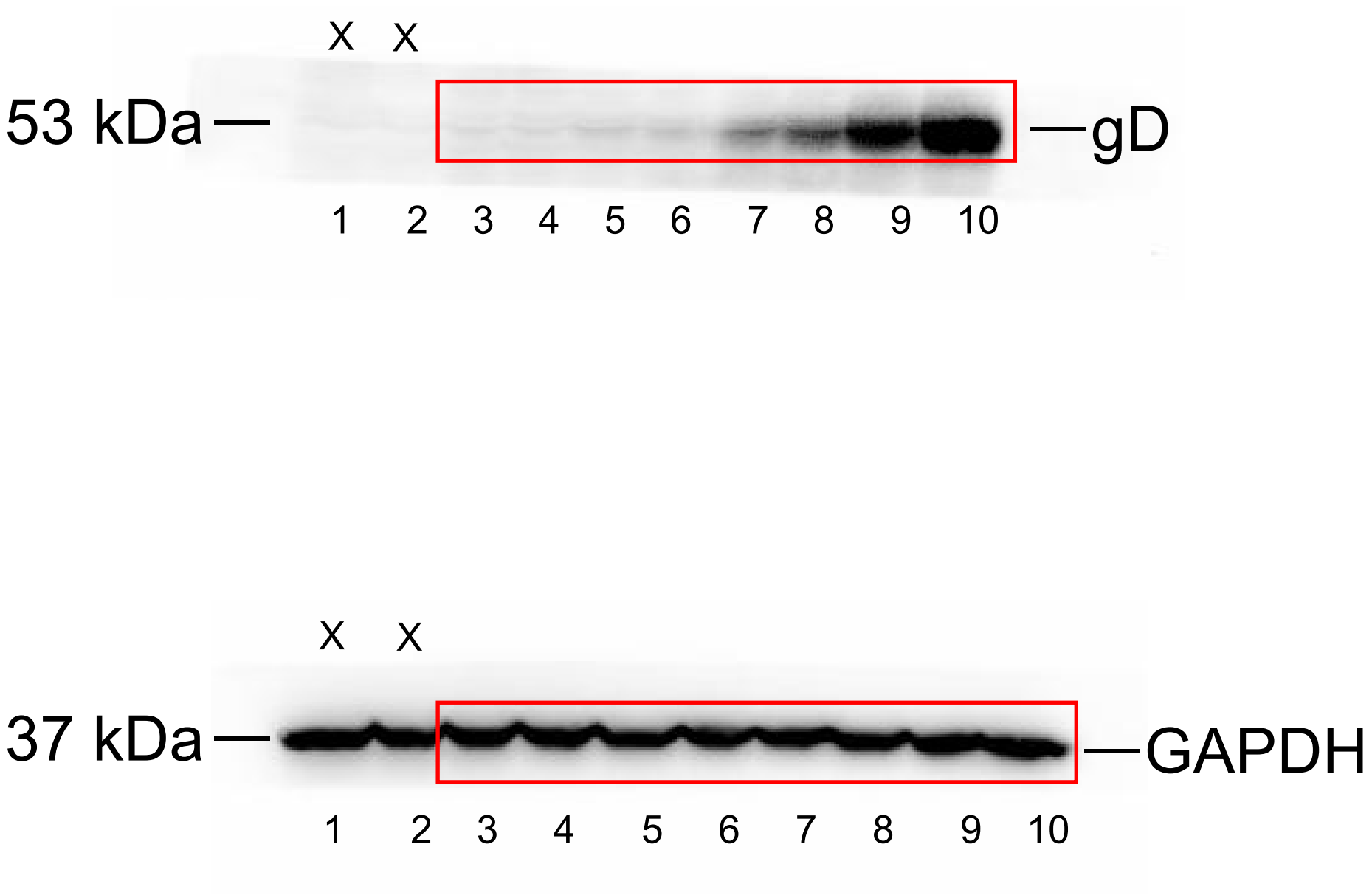


Lanes 1 and 2: wild- type (WT) mock- infected samples

Full and uncropped western blot for Fig 2E, Chemiluminescence (ECL)

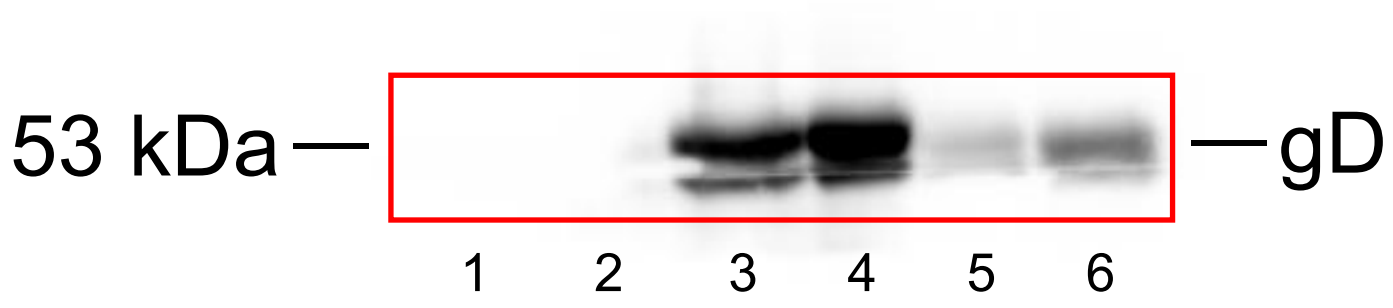


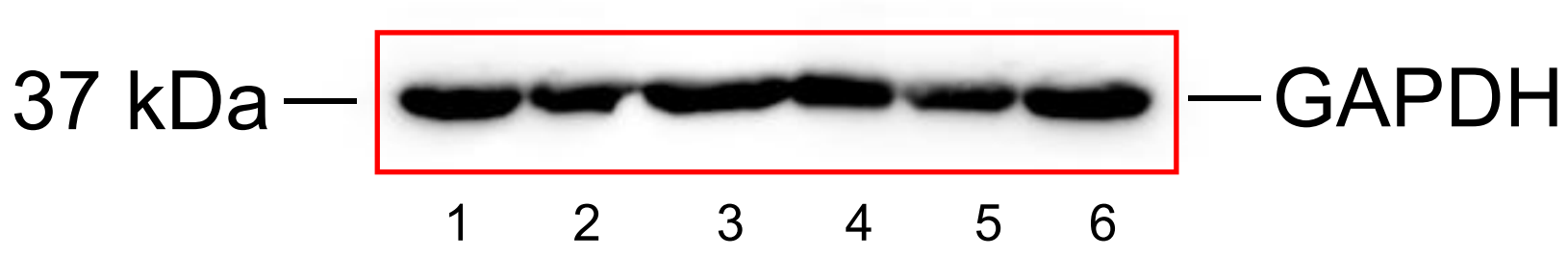

Full and uncropped western blot for Fig 2F, Chemiluminescence (ECL)

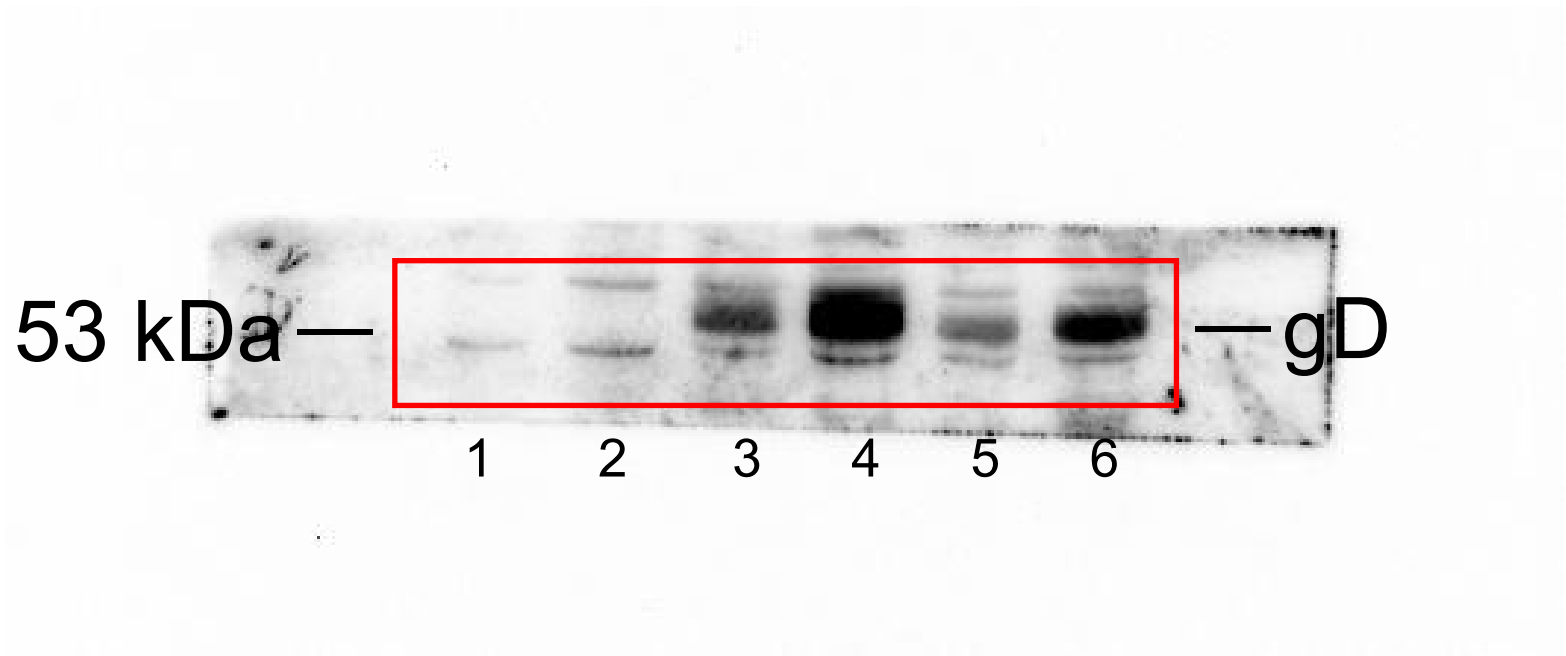


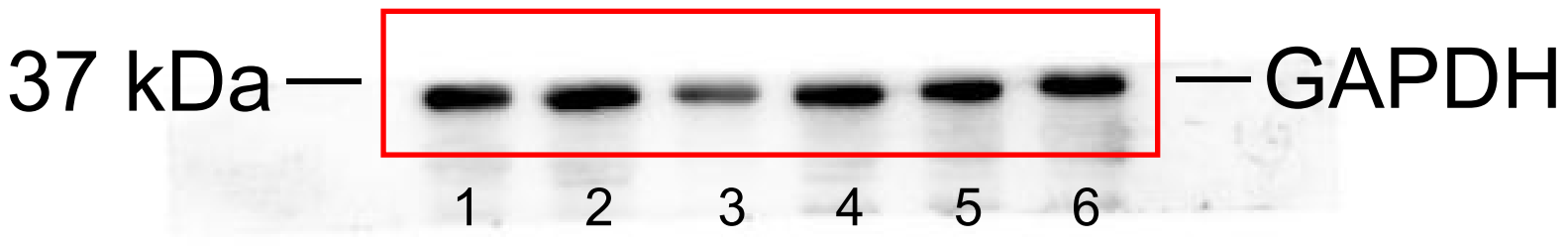

Full and uncropped western blot for Fig 2H, Chemiluminescence (ECL)

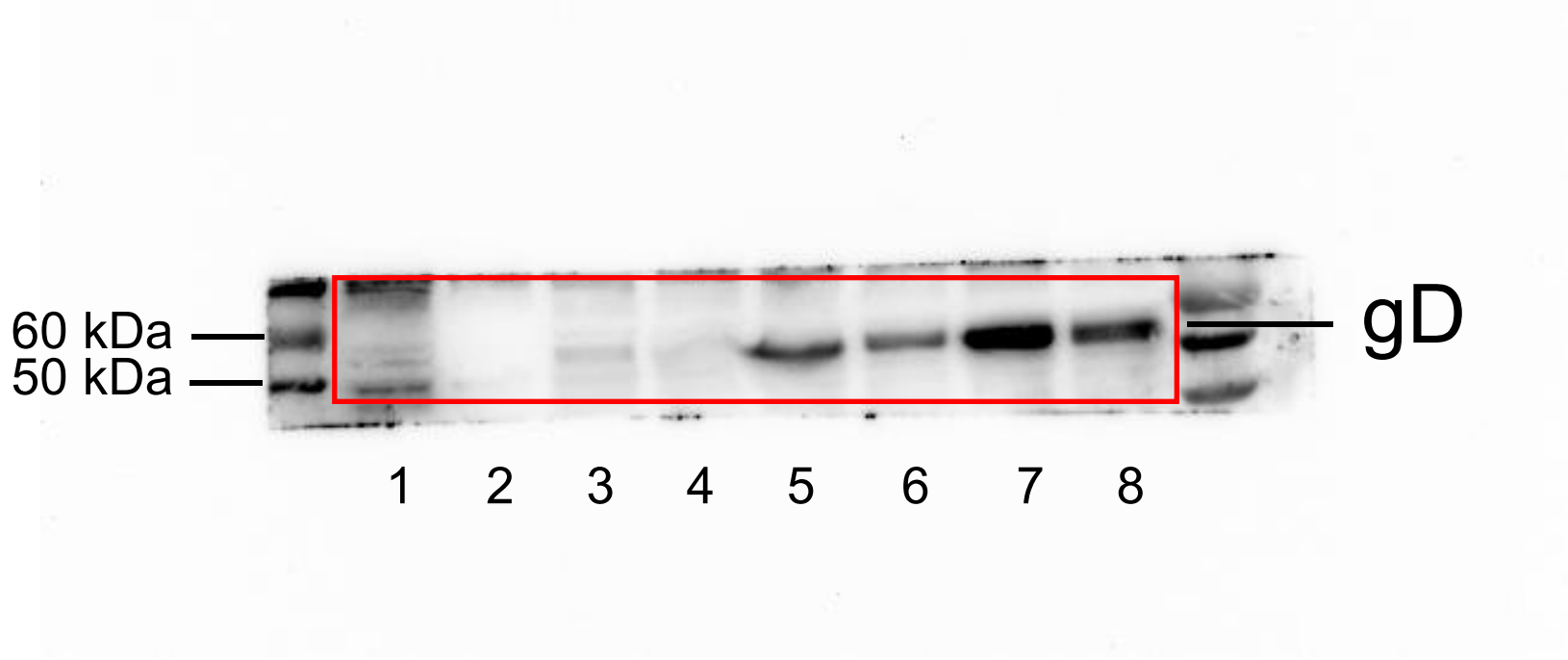


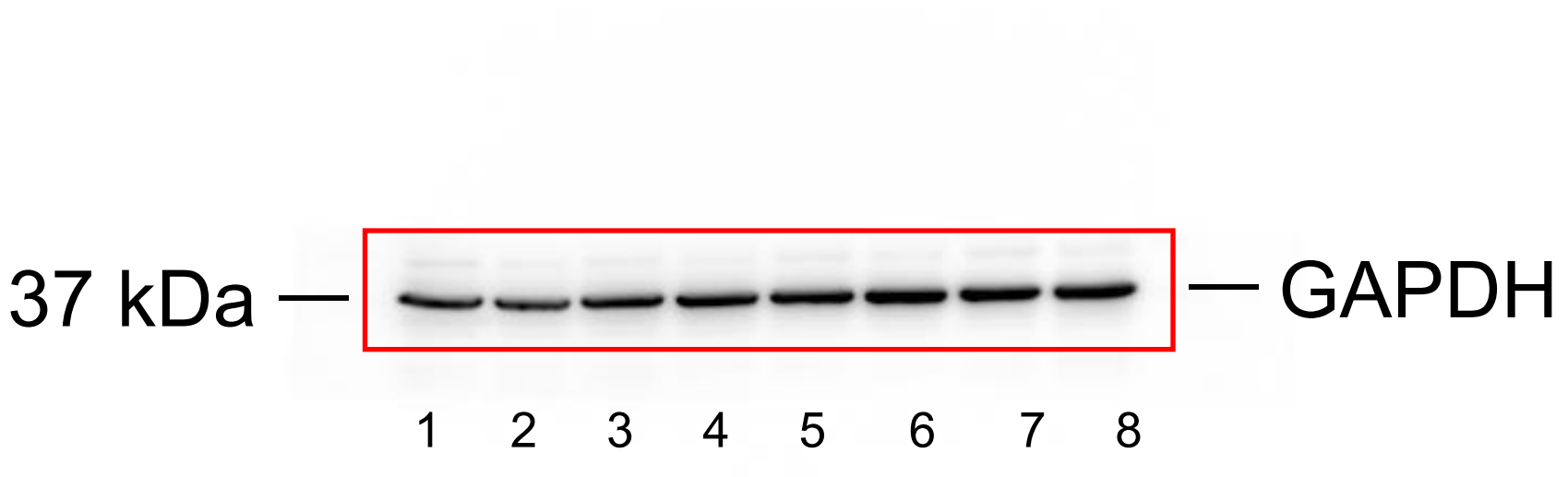

Full and uncropped western blot for Fig 3H, Chemiluminescence (ECL)

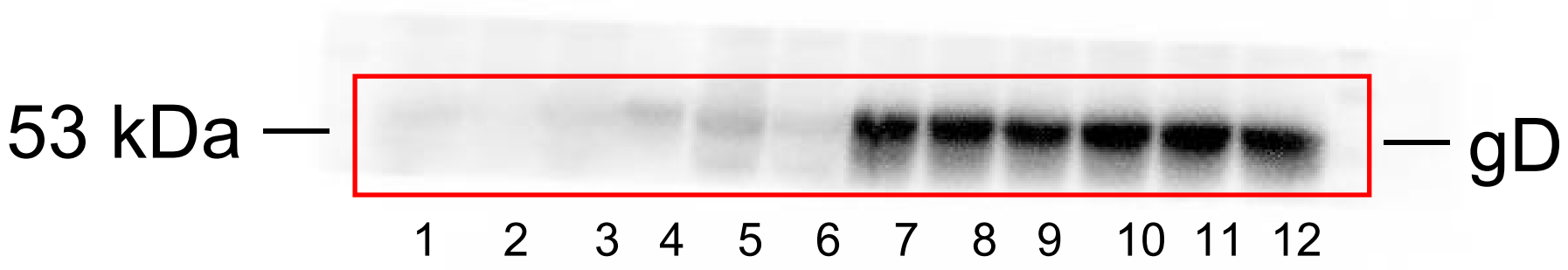


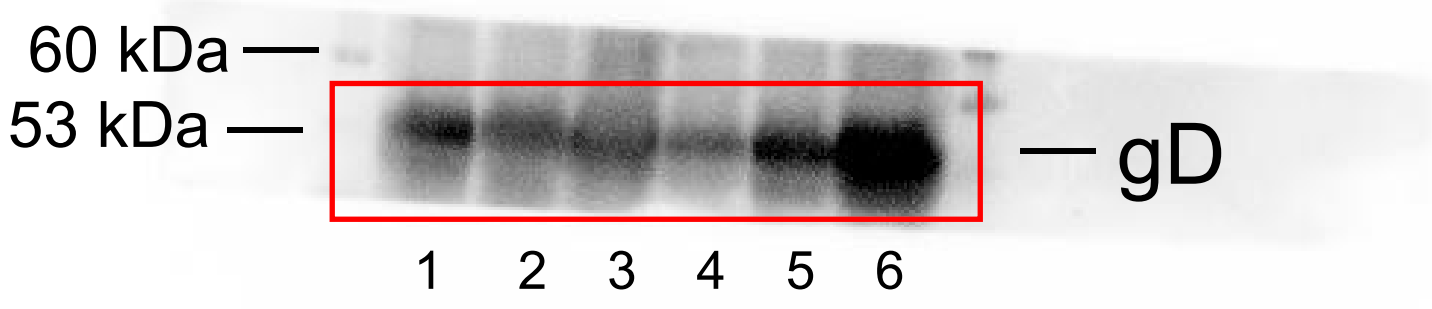


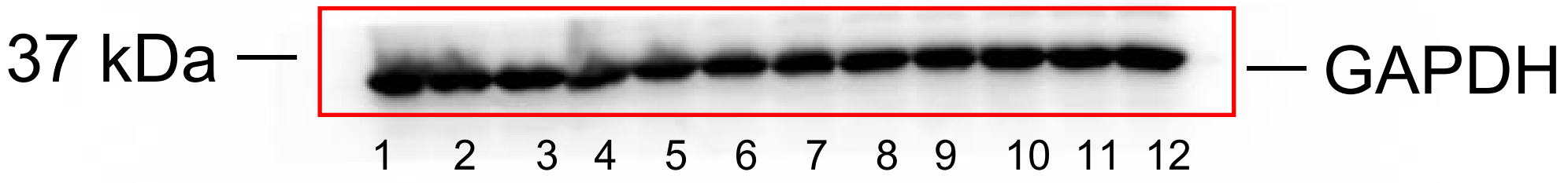


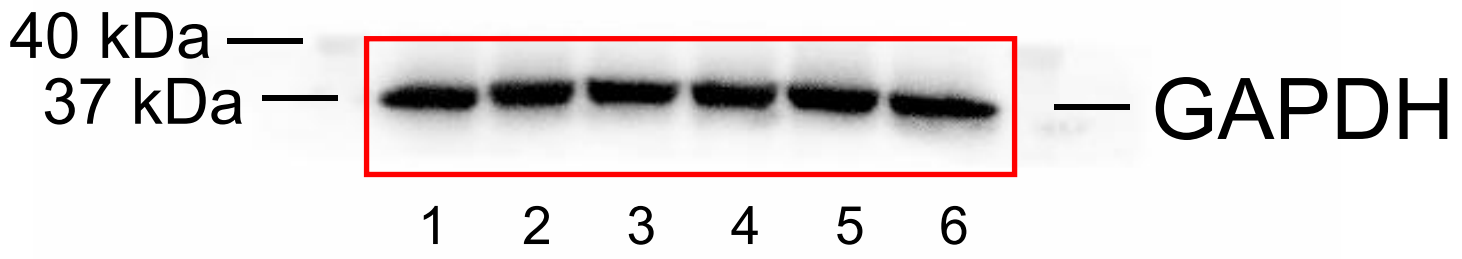

Full and uncropped western blot for Fig 4F and G, Chemiluminescence (ECL)

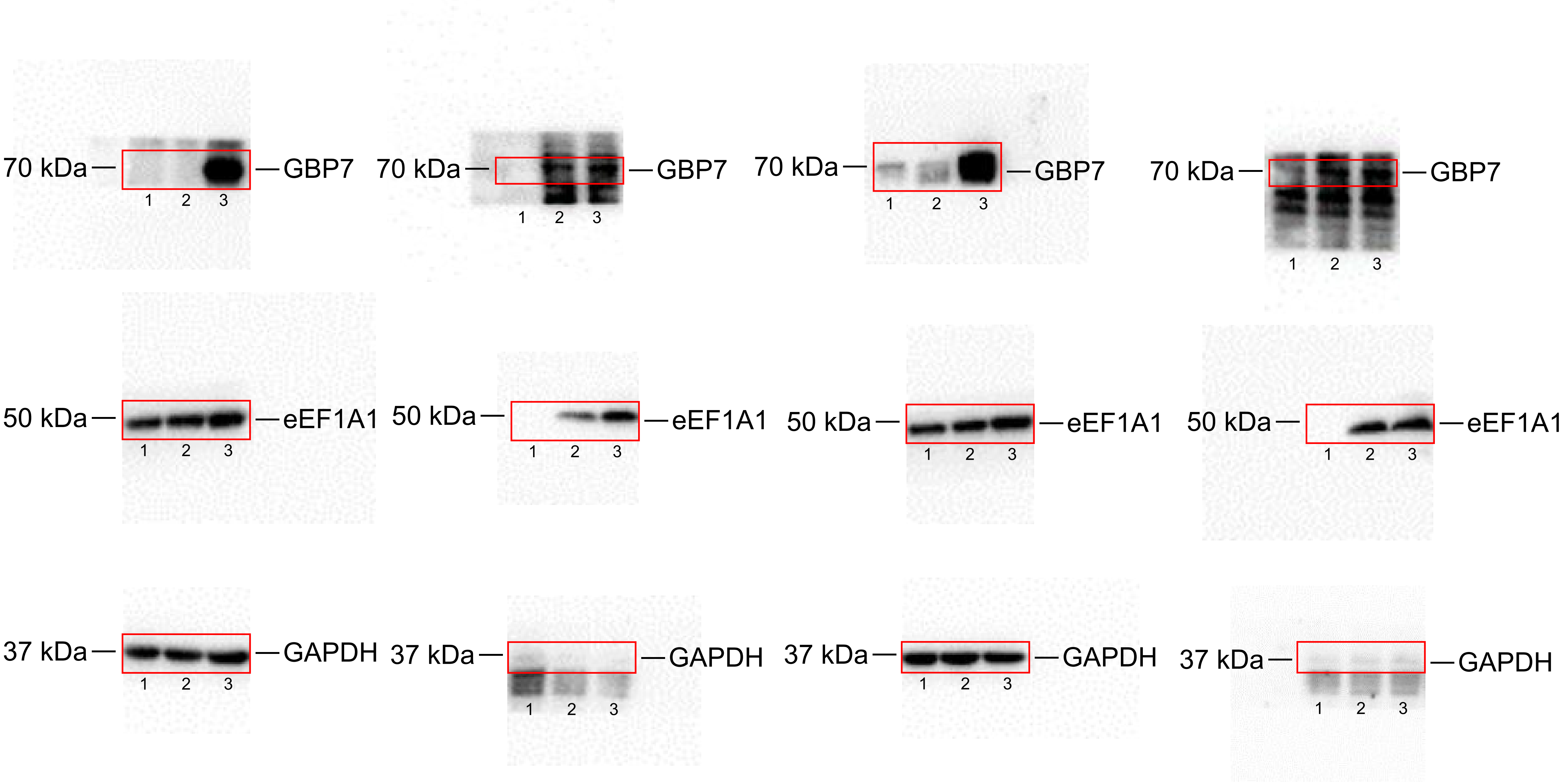

# Full and uncropped western blot for Fig 4J, Chemiluminescence (ECL)

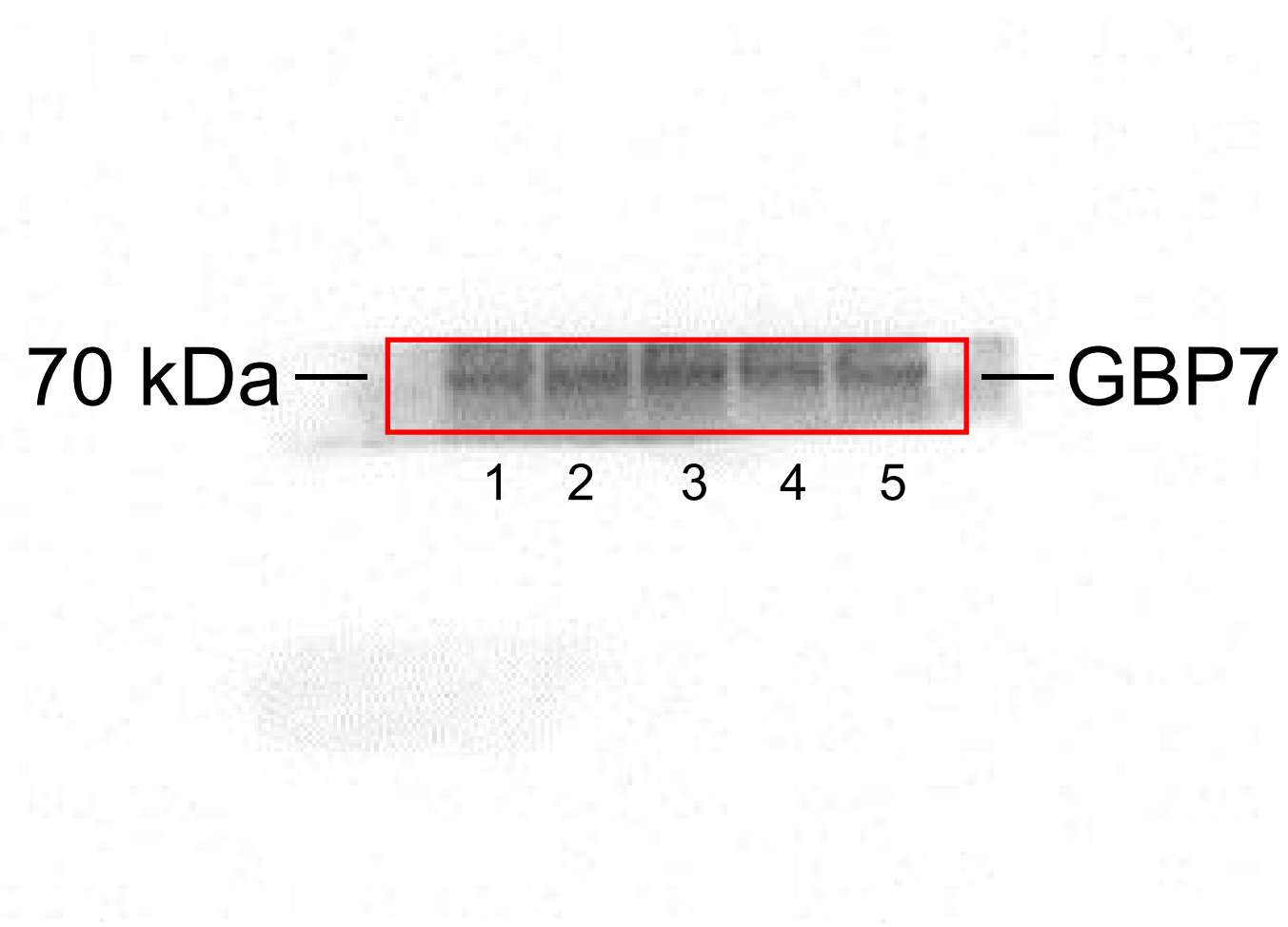


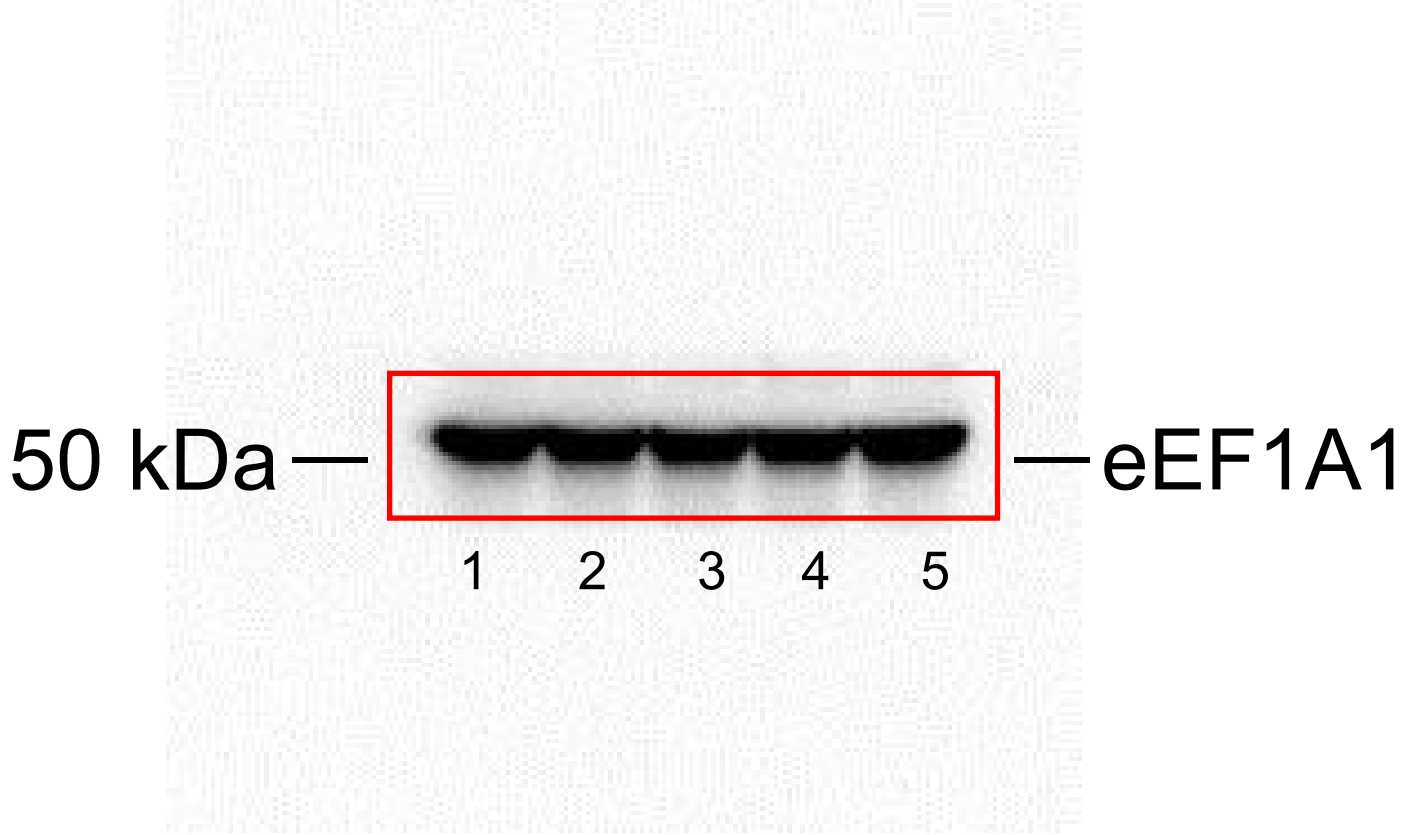


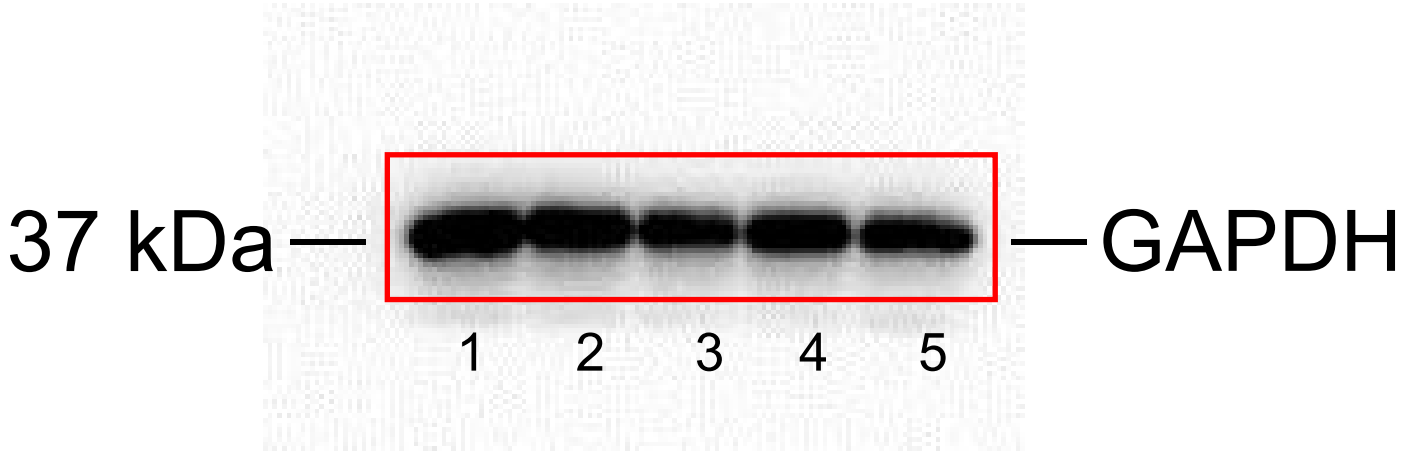


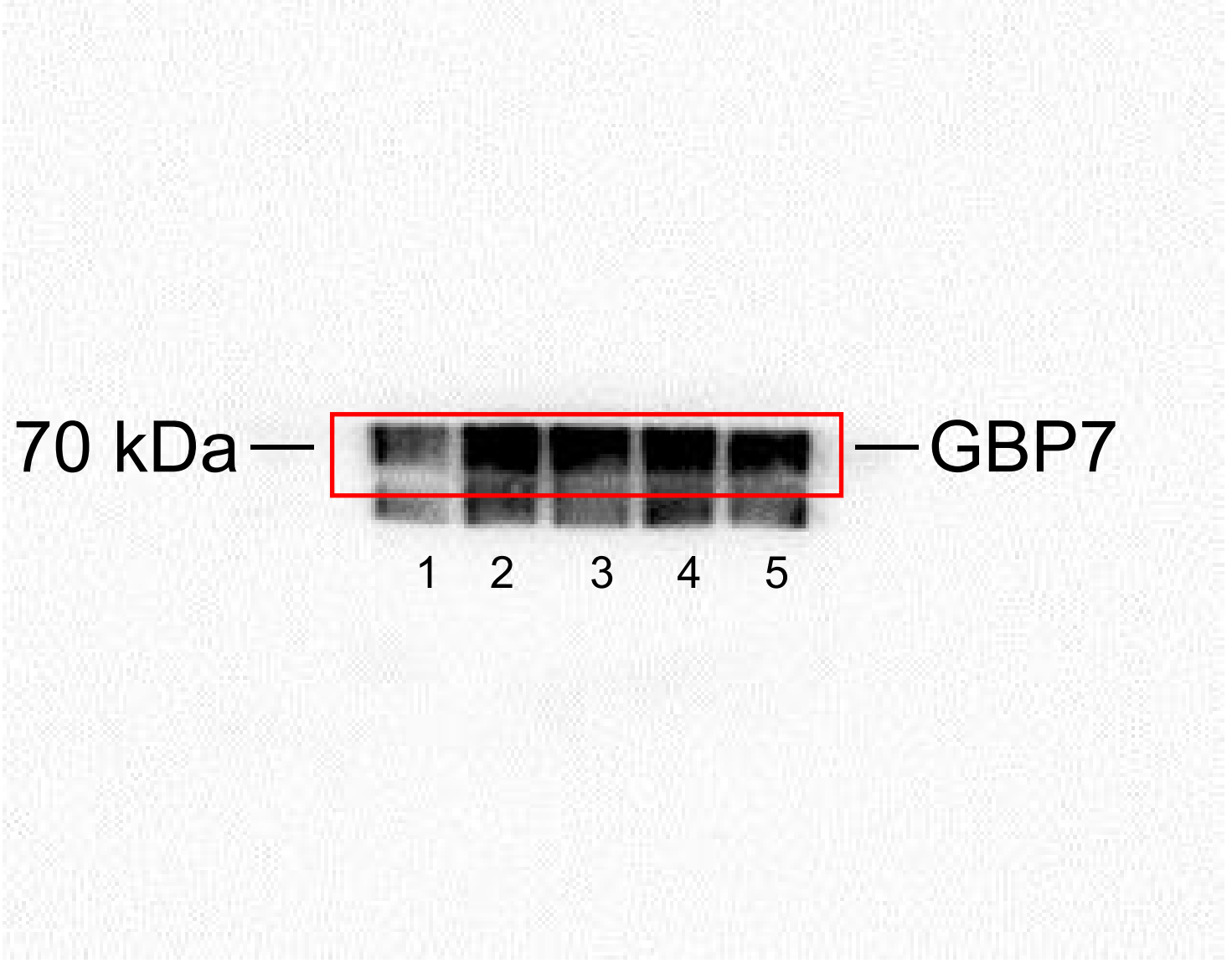


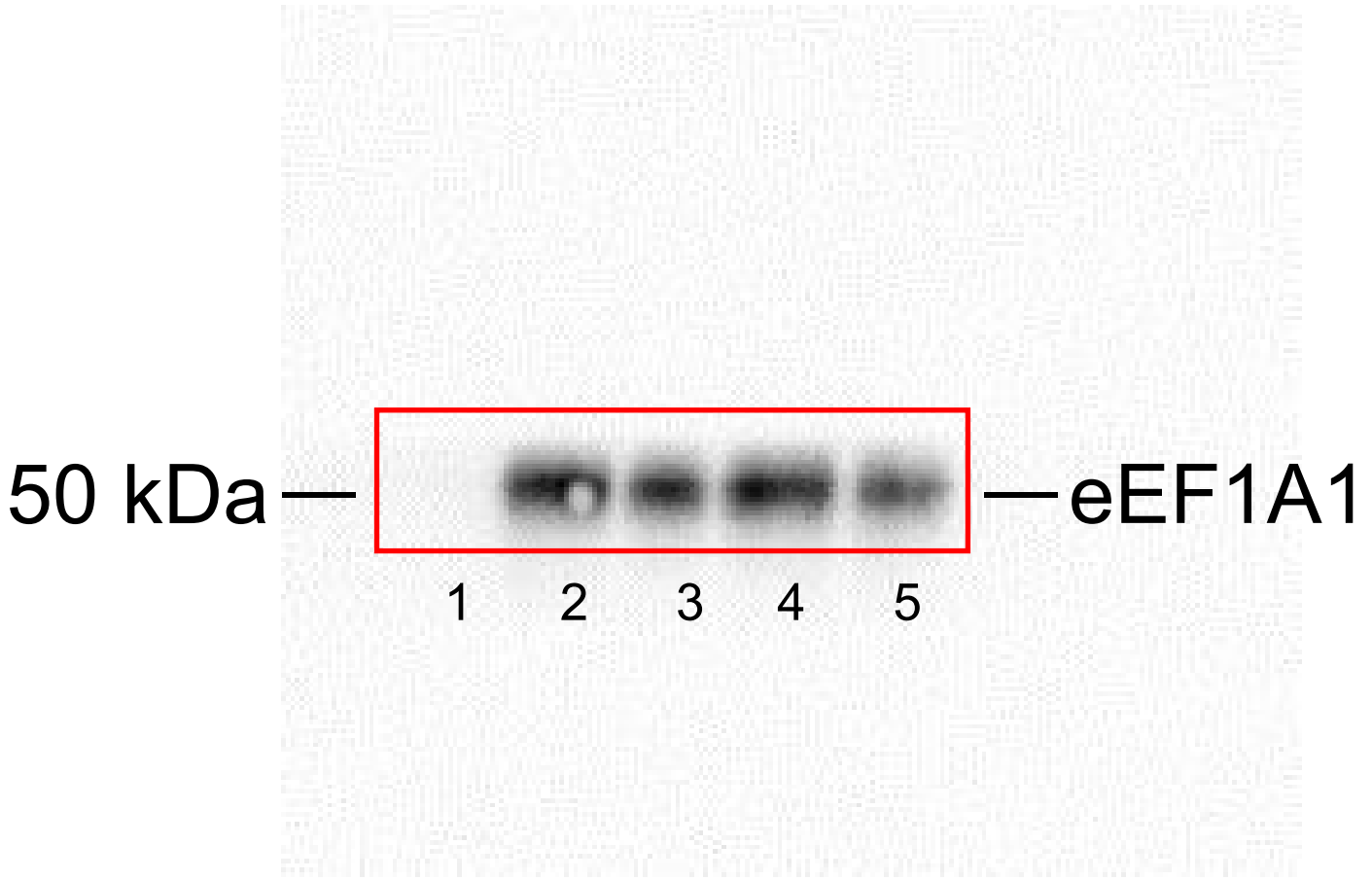


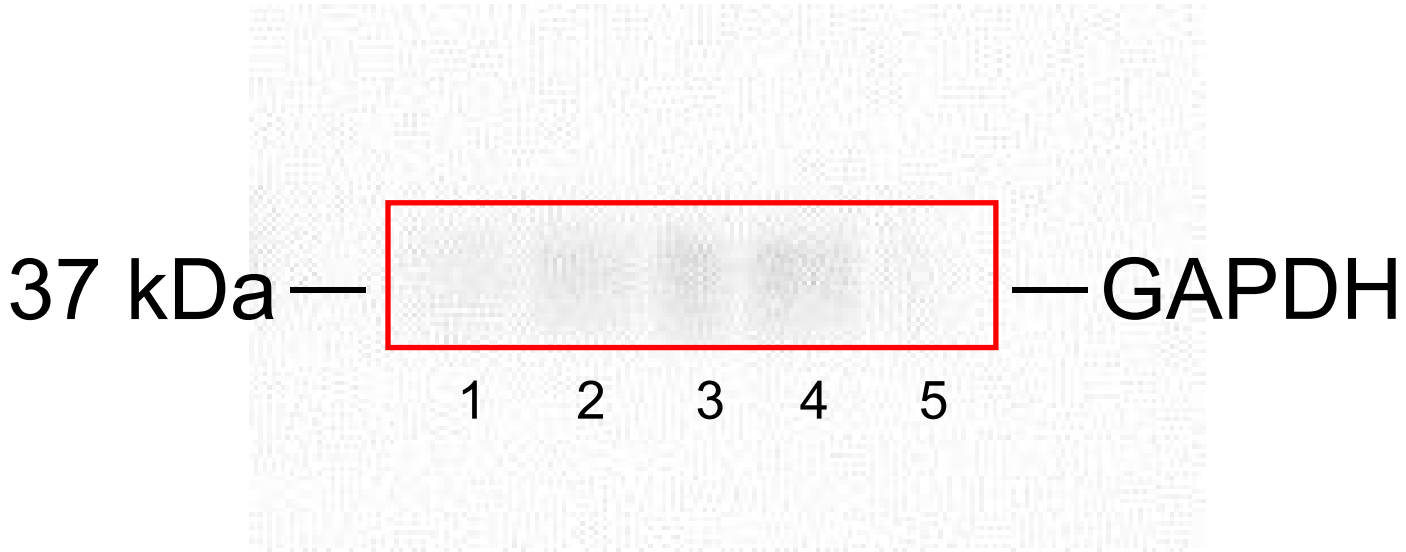

Full and uncropped western blot for Fig 5D, Chemiluminescence (ECL)

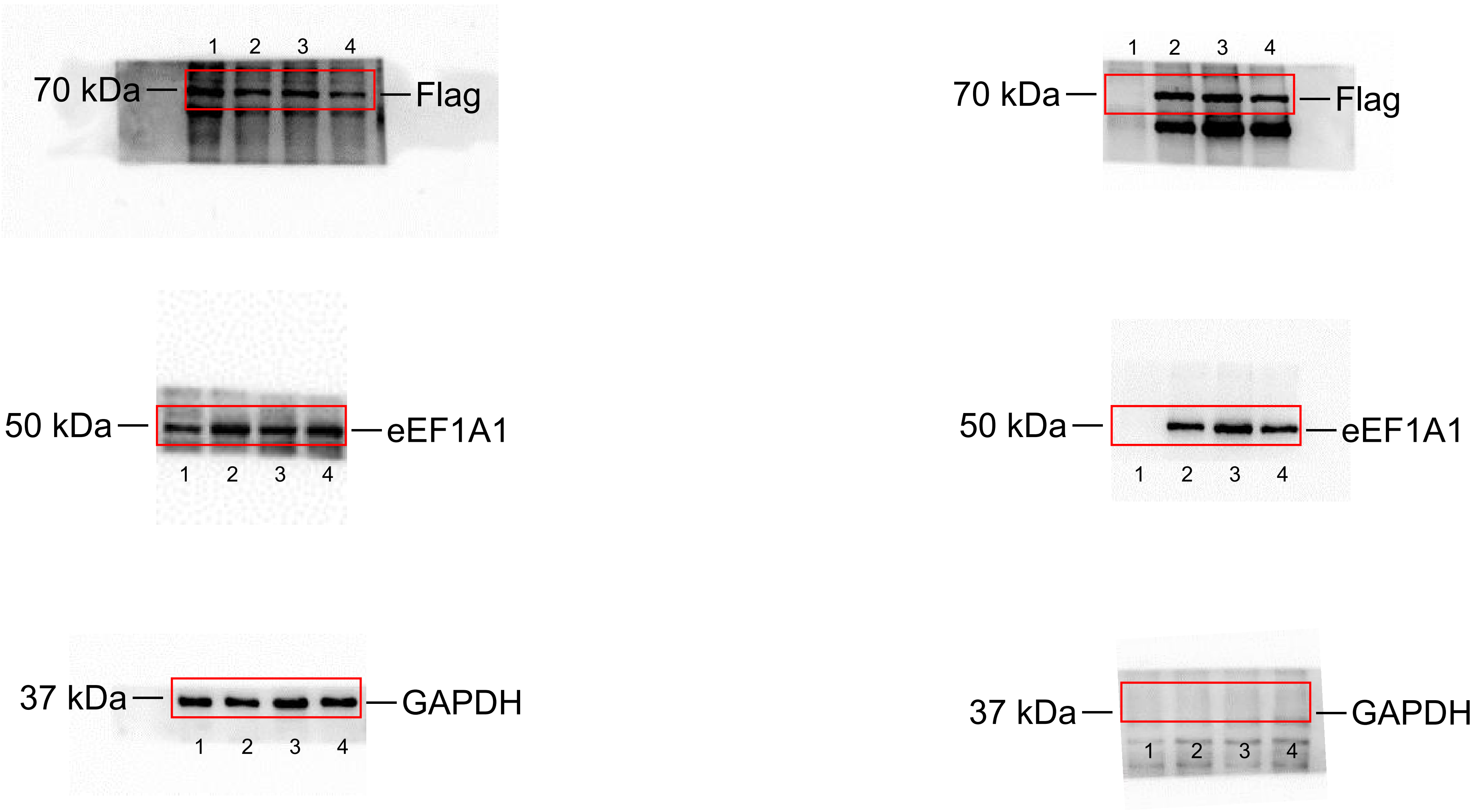

Full and uncropped western blot for Fig 5E, Chemiluminescence (ECL)

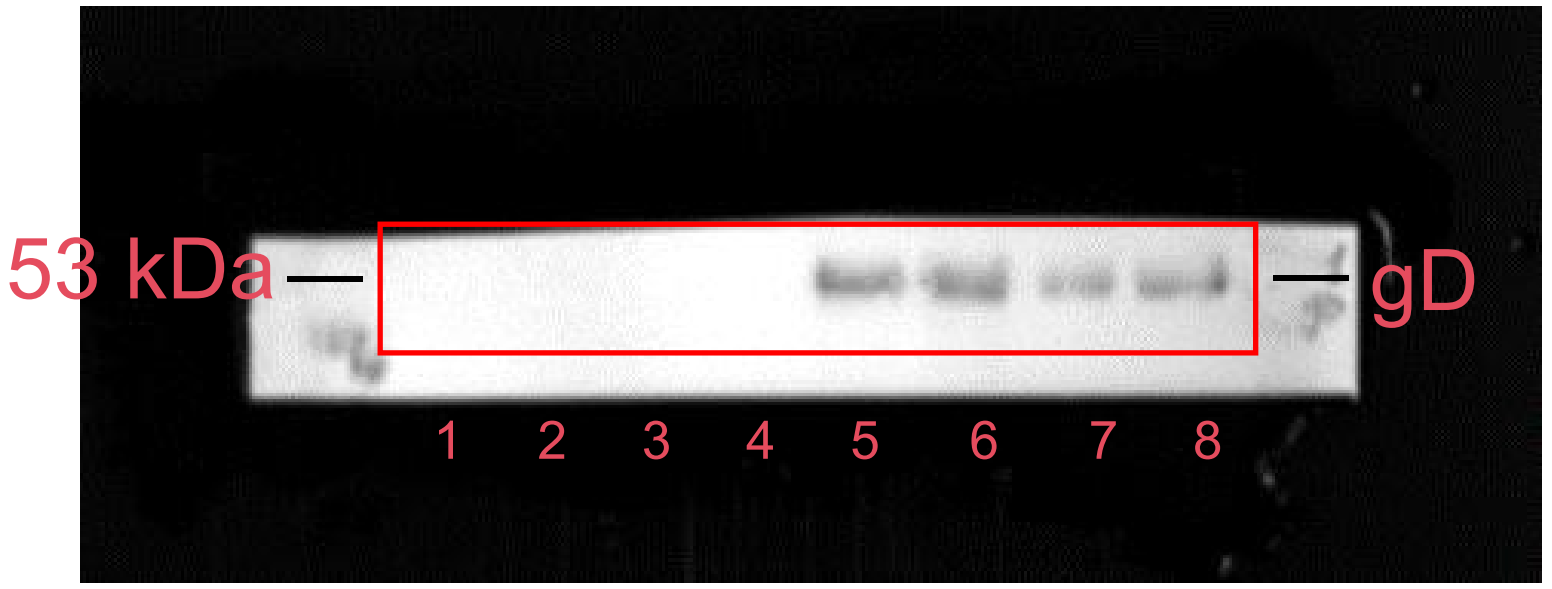


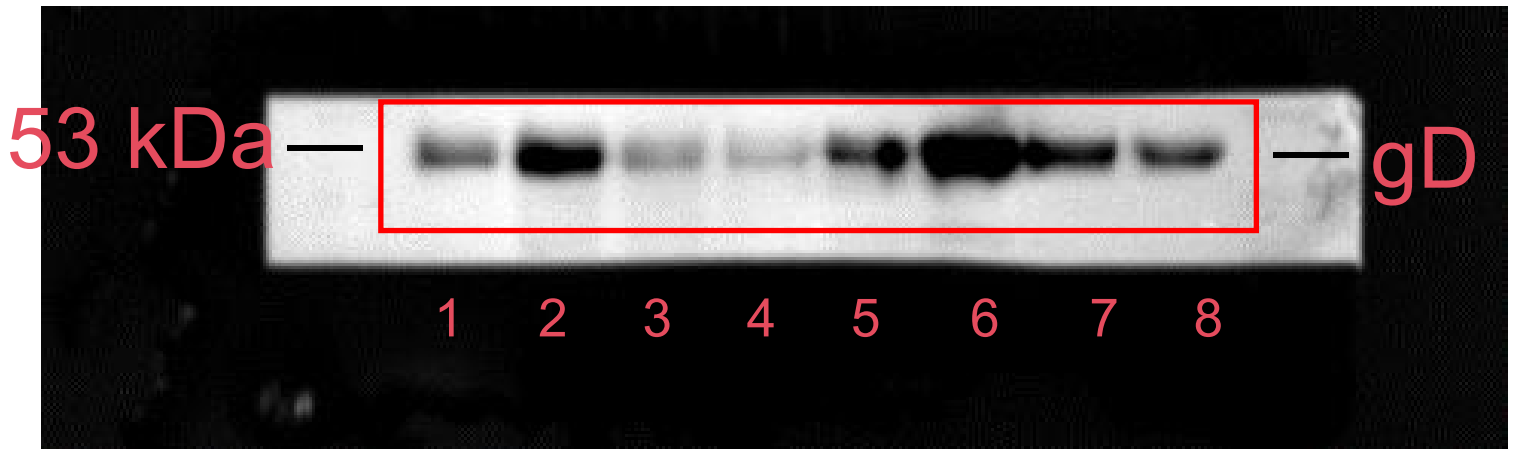


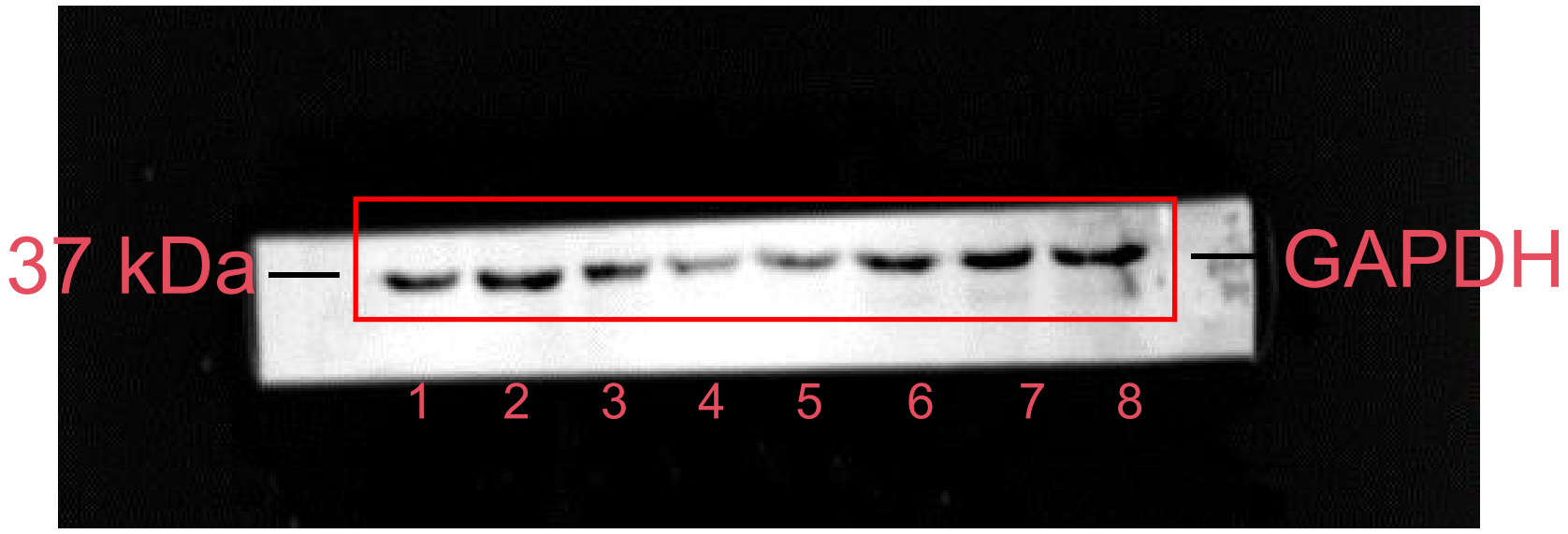


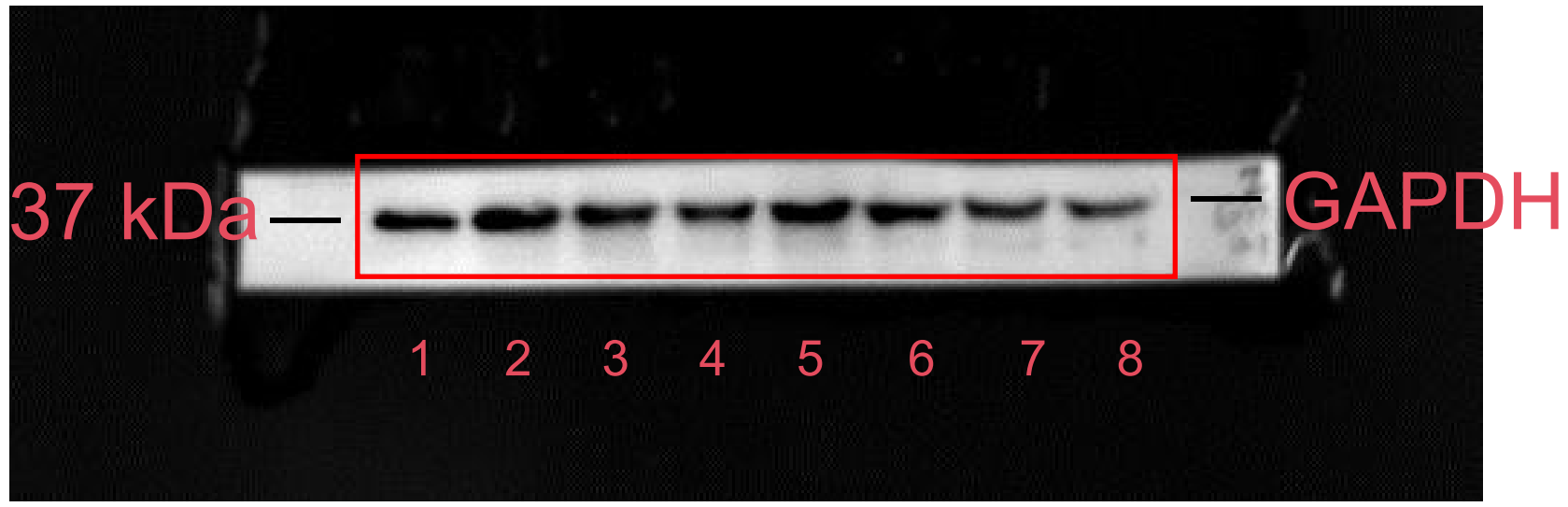

Full and uncropped western blot for Fig 5G, Chemiluminescence (ECL)

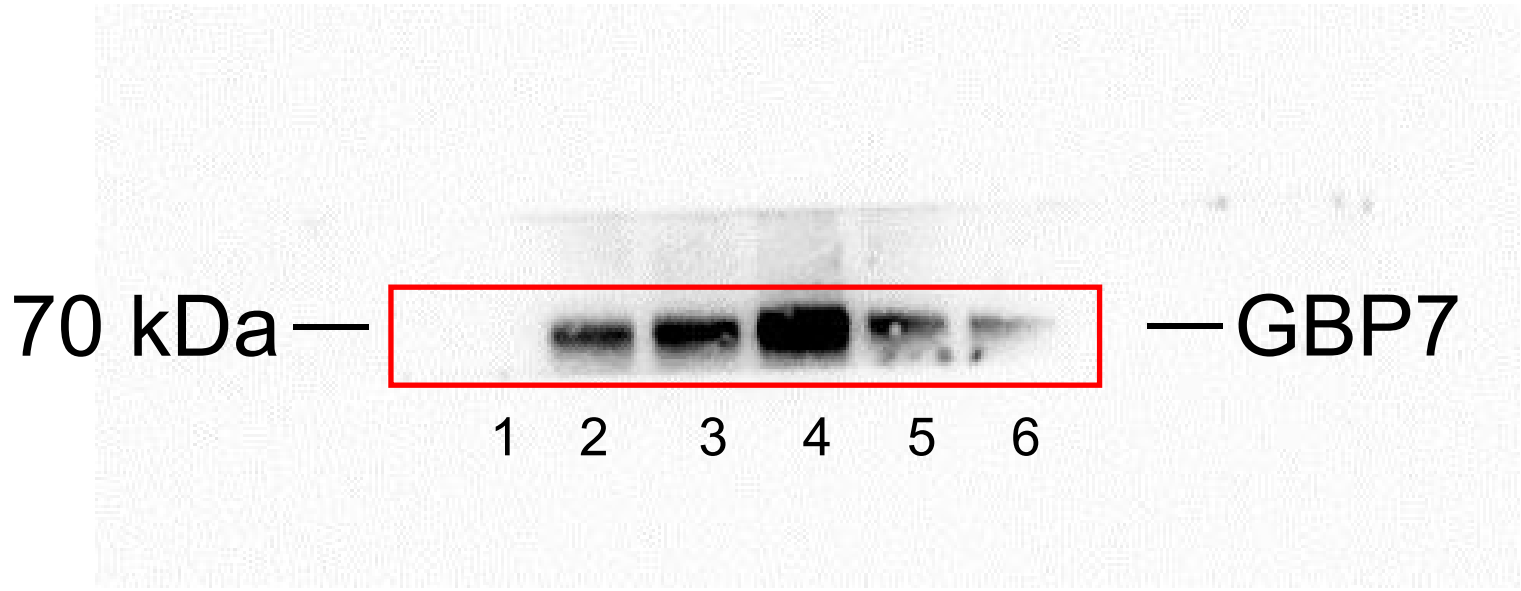


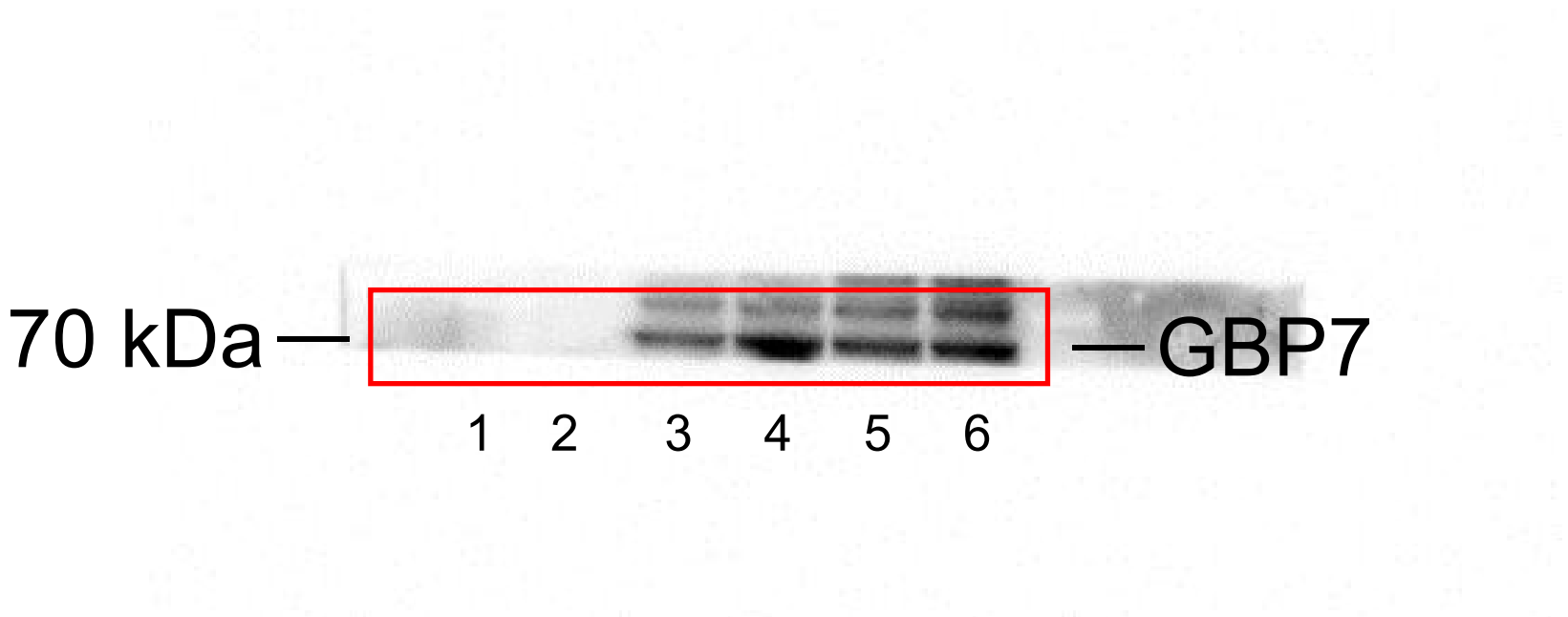


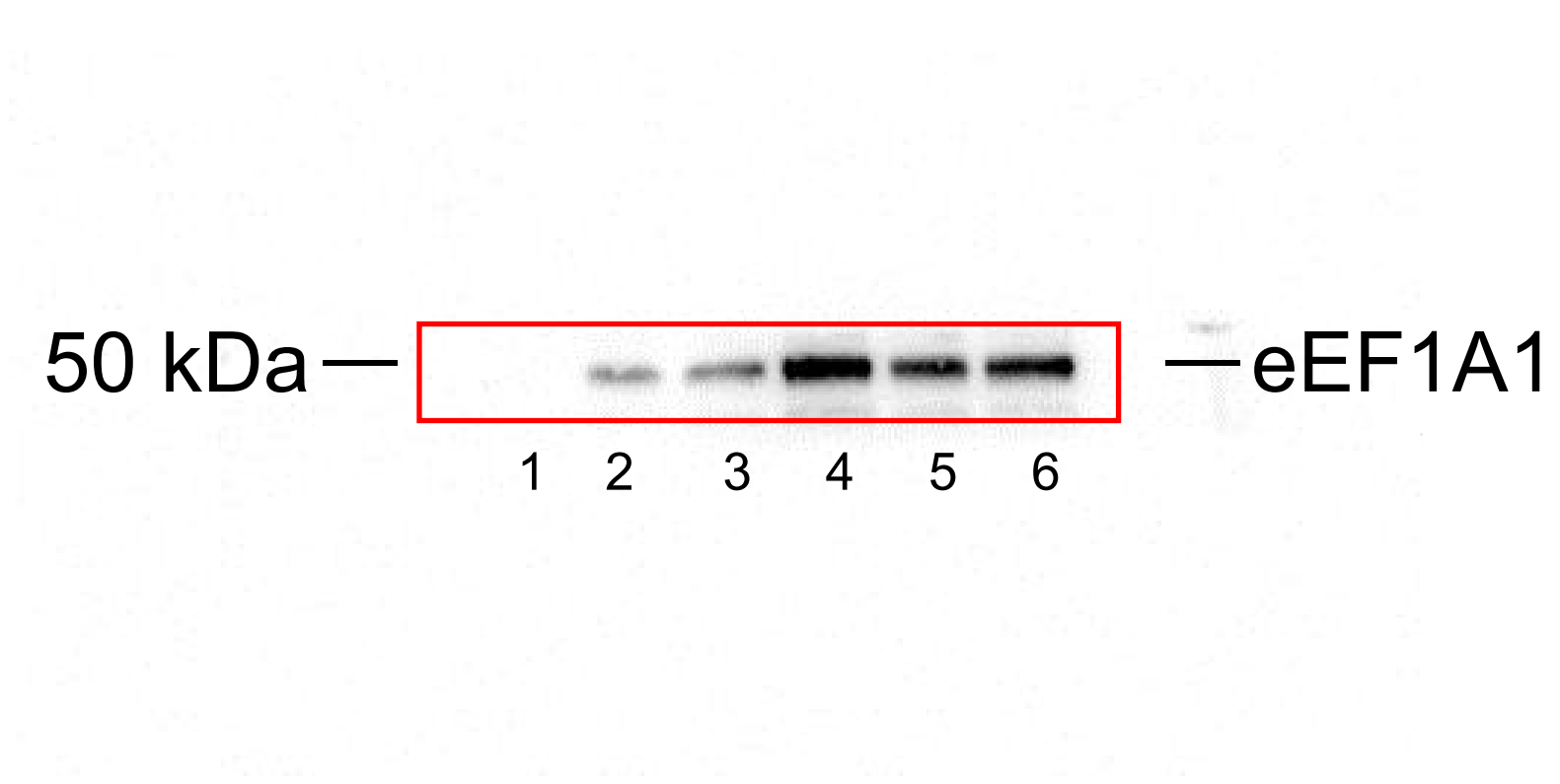


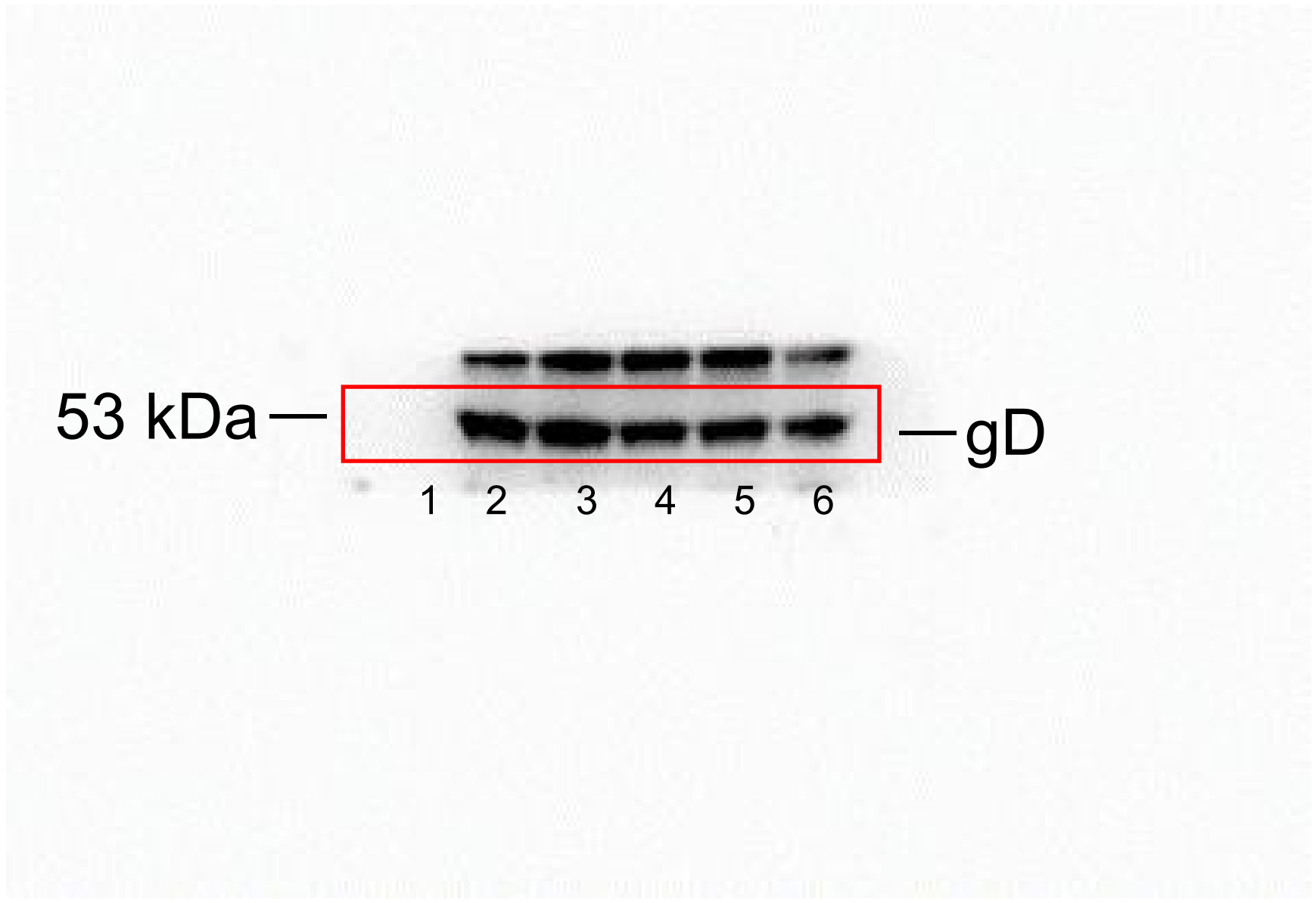

Full and uncropped western blot for Fig 5H, Chemiluminescence (ECL)

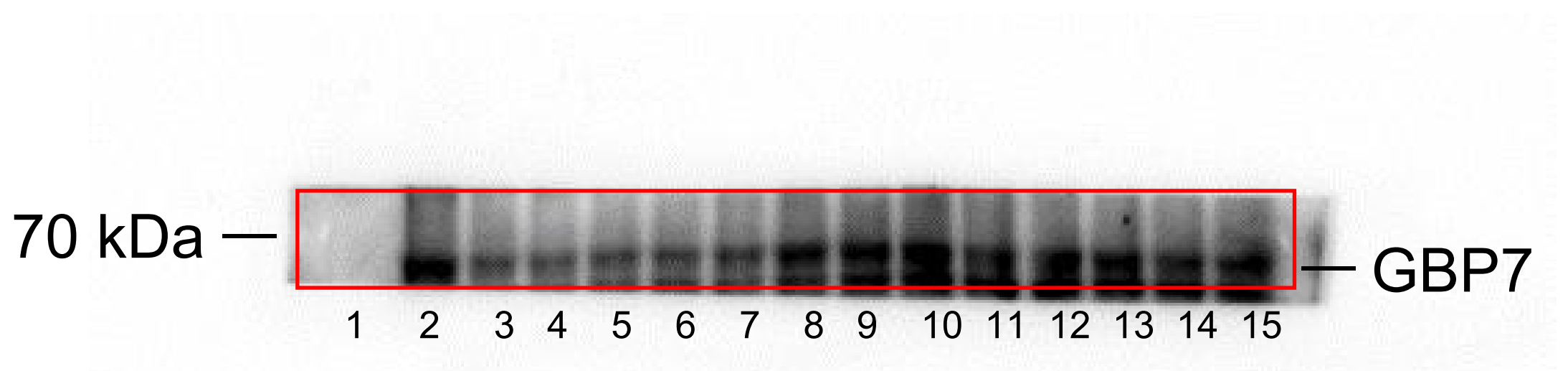


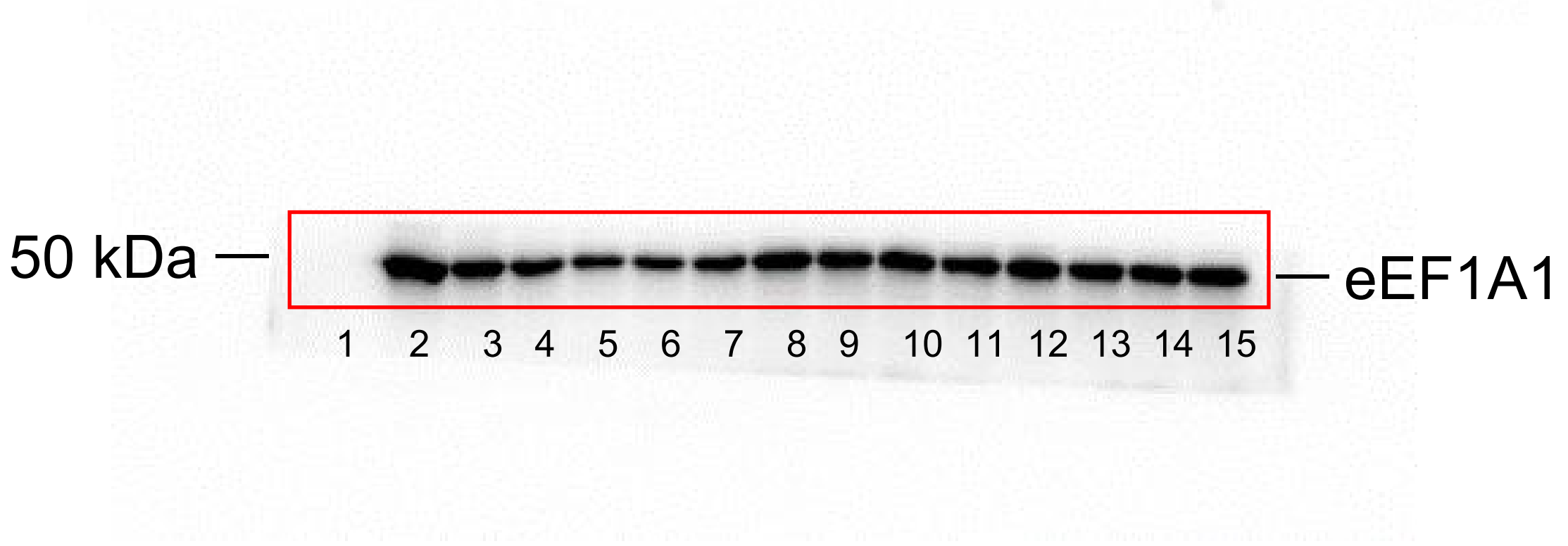

Full and uncropped western blot for Fig 5I, Chemiluminescence (ECL)

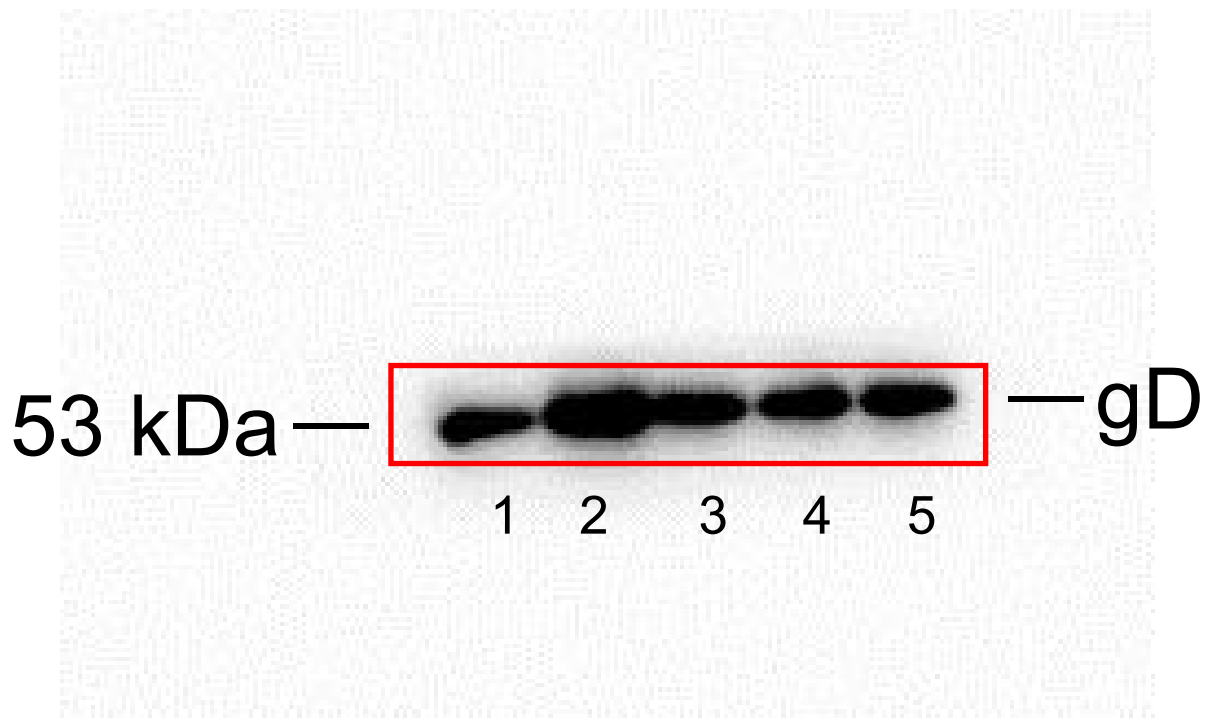


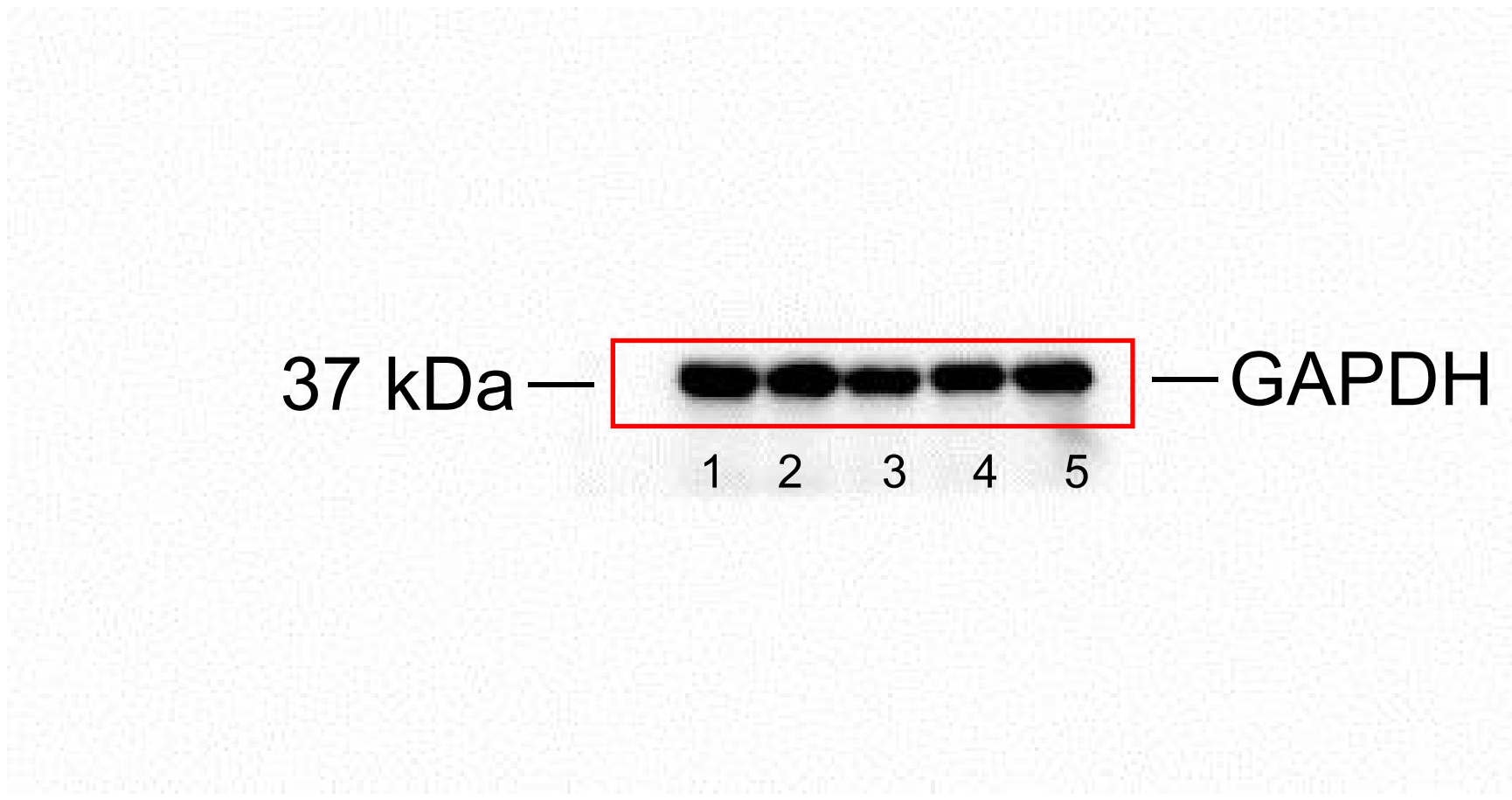

Full and uncropped western blot for Fig 5J, Chemiluminescence (ECL)

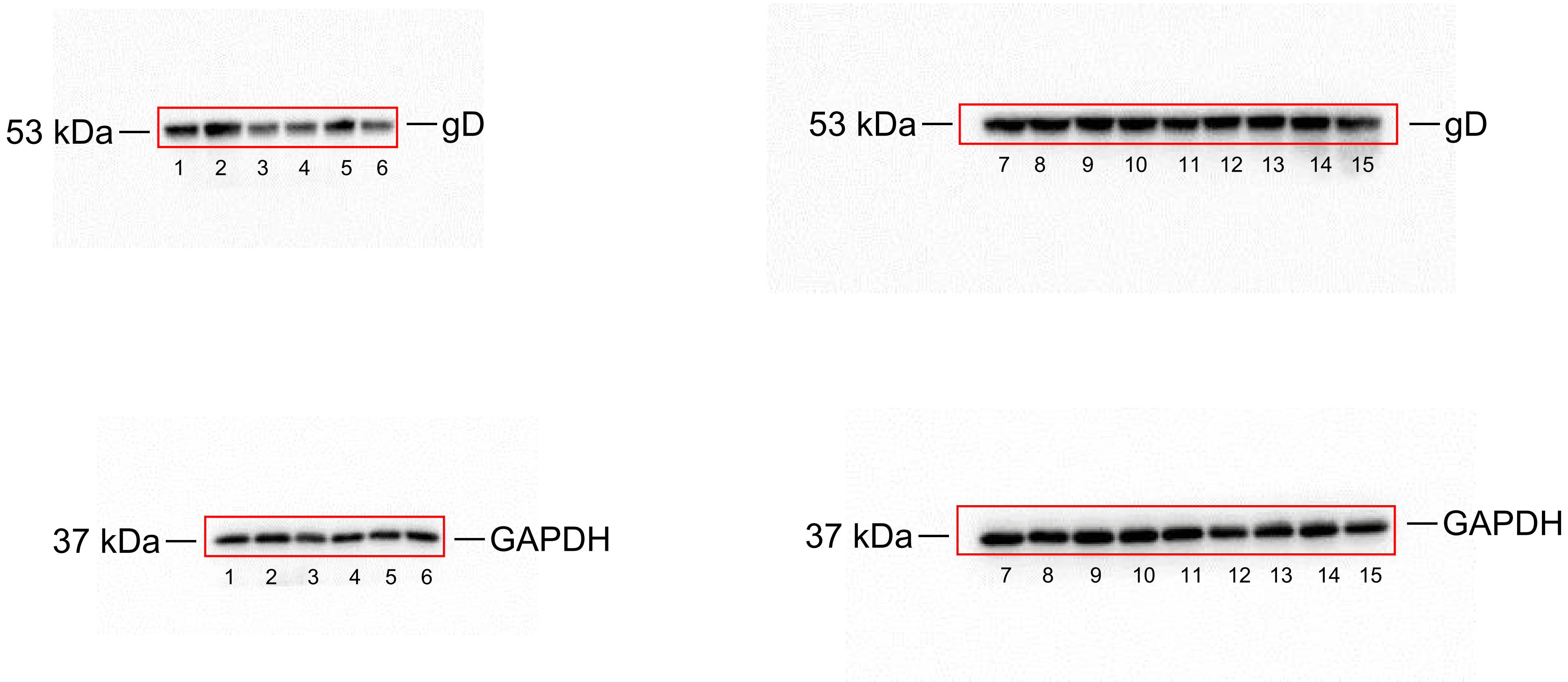

Full and uncropped western blot for Fig 6B, Chemiluminescence (ECL)

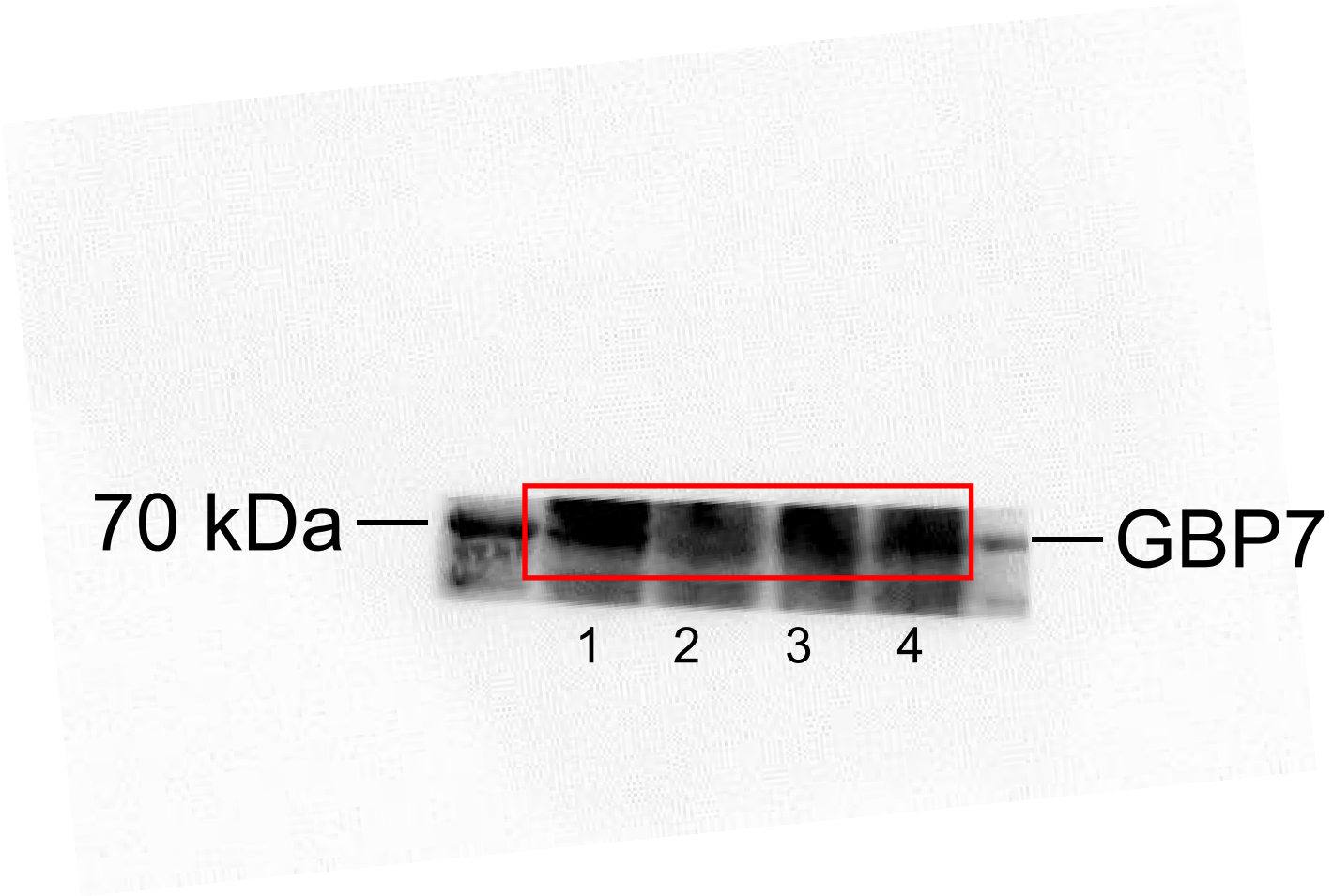


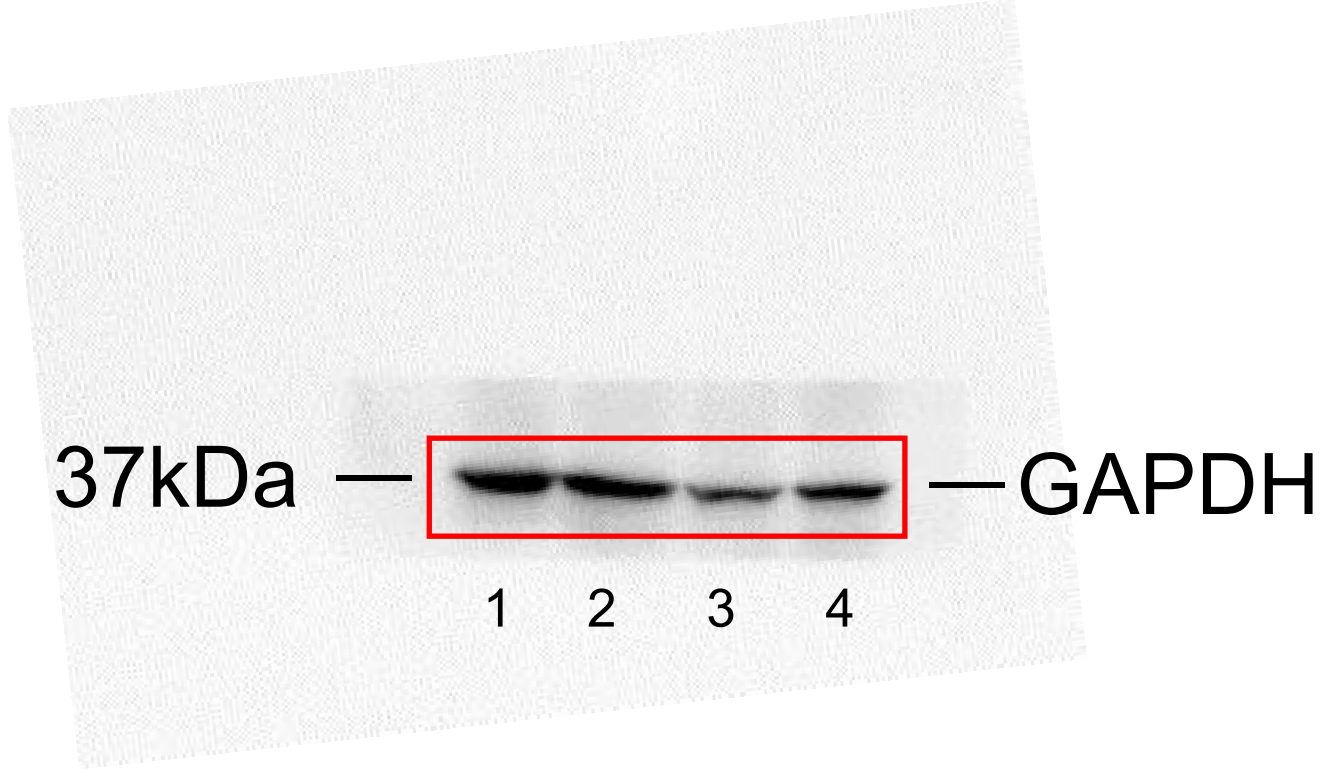

Full and uncropped western blot for Fig 6E, Chemiluminescence (ECL)

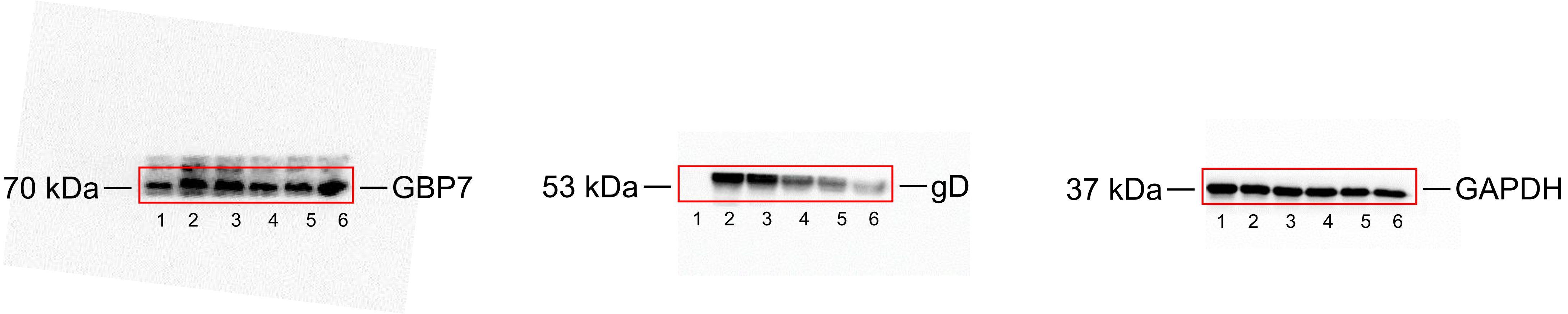

Full and uncropped western blot for Fig 6I, Chemiluminescence (ECL)

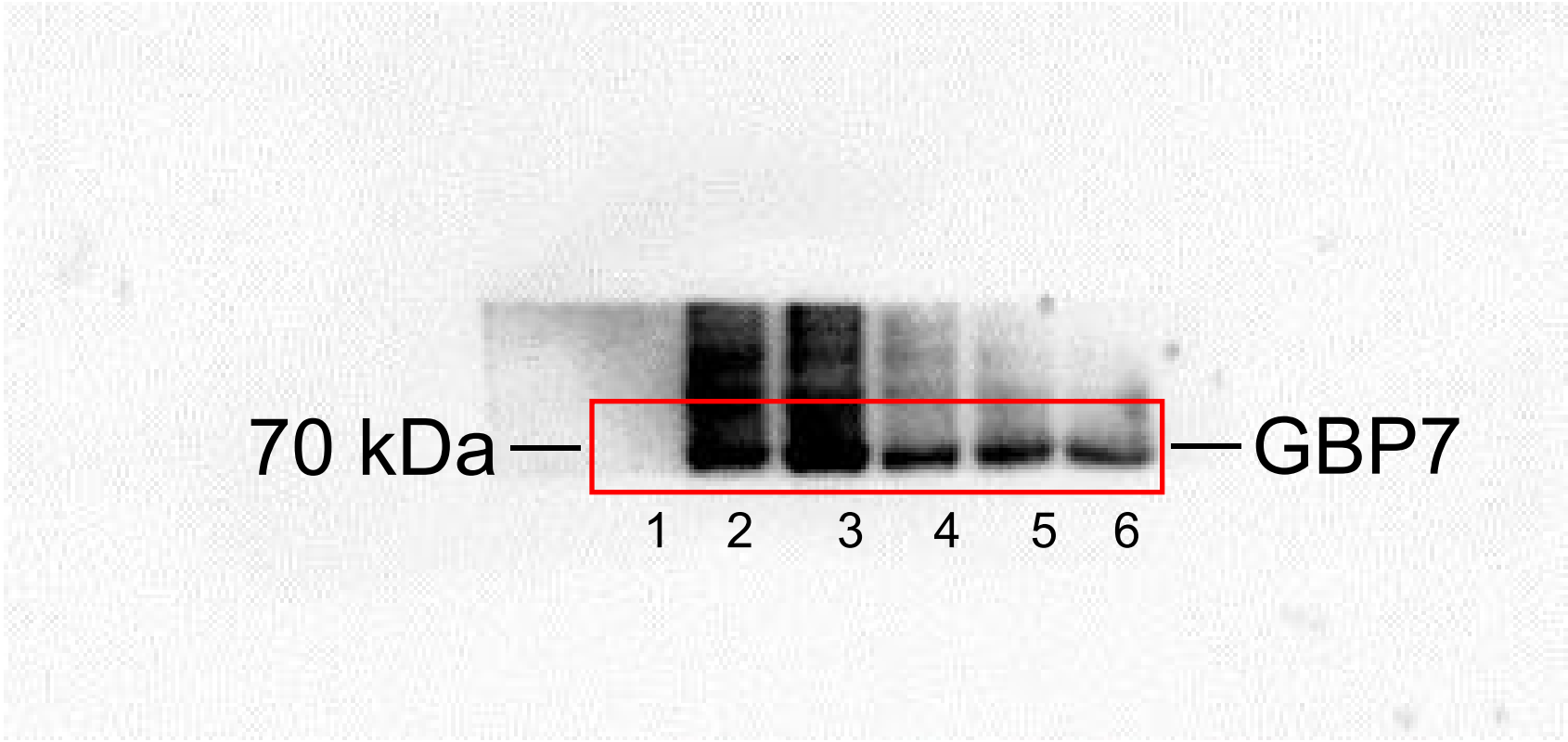


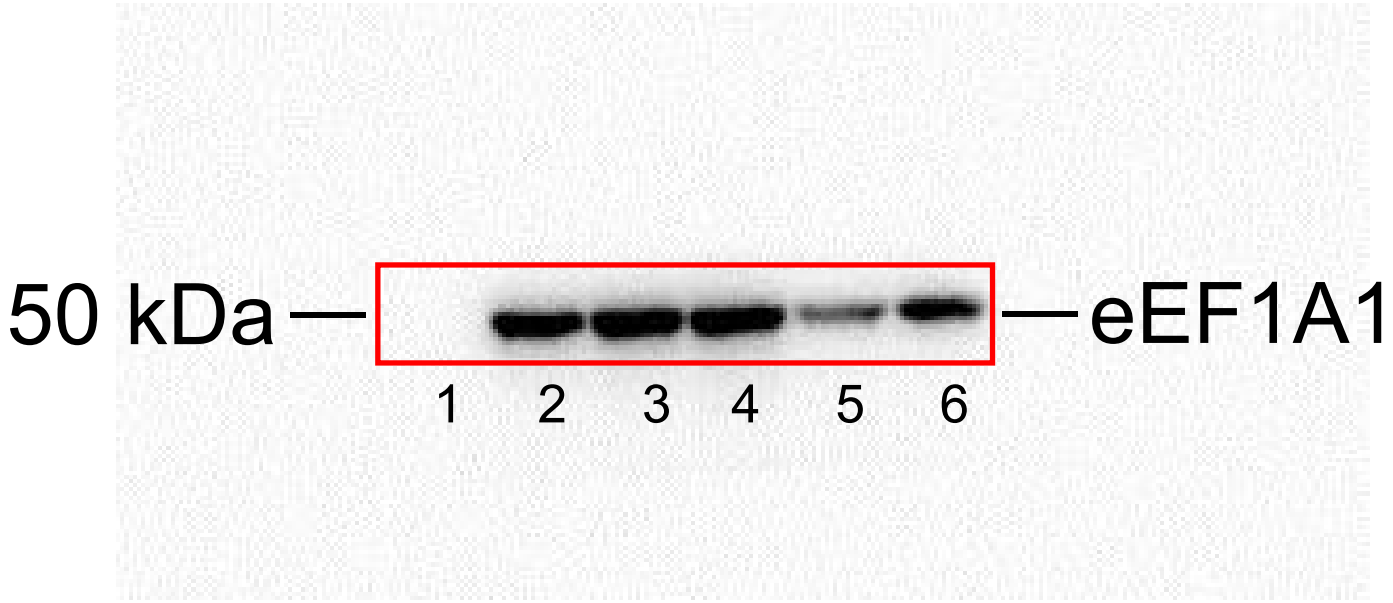

Full and uncropped western blot for Fig 6J, Chemiluminescence (ECL)

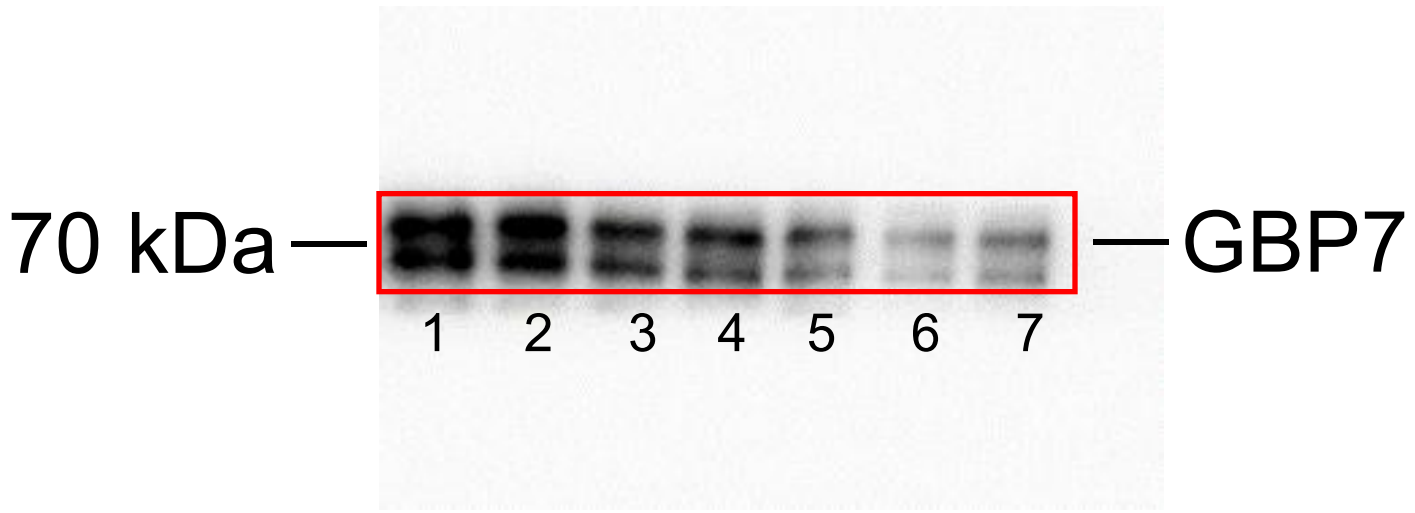


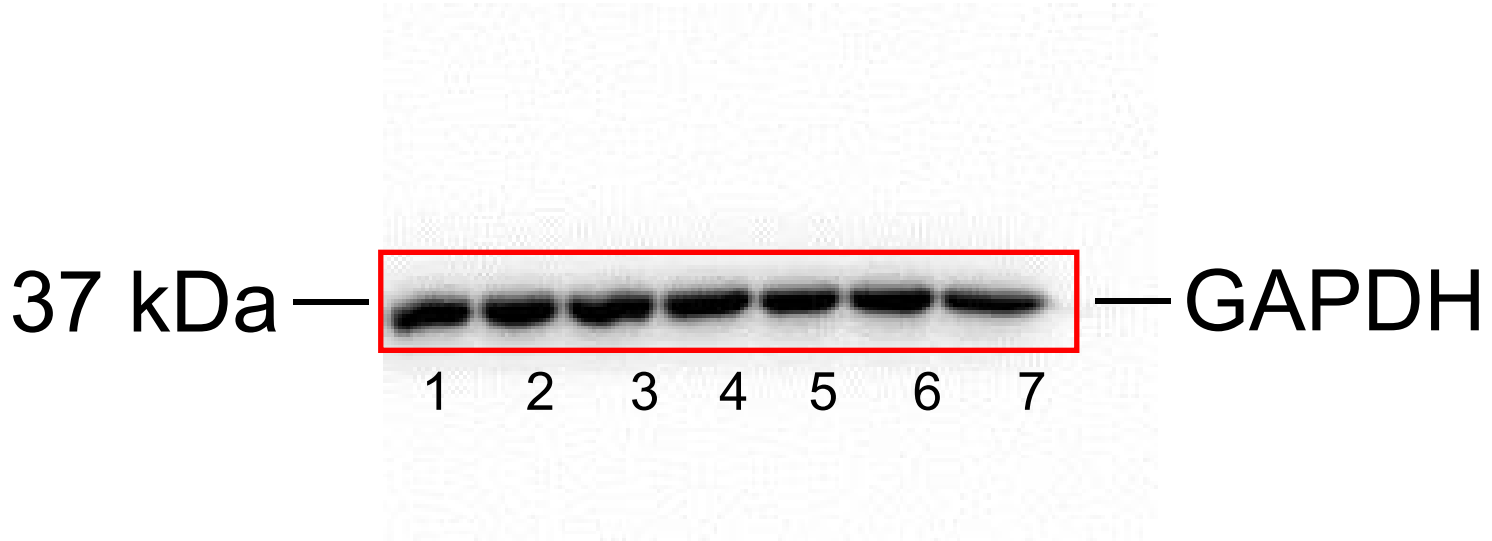

Full and uncropped western blot for Fig 8D, Chemiluminescence (ECL)

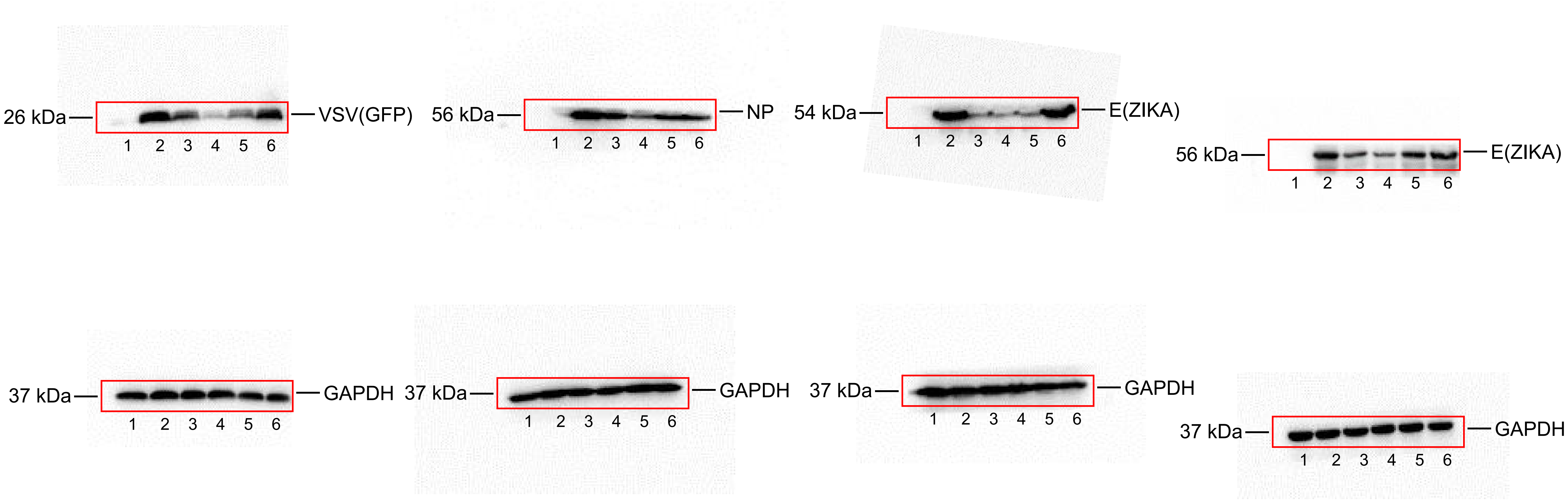

Full and uncropped western blot for S1A Fig, Chemiluminescence (ECL)

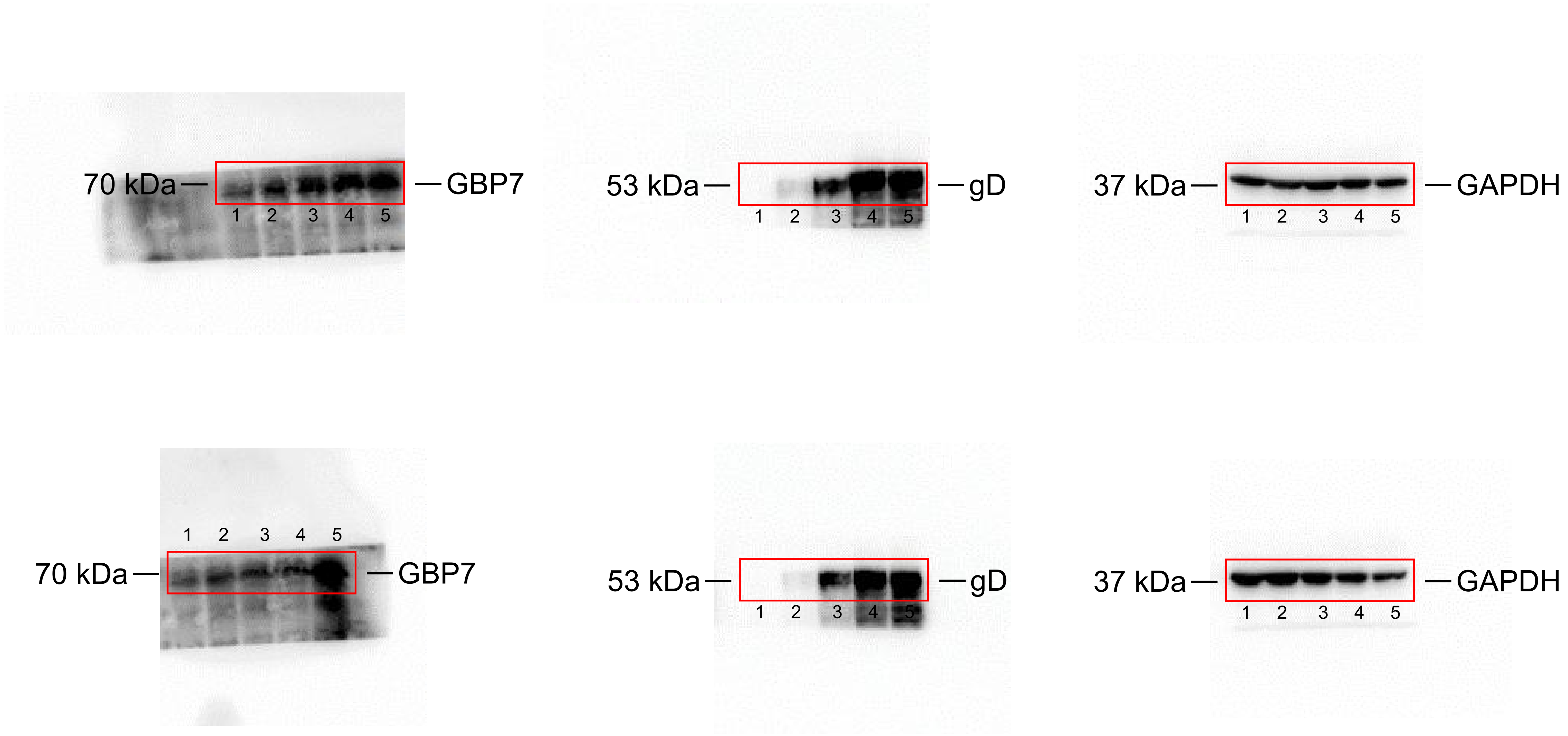

Full and uncropped western blot for S1B Fig, Chemiluminescence (ECL)

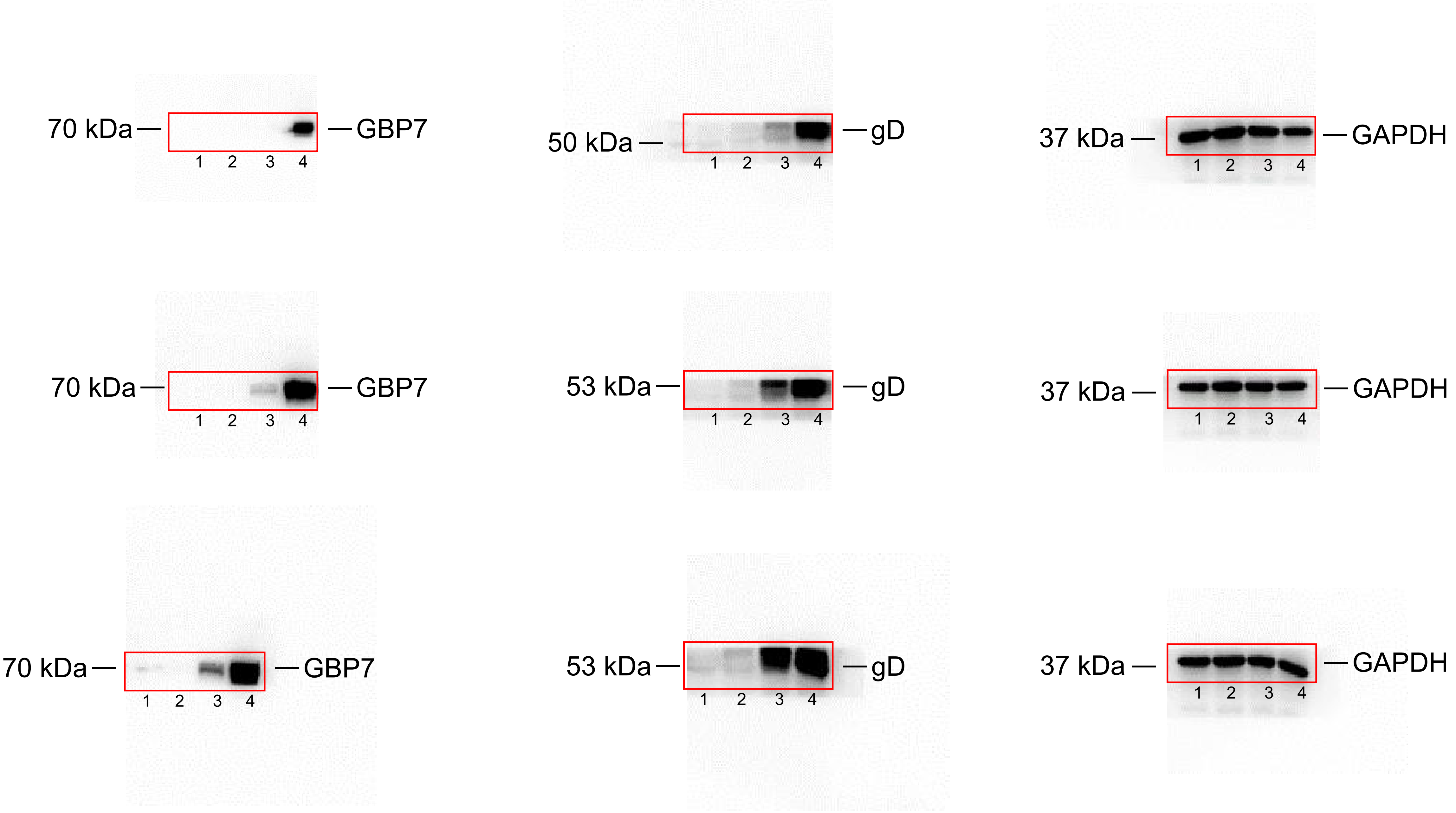

Full and uncropped western blot for S3A Fig, Chemiluminescence (ECL)

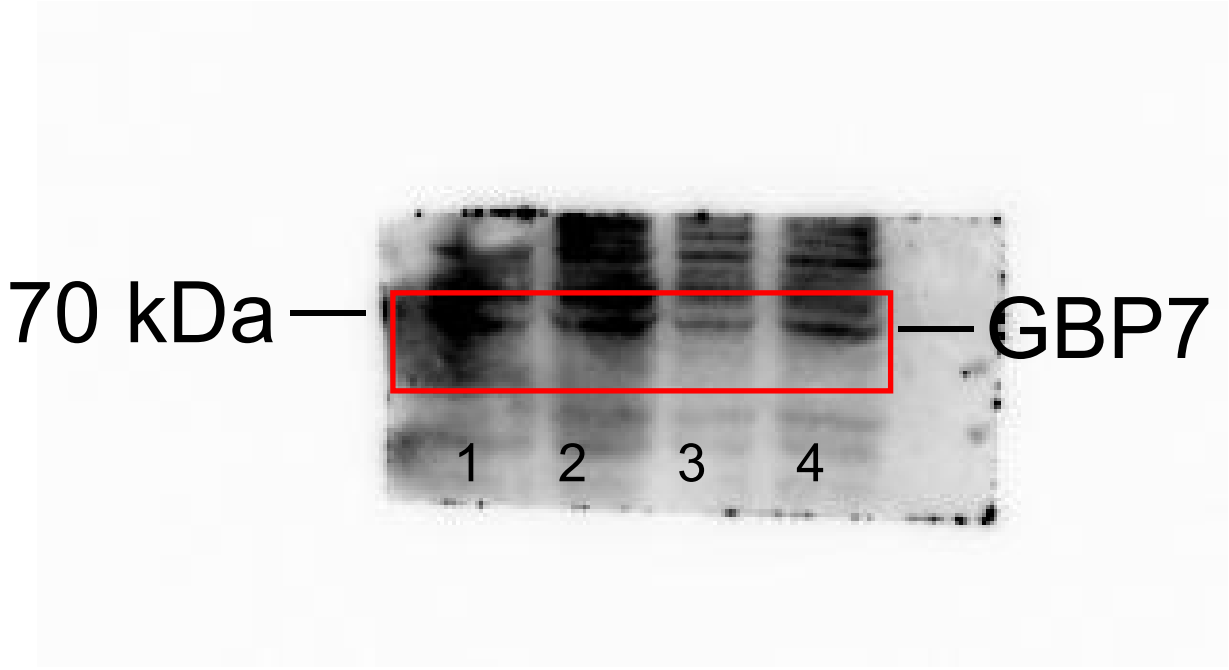


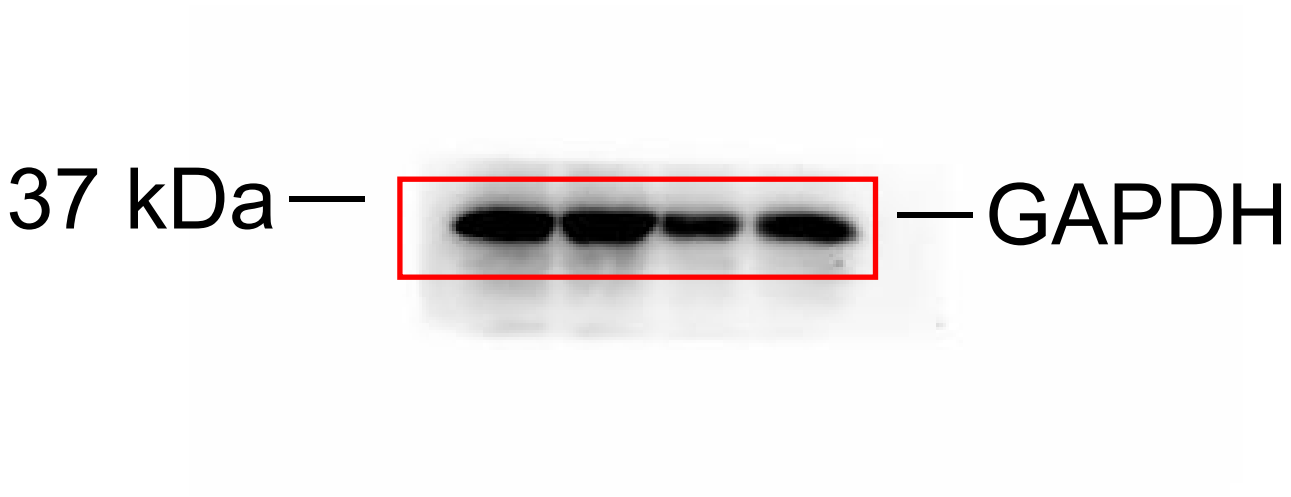

Full and uncropped western blot for S5C Fig, Chemiluminescence (ECL)

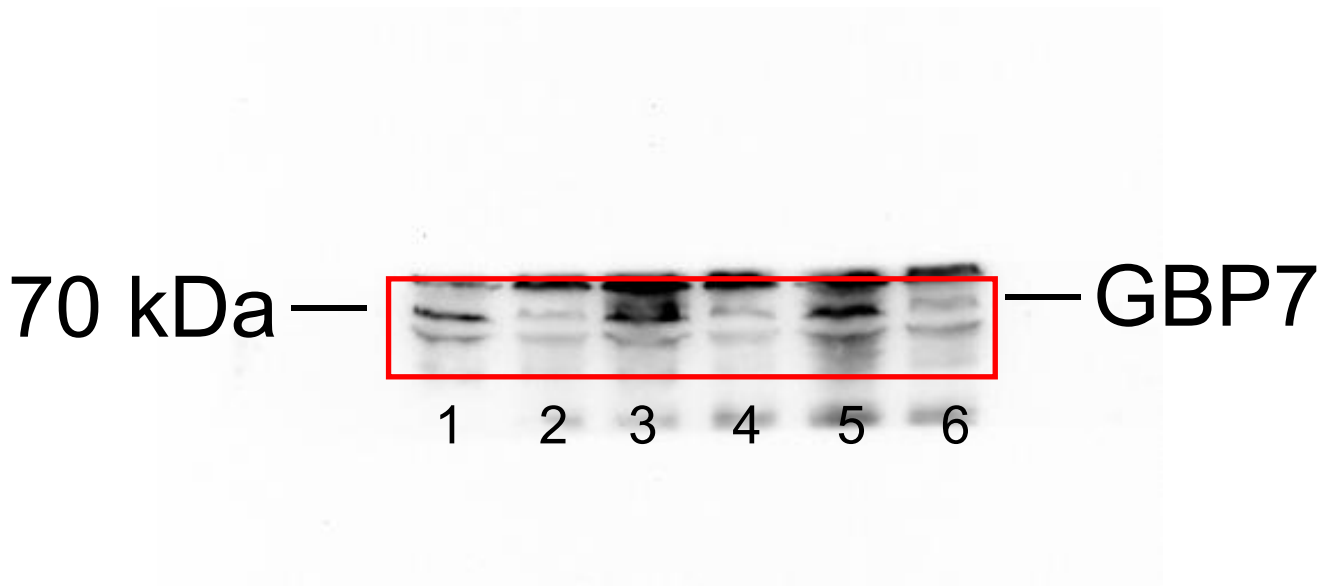


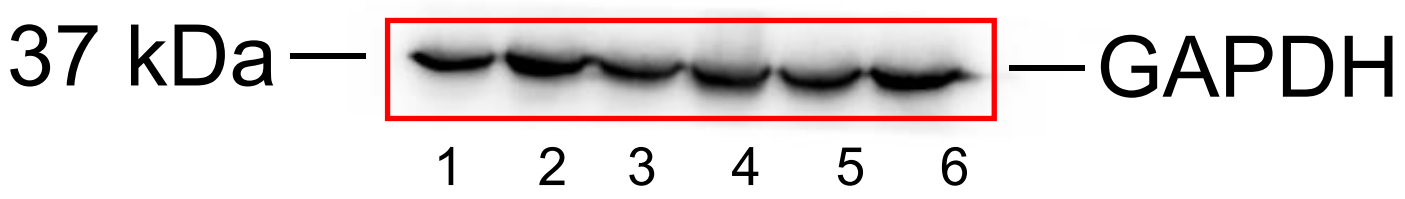

# Full and uncropped western blot for S7C and D Fig, Chemiluminescence (ECL)

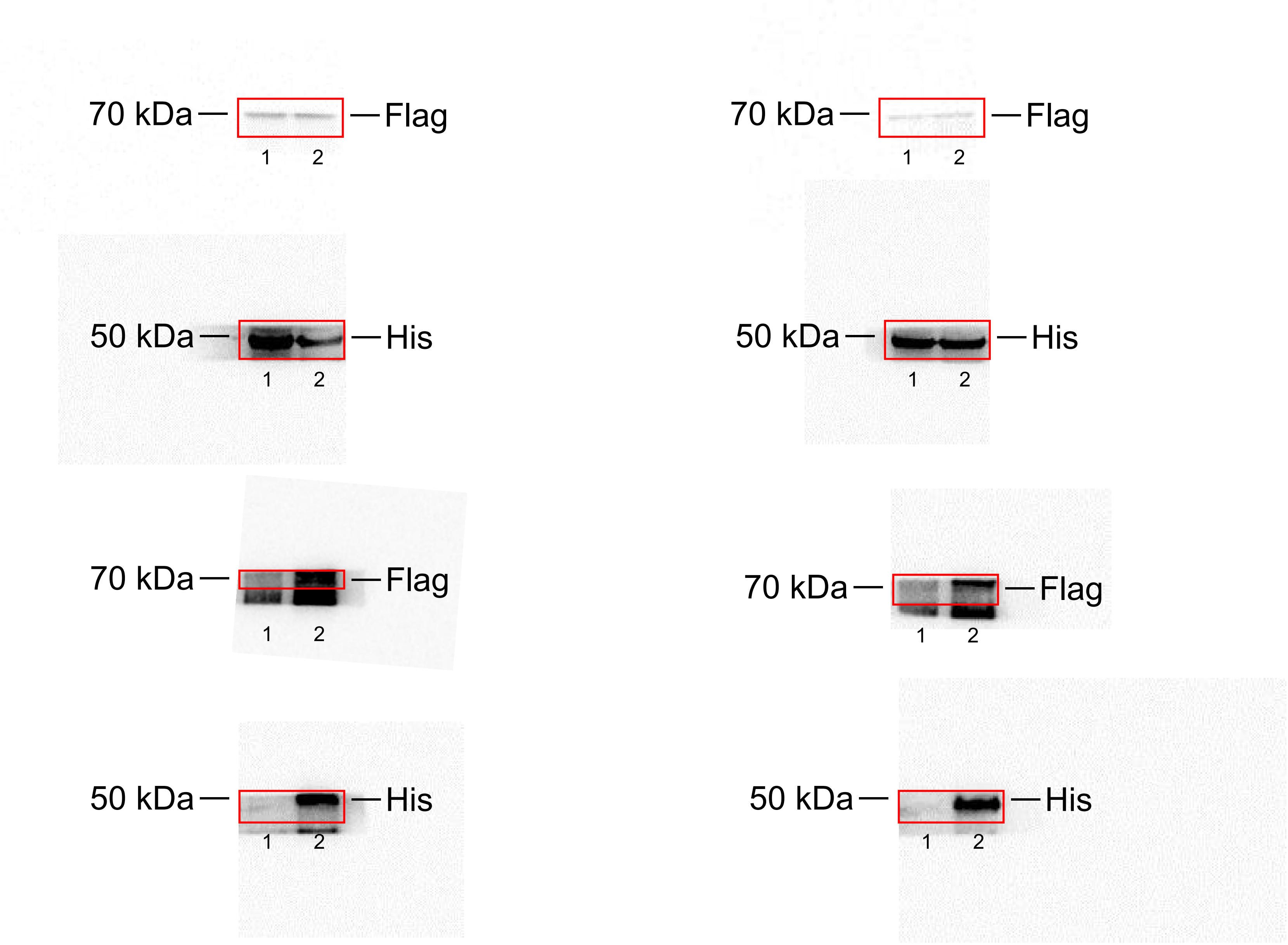

# Full and uncropped western blot for S7I Fig, Chemiluminescence (ECL)

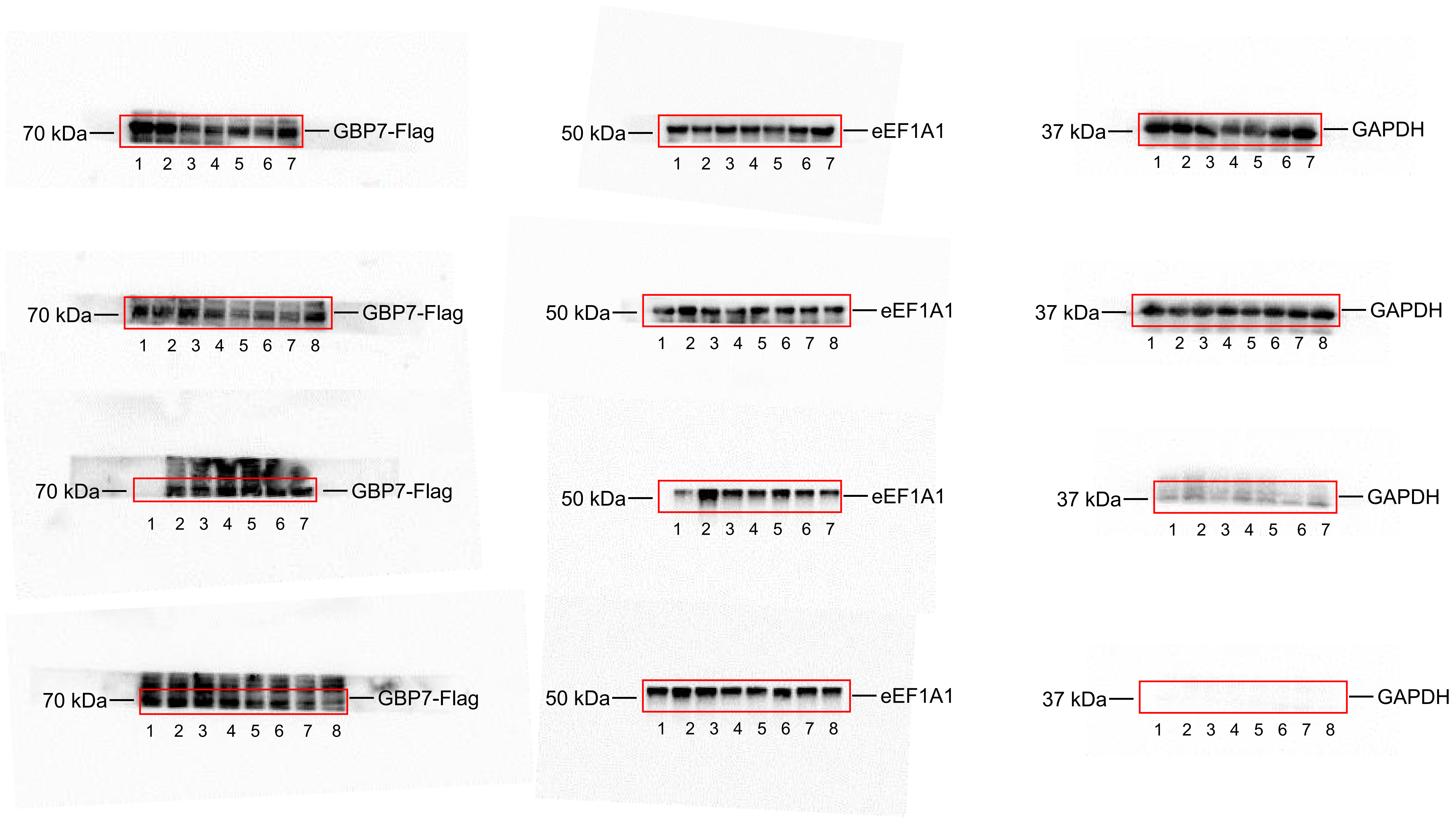

Full and uncropped western blot for S9A Fig, Chemiluminescence (ECL)

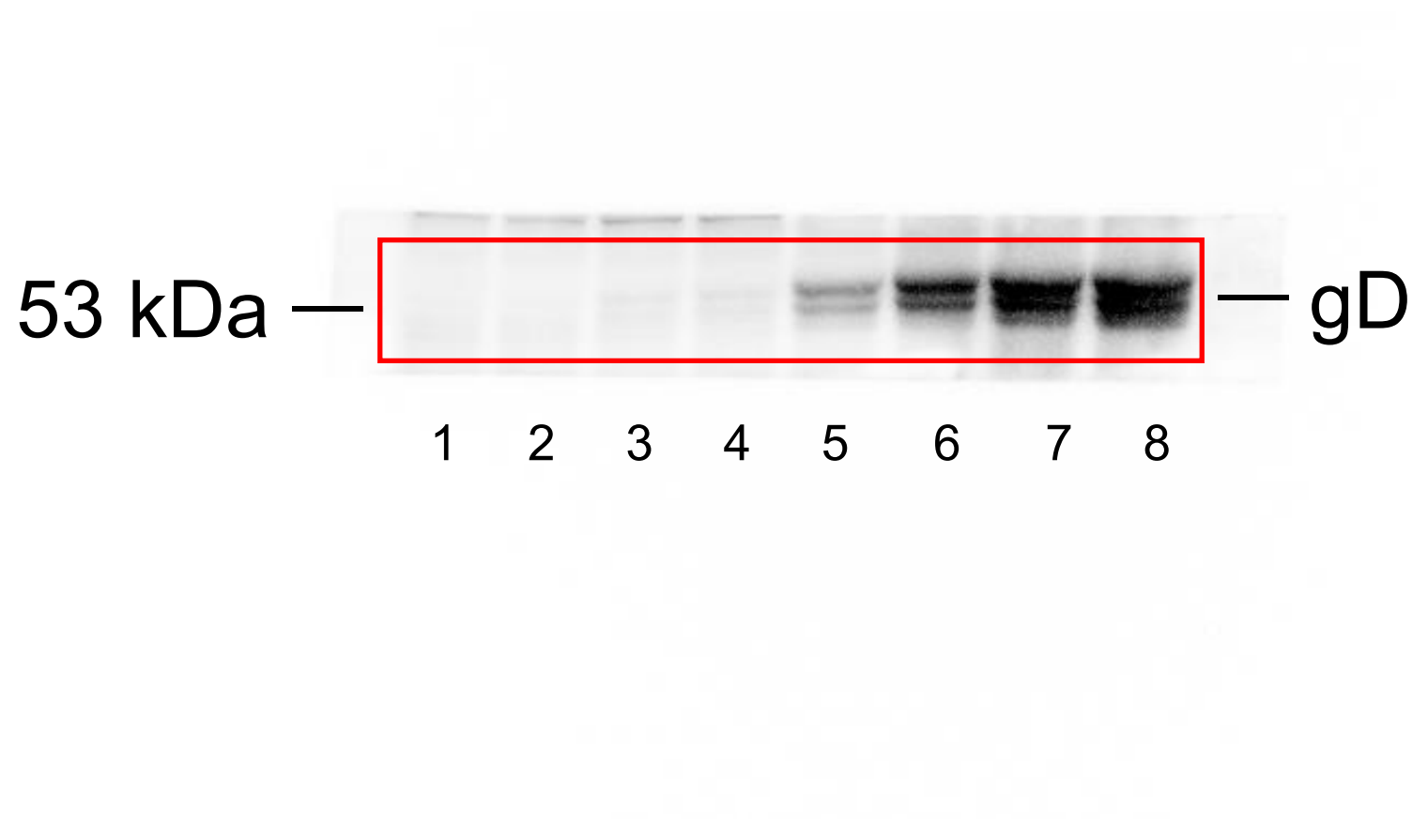


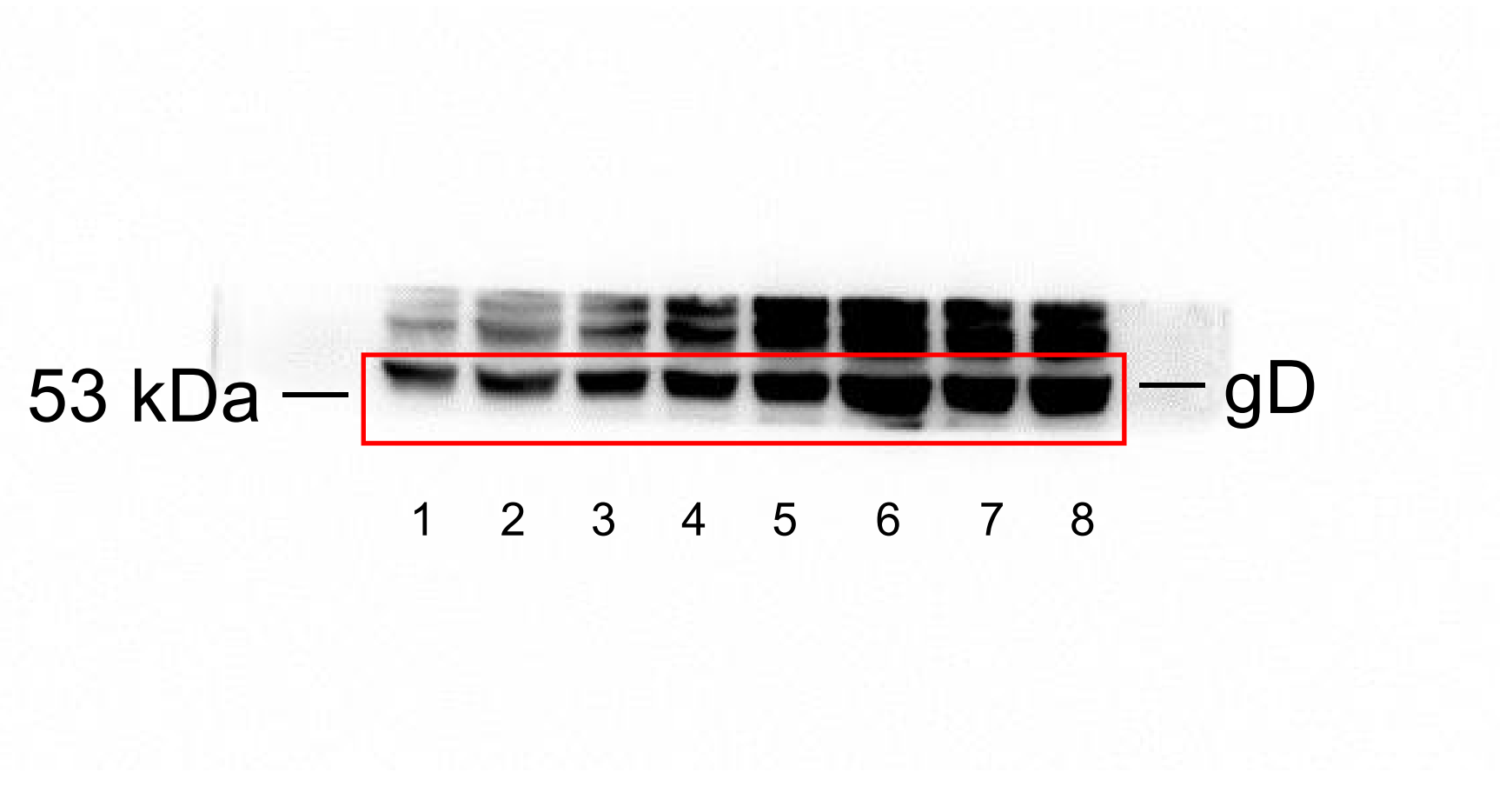


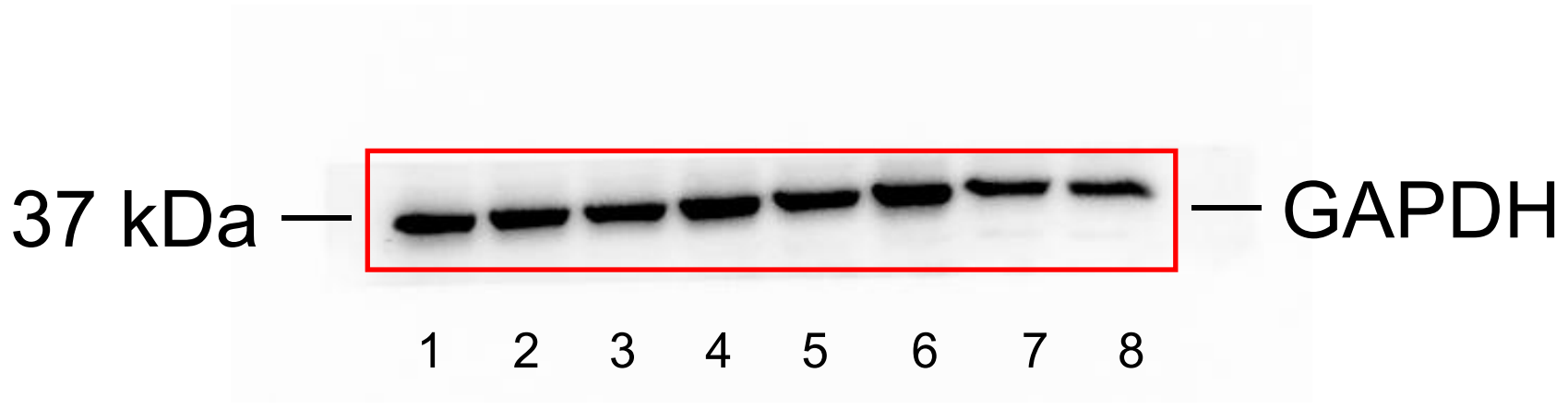


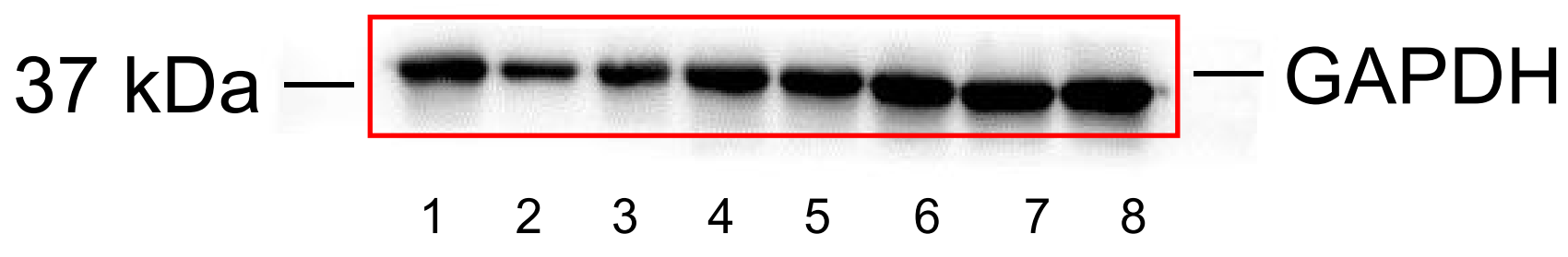

Full and uncropped western blot for S9B Fig, Chemiluminescence (ECL)

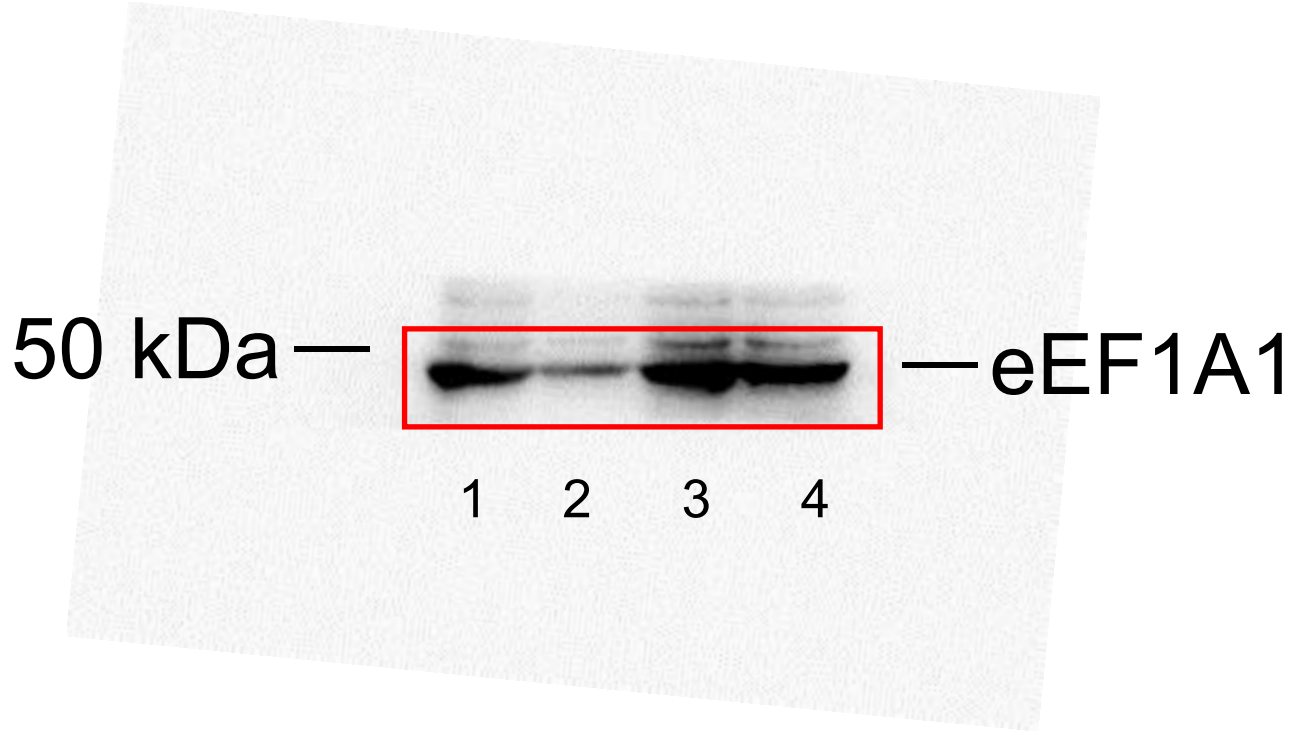


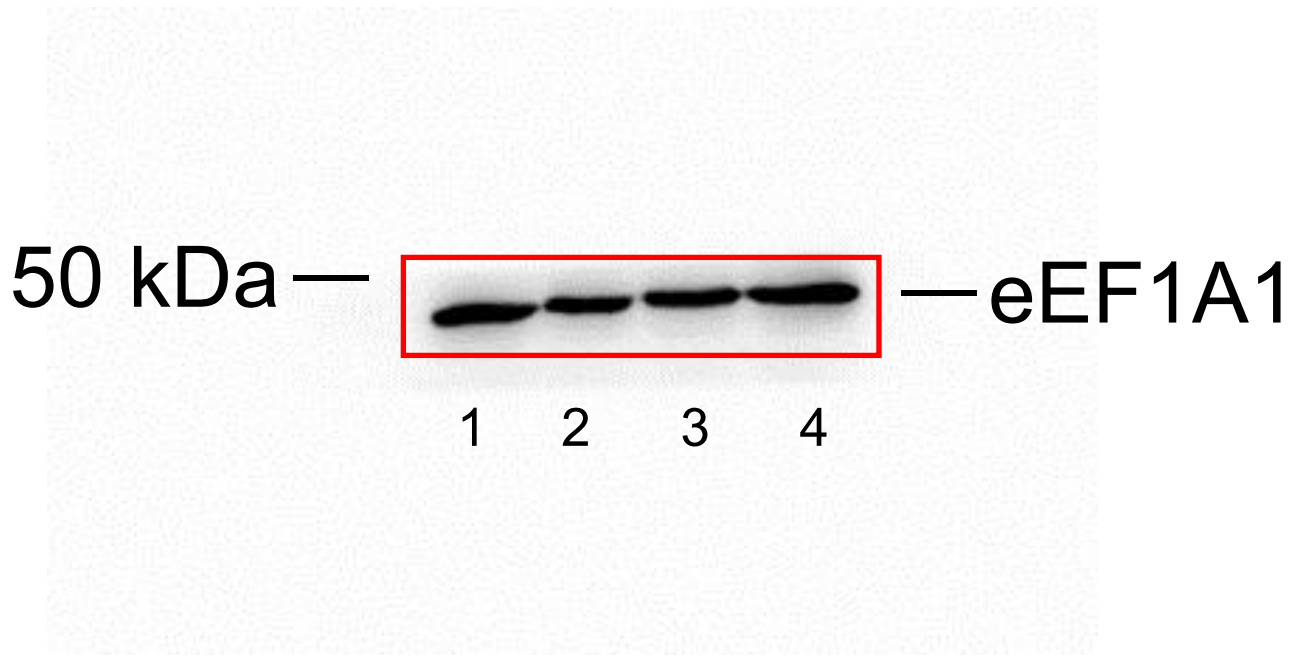

Full and uncropped western blot for S9C Fig, Chemiluminescence (ECL)

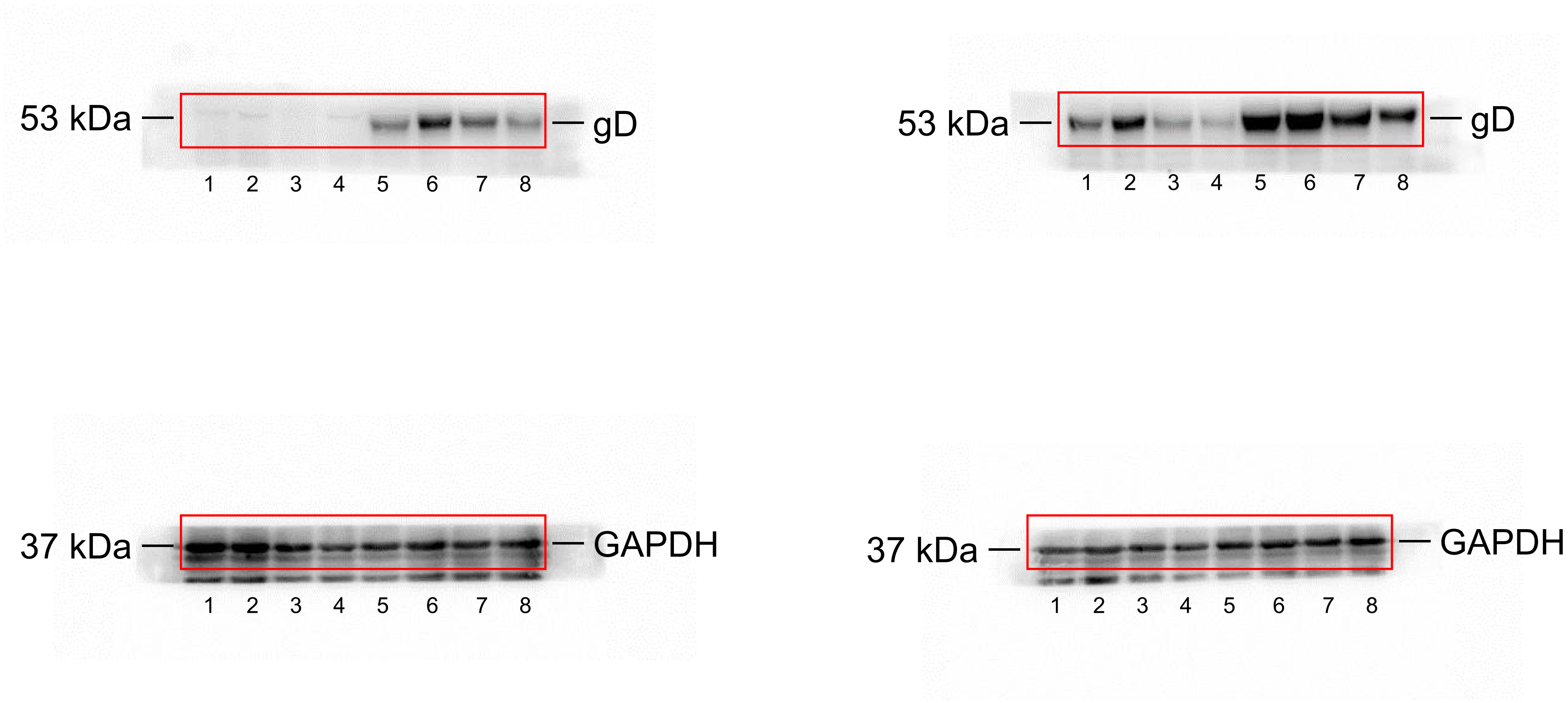

# Full and uncropped western blot for S11 Fig, Chemiluminescence (ECL)

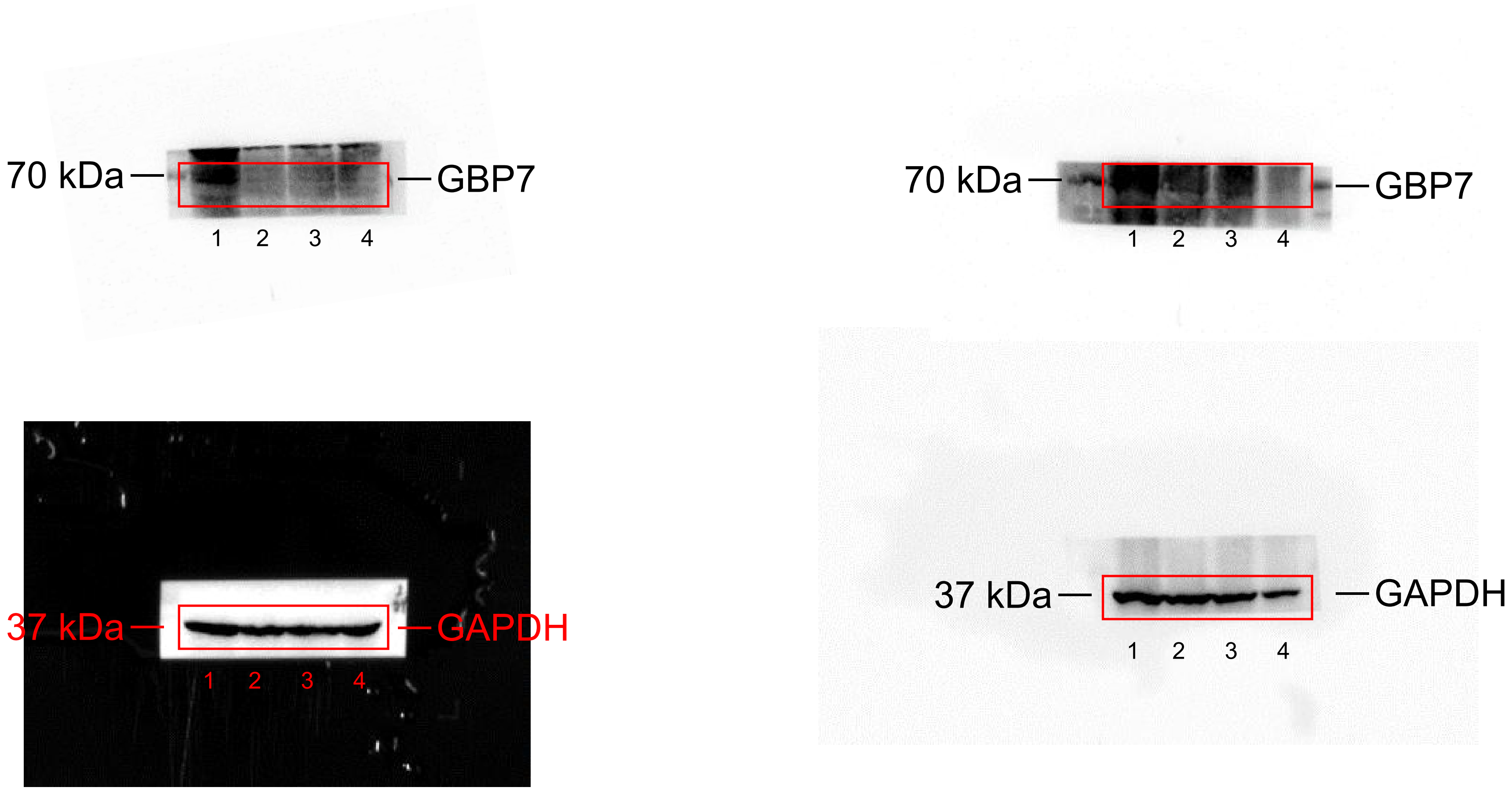

Full and uncropped western blot for S13 Fig, Chemiluminescence (ECL)

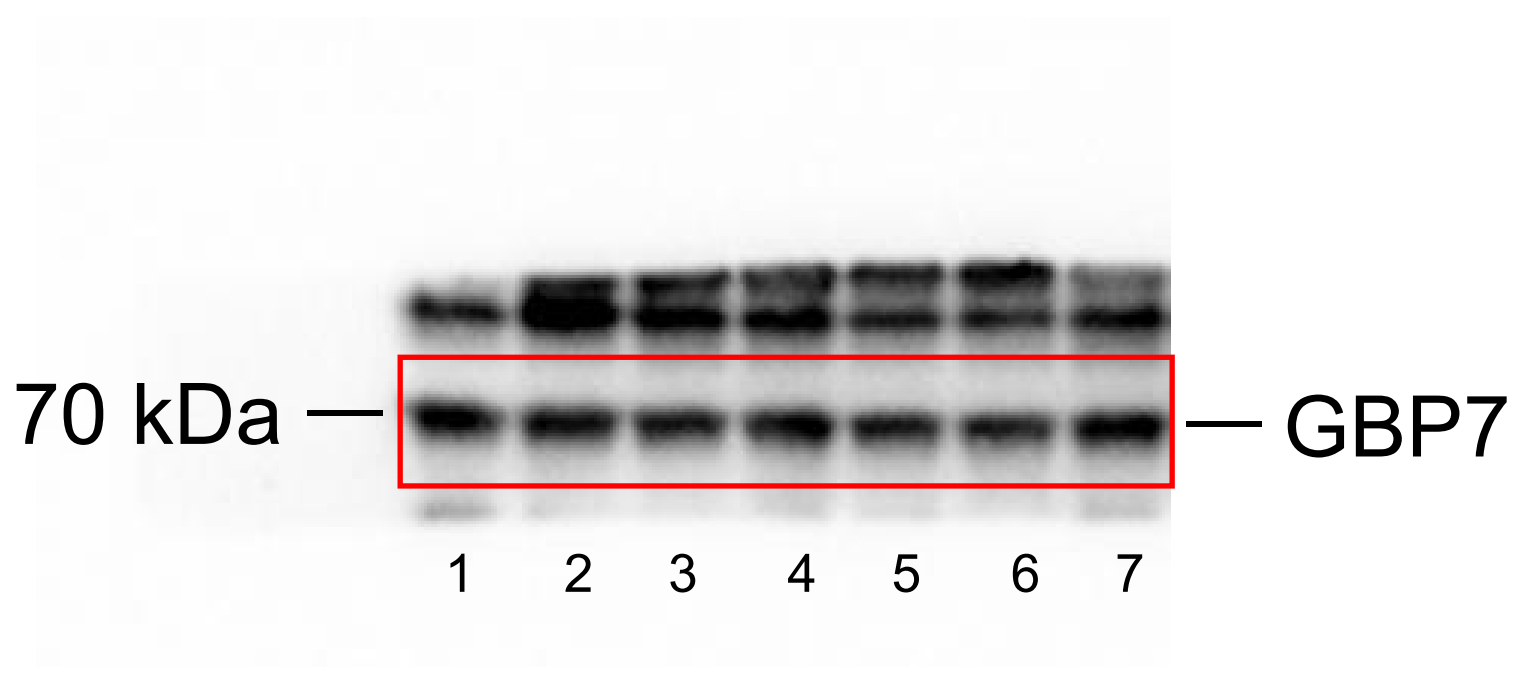


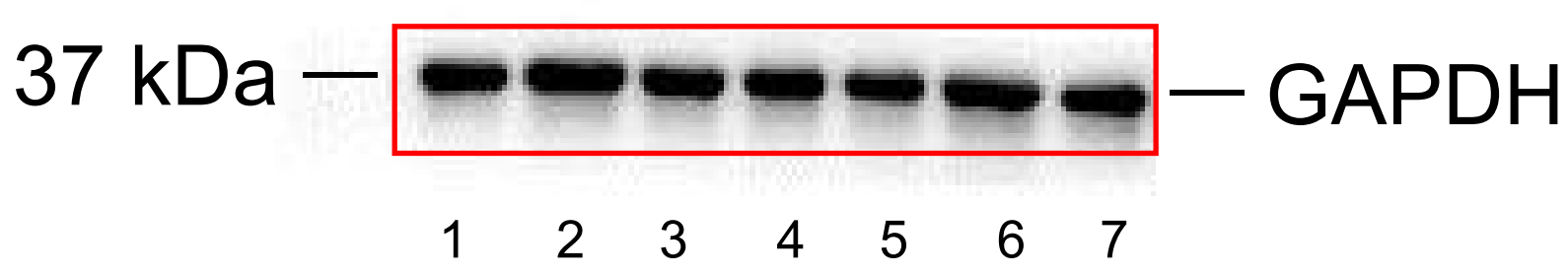

Supplement: Supplementary file 1 [file ppat.1014652.s020.pdf]
