# Supplementary material for: GTPase GBP7 promotes HSV-1 infection by interacting with and activating eEF1A1 to accelerate the protein translation process
Source: PLoS Pathog. 2026 Oct 12;22(10):e1014652. doi: 10.1371/journal.ppat.1014652 (PMC13759050; doi:10.1371/journal.ppat.1014652)
Supplement: Supplementary file 2 [file ppat.1014652.s014.docx]

**Table S1. Peptide-spectrum matches of the interacting proteins with GBP7-Flag from IP-MS (PSM > 1)**

| Accession | Gene Symbol | Coverage [%] | Peptides | PSMs | Unique Peptides | AAs | MW [kDa] | calc. pI | Sequest HT |
| --- | --- | --- | --- | --- | --- | --- | --- | --- | --- |
| Q8N8V2 | GBP7 | 15 | 11 | 15 | 11 | 638 | 72.5 | 5.99 | 36.2 |
| P07437 | TUBB | 10 | 5 | 14 | 3 | 444 | 49.6 | 4.89 | 32.31 |
| P68104 | eEF1A1 | 17 | 8 | 8 | 8 | 462 | 50.1 | 9.01 | 18.58 |
| P48668 | KRT6C | 10 | 7 | 8 | 1 | 564 | 60 | 8 | 16.72 |
| Q13509 | TUBB3 | 7 | 4 | 7 | 2 | 450 | 50.4 | 4.93 | 16.18 |
| Q07065 | CKAP4 | 12 | 5 | 6 | 5 | 602 | 66 | 5.92 | 14.07 |
| P22090 | RPS4Y1 | 16 | 4 | 4 | 4 | 263 | 29.4 | 10.24 | 10.19 |
| P50914 | RPL14 | 9 | 2 | 4 | 2 | 215 | 23.4 | 10.93 | 10.23 |
| P04844 | RPN2 | 4 | 3 | 3 | 3 | 631 | 69.2 | 5.69 | 6.86 |
| O75477 | ERLIN1 | 9 | 3 | 3 | 3 | 348 | 39.1 | 7.87 | 6.52 |
| P18077 | RPL35A | 22 | 3 | 3 | 3 | 110 | 12.5 | 11.06 | 6.37 |
| P16615 | ATP2A2 | 1 | 1 | 3 | 1 | 1042 | 114.7 | 5.34 | 7.91 |
| P83881 | RPL36A | 19 | 3 | 3 | 1 | 106 | 12.4 | 10.58 | 7.31 |
| P08238 | HSP90AB1 | 5 | 3 | 3 | 1 | 724 | 83.2 | 5.03 | 7.23 |
| P52907 | CAPZA1 | 11 | 2 | 2 | 2 | 286 | 32.9 | 5.69 | 5.68 |
| O94905 | ERLIN2 | 6 | 2 | 2 | 2 | 339 | 37.8 | 5.62 | 5.51 |
| Q969U6 | FBXW5 | 4 | 2 | 2 | 2 | 566 | 63.9 | 6.33 | 5.42 |
| P18124 | RPL7 | 10 | 2 | 2 | 2 | 248 | 29.2 | 10.65 | 4.61 |
| P27824 | CANX | 3 | 2 | 2 | 2 | 592 | 67.5 | 4.6 | 4.58 |
| P04843 | RPN1 | 3 | 2 | 2 | 2 | 607 | 68.5 | 6.38 | 4.51 |
| Q14683 | SMC1A | 2 | 2 | 2 | 2 | 1233 | 143.1 | 7.64 | 4.43 |
| Q8N5F7 | NKAP | 7 | 2 | 2 | 2 | 415 | 47.1 | 10.11 | 4.38 |
| Q15149 | PLEC | 0 | 2 | 2 | 2 | 4684 | 531.5 | 5.96 | 4.31 |
| P11940 | PABPC1 | 4 | 2 | 2 | 2 | 636 | 70.6 | 9.5 | 4.29 |

| Accession | Gene Symbol | Coverage [%] | Peptides | PSMs | Unique Peptides | AAs | MW [kDa] | calc. pI | Sequest HT |
| --- | --- | --- | --- | --- | --- | --- | --- | --- | --- |
| P62879 | GNB2 | 6 | 2 | 2 | 2 | 340 | 37.3 | 6 | 4.16 |
| P11172 | UMPS | 4 | 2 | 2 | 2 | 480 | 52.2 | 7.24 | 4.11 |
| Q9Y2L1 | DIS3 | 2 | 2 | 2 | 2 | 958 | 108.9 | 7.14 | 3.98 |
| Q14257 | RCN2 | 5 | 2 | 2 | 2 | 317 | 36.9 | 4.4 | 3.87 |
| P52597 | HNRNPF | 6 | 2 | 2 | 1 | 415 | 45.6 | 5.58 | 5.09 |
| Q9P0L2 | MARK1 | 4 | 2 | 2 | 1 | 795 | 88.9 | 9.36 | 4.54 |
| Q9HCR9 | PDE11A | 1 | 1 | 2 | 1 | 933 | 104.7 | 6.6 | 4.34 |
| Q4VCS5 | AMOT | 1 | 1 | 2 | 1 | 1084 | 118 | 7.64 | 4.25 |
| P14868 | DARS1 | 2 | 1 | 2 | 1 | 501 | 57.1 | 6.55 | 4.06 |
| Q7RTS7 | KRT74 | 4 | 2 | 2 | 1 | 529 | 57.8 | 7.71 | 3.89 |
