# Supplementary material for: GTPase GBP7 promotes HSV-1 infection by interacting with and activating eEF1A1 to accelerate the protein translation process
Source: PLoS Pathog. 2026 Oct 12;22(10):e1014652. doi: 10.1371/journal.ppat.1014652 (PMC13759050; doi:10.1371/journal.ppat.1014652)
Supplement: Supplementary file 3 [file ppat.1014652.s015.docx]

**Table S2. MMGBSA scoring for the molecular docking of compounds with GBP7**

| Compound | MMGBSA (Kcal/mol) | Compound | MMGBSA (Kcal/mol) | Compound | MMGBSA (Kcal/mol) |
| --- | --- | --- | --- | --- | --- |
| T32511 | -72.748665 | T1510 | -60.19236 | T5193 | -53.639954 |
| T38063 | -72.203896 | TQ0208 | -60.080437 | T8244 | -53.624741 |
| T7676 | -71.425171 | T33868 | -59.094978 | T26308 | -53.541065 |
| T67853 | -69.593979 | T4315 | -58.929638 | T5265 | -53.538834 |
| T0430 | -68.903656 | T40187 | -58.593769 | T3S2007 | -53.229694 |
| T17724 | -68.81073 | T9501 | -58.155338 | T10896L | -53.02364 |
| TQ0239 | -68.292633 | T2534 | -57.911263 | T14017 | -52.940216 |
| TP1254 | -67.5905 | TP1832 | -57.048584 | T2454 | -52.863121 |
| T7593 | -67.389786 | T5848 | -57.007431 | T2954 | -52.842155 |
| T21831 | -66.632423 | T5847 | -56.975582 | T31083 | -52.745747 |
| T6829 | -65.920486 | T0055 | -56.479111 | TN1051 | -52.542084 |
| T21640 | -65.327805 | T0749 | -56.377235 | T36935 | -52.230362 |
| T3148 | -63.308361 | T19655 | -56.176659 | T38612 | -52.146671 |
| T77695 | -62.618256 | T20255 | -55.66843 |  |  |
| T14510 | -62.205967 | T77562 | -55.324749 |  |  |
| T18639 | -62.152763 | TP2364L | -54.931602 |  |  |
| T6870 | -61.774117 | T66645 | -54.863758 |  |  |
| T3628 | -61.752693 | T10276 | -54.753319 |  |  |
| T40108 | -61.534676 | T6037 | -54.343494 |  |  |
| T4100 | -61.217728 | T5S1133 | -53.738194 |  |  |
