# Supplementary material for: GTPase GBP7 promotes HSV-1 infection by interacting with and activating eEF1A1 to accelerate the protein translation process
Source: PLoS Pathog. 2026 Oct 12;22(10):e1014652. doi: 10.1371/journal.ppat.1014652 (PMC13759050; doi:10.1371/journal.ppat.1014652)
Supplement: Supplementary file 4 [file ppat.1014652.s016.docx]

**Table S3. SgRNA primer sequence**

|  | Forward primer (5′-3′) | Reverse primer (5′-3′) |
| --- | --- | --- |
| Human GBP7 sgRNA-1 | CACCGACAGTGAAGTCTGAAACCAA | CAAATTGGTTTCAGACTTCACTGTC |
| Human GBP7 sgRNA-2 | CACCGACTTCACTGTGCAGCCCAG | CAAACTGGGCTGCACAGTGAAGTC |
| Human GBP7 sgRNA-3 | CACCGACTTCACTGTGCAGCCCAGA | CAAATCTGGGCTGCACAGTGAAGTC |
