# Supplementary material for: GTPase GBP7 promotes HSV-1 infection by interacting with and activating eEF1A1 to accelerate the protein translation process
Source: PLoS Pathog. 2026 Oct 12;22(10):e1014652. doi: 10.1371/journal.ppat.1014652 (PMC13759050; doi:10.1371/journal.ppat.1014652)
Supplement: Supplementary file 5 [file ppat.1014652.s017.docx]

**Table S4. SiRNA primer sequence**

|  | Sense sequence (5′-3′) | Antisense sequence (5′-3′) |
| --- | --- | --- |
| Human eEF1A1 siRNA-1 | GCAAGUACUAUGUGACUAUTT | AUAGUCACAUAGUACUUGCTT |
| Human eEF1A1 siRNA-2 | CCAAGUGCUAACAUGCCUUTT | AAGGCAUGUUAGCACUUGGTT |
| Human eEF1A1 siRNA-3 | CCGGUAUGGUGGUCACCUUTT | AAGGUGACCACCAUACCGGTT |
