# Supplementary material for: GTPase GBP7 promotes HSV-1 infection by interacting with and activating eEF1A1 to accelerate the protein translation process
Source: PLoS Pathog. 2026 Oct 12;22(10):e1014652. doi: 10.1371/journal.ppat.1014652 (PMC13759050; doi:10.1371/journal.ppat.1014652)
Supplement: Supplementary file 6 [file ppat.1014652.s018.docx]

**Table S5. The source, catalogue number and lot for commercial antibodies**

| Antibodies | Source | Catalogue |
| --- | --- | --- |
| Mouse anti-GAPDH | Fdbio science | CAT#: FD0063 |
| Mouse anti-HSV-1+HSV-2 gD | Abcam | CAT#: ab6507 |
| Rabbit anti-GBP7 | Abcam | CAT#: ab104293 |
| Mouse anti-HSV-1+HSV-2 VP16 | Abcam | CAT#: ab110226 |
| Mouse anti-EF 1α1/2 | SANTA CRUZ BIOTECHNOLOGY | CAT#: sc-377439 |
| Mouse anti-FLAG® M2 | Sigma-Aldrich | CAT#: F1804 |
| Rabbit anti-6*his, His tag | Proteintech | CAT#:10001-0-AP |
| Goat Anti-Mouse IgG H&L (Alexa Fluor 488) | Abcam | CAT#: ab150113 |
| Goat anti-Rabbit IgG H&L (Alexa Fluor 594) | Abcam | CAT#: ab150080 |
| Normal Rabbit IgG | Cell Signaling Technology | CAT#: 2729S |
| Normal Mouse IgG | Cell Signaling Technology | CAT#: 68860L |
