# Supplementary material for: GTPase GBP7 promotes HSV-1 infection by interacting with and activating eEF1A1 to accelerate the protein translation process
Source: PLoS Pathog. 2026 Oct 12;22(10):e1014652. doi: 10.1371/journal.ppat.1014652 (PMC13759050; doi:10.1371/journal.ppat.1014652)
Supplement: Supplementary file 7 [file ppat.1014652.s019.docx]

**Table S6. Primers used for quantitative real time PCR analysis**

|  | Forward primer (5′-3′) | Reverse primer (5′-3′) |
| --- | --- | --- |
| Human GAPDH | GAACGGGAAGCTCACTGG | GCCTGCTTCACCACCTTCT |
| Human GBP1 | AGGAGTTCCTTCAAAGATGTGGA | GCAACTGGACCCTGTCGTT |
| Human GBP2 | CTATCTGCAATTACGCAGCCT | TGTTCTGGCTTCTTGGGATGA |
| Human GBP3 | ATTCCCTGAAGCTAACGCAAG | GGGCAGATCGAAGACAAAACATT |
| Human GBP4 | ATGGGTGAGAGAACTCTTCACG | TGCGGTATAGCCCTACAATGG |
| Human GBP5 | CCATGTGCCTCATCGAGAACT | ACAGGTTGCGTAATGGCAGAC |
| Human GBP6 | ATGGAATCTGGACCCAAAATGTT | GCTGGTTCACCAATAGCTGCT |
| Human GBP7 | GCTGCACTACGTGACTGAG | TCCCTGGGCTTGTTAGAATTTTG |
| Mouse GBP7 | AACTGAGGGTGAACTCCAAAGC | GTTTCAGACCTAACTGTGGTGC |
| VP16 | TTGACTGCCTCTGTTGCGAC | ATGTGGTTTAGCTCCCGCAG |
| ICP8 | CGTGGGGGCCATGGTATTTA | TTTGCTGTGGTCCTTTGGGT |
| UL30 | CCACGAGATCTCTGCGAGCG | AGCGCACCGACGTGTACTAC |
| IAV-NP | TCAAGTGAGAGAGAGCCGGA | TGAGTGCAGACCGTGCTAAA |
| VSV-P | CATAGTCTACAGAGAATATTTGACTCTCGCC | GCTTGATATCATGGATAATCTCACAAAAGTTCG |
| ZIKA-E | GGTGGGACTTGGGTTGATGT | ATGTCACCAGGCTCCCTTTG |
| DENV-E | GAGGGGAGCGAAGAGAATGG | GCCCCATAGATTGCTCCGAA |
| Human eEF1A1 | TGTCGTCATTGGACACGTAGA | ACGCTCAGCTTTCAGTTTATCC |
