# Supplementary material for: GTPase GBP7 promotes HSV-1 infection by interacting with and activating eEF1A1 to accelerate the protein translation process
Source: PLoS Pathog. 2026 Oct 12;22(10):e1014652. doi: 10.1371/journal.ppat.1014652 (PMC13759050; doi:10.1371/journal.ppat.1014652)
Supplement: Supplementary file 9 [file ppat.1014652.s022.docx]

**Graphical Abstract. Proposed model of GBP7-mediated enhancement of viral protein translation and its inhibition by SAH.**

This schematic illustrates the proposed mechanism by which GBP7 promotes viral protein translation and the inhibitory effect of SAH on this process. Following viral invasion, activation of host immune pathways induces IFN‑γ expression, which subsequently upregulates GBP7. Within the host cell, the virus undergoes uncoating, nuclear import, replication, and transcription, culminating in the production of progeny virions. GBP7 interacts with eukaryotic translation elongation factor 1α1 (eEF1A1) and, through its GTPase activity, facilitates viral protein translation. The small‑molecule compound SAH specifically targets GBP7 by suppressing both its GTPase activity and protein expression, thereby disrupting the GBP7–eEF1A1 interaction and consequently inhibiting viral protein synthesis, ultimately reducing the yield of progeny virus.
