# Supplementary material for: GTPase GBP7 promotes HSV-1 infection by interacting with and activating eEF1A1 to accelerate the protein translation process
Source: PLoS Pathog. 2026 Oct 12;22(10):e1014652. doi: 10.1371/journal.ppat.1014652 (PMC13759050; doi:10.1371/journal.ppat.1014652)
Supplement: Supplementary file 10 [file ppat.1014652.s024.docx]

**Responses to Reviewers**

（Manuscript Number: PPATHOGENS-D-26-00828）

We thank the reviewers for their insightful comments and critiques. Below is our point-by-point response to the reviewers’ critiques. We have revised the manuscript according to the suggestions and comments. The added or changed sentences that were highlighted in red for convenience of reviewers and editors to review.

**Reviewers' Comments:**

Reviewer's Responses to Questions

Part I - Summary

Please use this section to discuss strengths/weaknesses of study, novelty/significance, general execution and scholarship.

Reviewer #1: This manuscript by Wang et al. delineates an unexpected proviral function of guanylate-binding protein 7 (GBP7). GBP7 promotes HSV-1 infection by interacting with and activating eEF1A1 to accelerate the protein translation process. This work addresses a critical gap in the functional characterization of the GBP family, and uncovers a novel mechanism by which HSV-1 hijacks the host innate immune response to support its replication. The identification of GBP7 as a host-directed antiviral target has important translational value for overcoming drug resistance in HSV-1 infection. It is of broad interest to the fields of virology, innate immunity and antiviral drug development, pending resolution of the following concerns.

Reviewer #2: This study systematically investigates the function and molecular mechanism of GBP7 during herpes simplex virus type 1 (HSV-1) infection using a comprehensive, multi‑level experimental design. The work reports for the first time that GBP7, a canonical interferon‑inducible GTPase, does not function as an antiviral factor but instead significantly promotes HSV-1 replication and infection. The authors further elucidate a novel regulatory axis: GBP7 directly binds eEF1A1, enhances GTP hydrolysis, and accelerates viral protein translation elongation. Additionally, virtual screening identifies SAH as a GBP7‑targeted inhibitor with potent anti‑HSV-1 and broad‑spectrum antiviral activity in cells and mice. Overall, this study features high novelty, rigorous logic, sufficient experimental evidence, and standardized methodology. It not only expands understanding of the GBP protein family but also provides a novel target and candidate molecule for host‑directed broad‑spectrum antiviral drug development, with important scientific significance and translational potential. However, there are deficiencies in aspects such as the rigor of experimental design, the depth of mechanism verification, the accuracy of result interpretation, and the expansion of significance, which need to be further improved to enhance the scientificity and influence of the research.

Reviewer #3: In this manuscript, Wang and collogues perform a detailed characterization of the role of the GBP7 GTPase in the activation of eEF1A1, showing how this interaction favors HSV-1 viral production. However, critical controls and clearer explanations are needed to improve the robustness of the data provided, as suggested in the key points of this revision. Findings are relevant as they reveal for the first time how GBP7 impacts the activity of eEF1A on protein translation, and how the modulation of GBP7 can lead to a different approach to develop broad spectrum therapies. This indeed aligns with prior evidence on eEF1A blockade, for which a broad-spectrum antiviral activity has been recently identified, what adds to the prior well-known activity of drugs targeting eEF1A that have shown antiviral activity in vitro and in vivo, being safe in several clinical trials. This information is currently missing from the manuscript and could help to strengthen the findings of this paper, contextualizing several assumptions of the authors in the discussion section, where there are several claims that are not sustained by the current available evidence.

**Response:** We thank the reviewers for their overall comments and concerns about our study.

**Main comments to the authors:**

1. In the introduction (lines 59-61), the authors state that eEF1A is a host factor required for the replication of different viruses, but they cite reviews, when there are primary references showing how antivirals targeting eEF1A inhibit the replication of multiple viruses, including HSV-1, which are the following:

*Molina Molina E, et al. Targeting eEF1A reprograms translation and uncovers broad-spectrum antivirals against cap or m6A protein synthesis routes. Nat Commun. 2025 Feb 7;16(1):1087. doi: 10.1038/s41467-025-56151-y. PMID: 39920115; PMCID: PMC11805953.

*Albericio G, Rodríguez-Martín D, Avilés P, Cuevas C, Guillén-Navarro MJ, Noriega MA, Flores S, Sánchez-Cordón PJ, Astorgano D, Pérez P, Esteban M, García-Arriaza J. Functional characteristics of plitidepsin as an antiviral treatment against monkeypox virus infection. Antiviral Res. 2025 Sep;241:106238. doi: 10.1016/j.antiviral.2025.106238. Epub 2025 Jul 15. PMID: 40675496.

**Response:** We sincerely thank the reviewer for this rigorous suggestions. We have replaced the review citations with the original research articles (Molina Molina et al., Nat Commun, 2025; Albericio et al., Antiviral Res, 2025). These studies provide direct evidence that pharmacological blockade of eEF1A suppresses the replication of multiple viruses, including HSV-1, thereby firmly supporting the statement that eEF1A is a host factor required for viral replication. **(Revised lines 61-66)**

In line 64 and 65, it is also stated that inhibition of eEF1A is a promising avenue for antiviral efficacy, but no references are given, when clinical trials have already shown safety and efficacy of molecules targeting this host factor:

*Varona, J.F., et al., 2022. Preclinical and randomized phase I studies of plitidepsin in adults hospitalized with COVID-19. Life Sci. Alliance 5, e202101200. https://doi.org/10.26508/lsa.202101200

*Landete, P., et al, J.-F., 2024. A phase III randomized controlled trial of plitidepsin, a marine derived compound, in hospitalized adults with moderate COVID-19. Clin Infect Dis ciae227. https://doi.org/10.1093/cid/ciae227

Finally, in lines 78-81 the authors mention the role of GBP7-eEFA1 axis as a broad spectrum antiviral approach, but this was already known for plitidepsin, a drug blocking eEFA1 that inhibits viral replication in vitro and in vivo of several viruses (please, see the above mentioned references) and has shown safety in clinical trials (see the above mentioned references).

**Response:** Thanks for the reviewer's suggestion; we have reviewed the aforementioned references. In **Revised lines 67-60**, we now explicitly cite the clinical studies by Varona et al. (Life Sci Alliance, 2022) and Landete et al. (Clin Infect Dis, 2024), which document the safety of the eEF1A inhibitor plitidepsin in Phase I and Phase III randomized controlled trials for COVID-19, providing robust support for the translational potential of targeting targeting eEF1A and its regulatory pathways. Further, we have carefully revised the text to avoid implication that a host-directed, broad-spectrum antiviral strategy targeting eEF1A is entirely new.

2. The authors plot and compare viral and cellular mRNA transcripts in a linear scale in most of the figures of this manuscript, and they say that they refer it to GDPAH. This analysis can be biased, as blocking protein translation leads to a cytostatic effect that decreases cell division, and therefore, if no controls are provided to show that the same number of cells are added, interpretations can be misleading. Moreover, I am having a hard time to understand why an inhibitor of protein translation is having an impact on viral mRNA 24h pi, when most likely no new infections are taking place and similar viral mRNAs would be found in the cell. This is precisely what the authors see in the animal model in Fig 3G, where no change in RNA is detected. Indeed, if GBP7 is overexpressed between 24 to 48 hpi (Fig 1) and upon knockdown it can decrease viral mRNA 24 h pi (Fig 2), how do the authors explain that in the murine KO model, there is no decrease in viral mRNA 3 dpi (Fig. 3G) and no differences in viral infectivity measured as viral titers (Fig. 3G)?

While protein quantification experiments are clear to me and support the pathway involving GBP7 and eEF1A in boosting protein translation, experiments showing RNA levels are confusing, so the authors should focus on the effects observed at protein level, especially at short times post infection.

**Response:** We greatly appreciate this insightful comment, which prompted us to critically re-evaluate our mRNA-level data and its interpretation.

Reconsideration of mRNA quantification: We agree that relying solely on GAPDH-standardized mRNA levels may introduce bias when the cell proliferation status changes. In fact, we did not use protein translation inhibitors but instead altered the level of GBP7 through genetic manipulation (overexpression, knockdown, or knockout). We carefully evaluated cell proliferation. There was no significant difference in proliferation rate and viability between GBP7-KO cells and control cells (**Revised S3 Fig**), thus ruling out the bias in cell number in mRNA detection. We emphasize in the revised manuscript that the primary and most direct consequence of the GBP7–eEF1A1 axis is the modulation of protein translation efficiency. The observed reduction in viral mRNA in our in vitro experiments is interpreted as a secondary effect. As demonstrated by White et al. ([Plitidepsin has potent preclinical efficacy against SARS-CoV-2 by targeting the host protein eEF1A. *Science*. 2021;371(6532):926-931.](https://www.science.org/doi/10.1126/science.abf4058)), the eEF1A inhibitor plitidepsin profoundly suppresses viral protein synthesis, and the ensuing shortage of essential viral replication proteins consequently leads to a reduction in viral RNA levels over the course of SARS-CoV-2 infection. Our in vitro data are consistent with this cascade.

3. Do GBP7-KO cell lines have lower viability or cell division rates as compared to controls transduced with empty vectors? And is it similar to the viability and cell division rates observed for eEF1A KO cells, in Fig S6? eEF1A inhibitors are known to display a cytostatic effect that has been very useful in the cancer field, so this should be controlled for too, as this could explain decreases in mRNA.

**Response:** We thank the reviewer for the suggestions. We assessed cell viability using CCK-8 assays (**Revised S3 Fig**), GBP7-KO cells exhibited no significant difference in viability or proliferation rate compared to wild-type cells.

4. Authors state that the complex formed by GBP7 and eEF1A is increased upon infection (4E), but this is not quantified and no colocalization data is provided.

**Response:** We thank the reviewer for the suggestions. The co‑localization results, presented in Fig. 4E, showed that the fluorescence signals of GBP7 and eEF1A1 consistently exhibited substantial overlap. Furthermore, co‑immunoprecipitation (Co‑IP) assays (Fig. 4F) revealed that when GBP7 was immunoprecipitated from HSV‑1‑infected cells, the levels of co‑precipitated eEF1A1 were increased. Similarly, when eEF1A1 was immunoprecipitated, higher amounts of interacting GBP7 were detected compared with those in uninfected cells. We therefore conclude that the GBP7–eEF1A1 complex is augmented following HSV‑1 infection.

5. In the antiviral screening, there is no calculation of the toxicity (CC50) of SAH, via MTT, cell titer glo or other method. No IC_50_ is provided either. In addition, whether blocking GBP7 has a cytostatic effect is not analyzed. Again, from a mechanistic point of view, it is not clear to me why the mRNA of HSV VP16 is decreased 24h post infection (Fig 6D). If we assume that GBP7 inhibition is blocking viral translation via inactivation of eEF1A, it should affect viral protein synthesis, not viral mRNA, as previously observed in the KO animal model, where differences were detected 5 dpi. Assessing protein quantification will provide more robust evidence. In addition, a comparison with known inhibitors that block eEF1A, such as plitidepsin, would be helpful to confirm these findings.

**Response:** We thank the reviewer for the suggestions. We have now provided the IC_50_ of SAH. (**Revised Fig 6G**), and blockade of GBP7 did not significantly affect cell proliferation efficiency (**Revised S3 Fig**). We previously assessed the cytotoxicity of SAH in A549 cells, and have now extended these measurements to include Hela and Vero cells (**Revised** **S12 Fig**). Owing to solubility limitations, the maximum concentration of SAH examined was 200 μM. The primary mechanism by which GBP7 inhibition suppresses viral replication is through blockade of viral translation. Upon GBP7 knockout, viral protein levels, mRNA levels, and progeny virus titers were all reduced at the same time points post‑infection. Although the differences were modest at early times, the viral mRNA levels at the 24 h time point showed a statistically significant difference (Fig 2).

6. In lines 239-241, authors state that SAH not only downregulates the expression of GBP7 protein in cells but also impairs GTPs activity. However, if infection increases GBP7 and SAH blocks infection, it is expected that GBP7 would be expressed to a lower extent, but not due to a direct action of SAH. Most likely, dugs like ACV would lead to the same effect. This should be clarified.

**Response:** We thank the reviewer for the valuable suggestion. Our previous results demonstrated that SAH downregulated GBP7 protein levels even in the absence of viral infection ((**Revised Fig 6J**). In this revision, we have now included the effects of ACV on GBP7 expression under the same conditions ((**Revised S13 Fig**). These results indicate that SAH directly suppresses GBP7 expression even in the absence of infection, whereas ACV does not exert such an effect.

7. If animals in figure 7 were followed over time, why do authors only show titration and VP16 mRNA levels at one timepoint? What is the kinetics of SAH viral inhibition, measured by protein quantification? Given that the best response is obtained using 200 mg/Kg, which is a very high drug dose, what are the chances to move the use of this antiviral further for clinical use? This should be discussed as a limitation.

**Response:** This should be discussed as a limitation. We acknowledge that the 200 mg/kg dose used in our animal study is relatively high and represents a limitation for direct clinical translation. However, we would like to emphasize that the primary focus of the present study is to validate GBP7 as a bona fide antiviral host target and to elucidate the underlying mechanism of action, rather than to establish SAH as a final clinical candidate. The in vivo efficacy data, though obtained at a higher dose, serve as proof‑of‑concept evidence that pharmacological inhibition of GBP7 effectively suppresses HSV‑1 replication in a physiological setting.

8. Broad spectrum activity of SAH is only based on mRNA quantification 24h post infection without any control for cytotoxicity and cytostatic effects of the drug. Additional evidence, such as measuring the impact on viral protein production, which is the expected MoA of SAH when blocking GBP7, will be needed to demonstrate the MoA. Moreover, a side-by-side comparison with plitidepsin, that blocks eEF1A will reinforce this MoA. Complementary assays to show the impact on viral replication, including CPE measurement, will be needed to show the broad-spectrum activity of SHA beyond RNA levels. In addition, calculation of IC50 and CC50 for SAH in each viral model should be provided.

**Response:** In the revised manuscript, we have performed additional Western blotting analyses to confirm that SAH treatment inhibits viral protein expression across multiple viruses, including VSV, IAV, DENV, and ZIKV, which supports the initial exploration of SAH and GBP7 as potential broad‑spectrum antiviral candidates (**Revised Fig 8D**). Regarding the proposed comparison with plitidepsin, we respectfully note that plitidepsin targets eEF1A directly, whereas SAH acts through GBP7—a distinct upstream mechanism. Given that our study focuses primarily on validating GBP7 as a host antiviral target and exploring the proof‑of‑concept potential of SAH, we consider that a side‑by‑side comparison with plitidepsin would not directly contribute to addressing the core scientific questions of this work.We also acknowledge that the broad‑spectrum antiviral activity of SAH presented here is at a preliminary, in vitro stage, intended to support the feasibility of targeting GBP7, rather than to promote SAH as a clinical candidate. Therefore, we have not pursued extensive profiling of compound activity and toxicity across multiple levels in this study. We hope that the additional data we have provided—including viral protein and cytotoxicity—adequately address the reviewer’s concerns and further support the mechanistic conclusions of our study.

9. In the discussion section, authors state “However, the translation elongation process is indispensable for intracellular protein production. Targeting eEF1A1 may affect the normal vital activities of cells and even lead to cell death and, potentially, the death of individuals”. This statement completely ignores current evidence, as several studies have confirmed that targeting eEF1A with plitidepsin does not affect cell viability at the nM concentrations where antiviral effect is achieved. This is because protein translation is not completely shut down, as alternative m6A protein translation routes are not affected by blocking eEF1A and cells can synthesize cellular proteins in the presence of plitidepsin, as shown in:

*Molina Molina E, et al. Targeting eEF1A reprograms translation and uncovers broad-spectrum antivirals against cap or m6A protein synthesis routes. Nat Commun. 2025 Feb 7;16(1):1087. doi: 10.1038/s41467-025-56151-y. PMID: 39920115; PMCID: PMC11805953.

There are also several clinical trials showing the safety of plitidepsin in hospitalized COVID-19 individuals, where no death was reported, as published in:

*Varona, J.F., et al., 2022. Preclinical and randomized phase I studies of plitidepsin in adults hospitalized with COVID-19. Life Sci. Alliance 5, e202101200. https://doi.org/10.26508/lsa.202101200

*Landete, P., et al, J.-F., 2024. A phase III randomized controlled trial of plitidepsin, a marine derived compound, in hospitalized adults with moderate COVID-19. Clin Infect Dis ciae227. https://doi.org/10.1093/cid/ciae227

Moreover, comments such as “Considering the challenges associated with directly targeting eEF1A1” in line 321 are rather confusing as the authors propose to target this factor indirectly by blocking GBP7. Thus, to correctly interpret their data, they should use the paralelism of the observations they found here (broad spectrum activity by indirect blocking of eEF1A via GBP7 inhibition) with those reported earlier by directly targeting eEF1A.

**Minor comments:**

-Please review English style in lines 26-28

**Response:** We sincerely thank the Reviewer for this important comment, which prompted us to refine our discussion of eEF1A1 targeting and to better distinguish complete loss-of-function from pharmacological modulation. We fully agree that the original statement was overly broad and did not account for the evidence that partial, low-dose inhibition of eEF1A1 can achieve antiviral effects without compromising cell viability or organismal safety. We have now revised the manuscript to clearly differentiate scenarios. Genetic ablation or acute depletion of eEF1A1 is indeed incompatible with cell survival.With respect to the statement about “challenges associated with directly targeting eEF1A1,” our intention was to highlight the difficulty of developing direct eEF1A1 inhibitors that are sufficiently selective to avoid on-target toxicity. We have rephrased this passage to avoid confusion and instead emphasize that our strategy—indirectly blocking eEF1A1 through GBP7 inhibition—may circumvent such challenges. The GBP7–eEF1A1 interaction appears to be functionally engaged specifically during viral infection. Importantly, in contrast to eEF1A1 knockout, Gbp7 knockout cells and mice are fully viable and display no overt phenotypic abnormalities. This key difference suggests that disrupting the GBP7–eEF1A1 interface could impair the viral translation machinery while preserving the essential housekeeping functions of eEF1A1.

We thank the Reviewer for this additional point. We have carefully reviewed and delete the content in **lines 26–28** of the original manuscript.

-Line 60, Not sure if actin binding to eEF1A enables its function as a cofactor in viral replication, the reference 22 does not work with viruses.

**Response:** We thank the Reviewer for the careful evaluation of this statement and for the suggestion to include a biosafety statement. We have addressed both points as detailed below. We acknowledge that the original reference 22 did not directly study viral infection, and we appreciate the Reviewer’s caution. We have now replaced the citation and revised the text to more accurately reflect the supporting evidence. Specifically, we now cite Snape et al. (2018)<https://link.springer.com/article/10.1186/s12985-018-1091-7>, who demonstrated that eEF1A is required for the formation of actin stress fibers and that this function is critical for infectious RSV production (**Revised** **lines 70**).

-Please include a biosafety statement and procedure approval.

**Response:** We sincerely thank the reviewer for this valuable suggestion. We have now added the biosafety statement and procedure approval to the revised manuscript as detailed below (**Revised** **lines 521-528**).

Biosafety Statement: All experiments involving infectious agents were performed in a certified Biosafety Level 2 (BSL-2) laboratory at Southern Medical University‌, in compliance with the guidelines outlined in the CDC/NIH Biosafety in Microbiological and Biomedical Laboratories. The four viruses used in this study, including herpes simplex virus type 1 (HSV-1), Zika virus (ZIKV), dengue virus (DENV), and influenza A virus (IAV) are classified as Risk Group 2 (RG2) agents by the NIH and the World Health Organization (WHO), and all procedures were performed in compliance with biosafety level 2 (BSL‑2) laboratory requirements..

Part II – Major Issues: Key Experiments Required for Acceptance

**Reviewer #1:**

1. The authors used a rabbit reticulocyte lysate in vitro translation system, which contains endogenous eEF1A and GBP family proteins. The current experiments cannot confirm that the enhanced translation is specifically mediated by the GBP7-eEF1A1 axis. It would be convinced that repeating the assay in an eEF1A1-depleted translation system to demonstrate that GBP7's pro-translation effect is strictly dependent on eEF1A1.

**Response:** We sincerely thank the reviewer for this insightful comment. We fully agree that the rabbit reticulocyte lysate‑based TnT Quick Coupled Transcription/Translation System contains endogenous eEF1A1 and GBP family proteins. Unfortunately, selective depletion of endogenous eEF1A1 from this commercial lysate is technically challenging; immunodepletion would likely disrupt the integrity of the translation machinery and severely compromise basal activity, making the interpretation of any residual pro‑translation effect unreliable. Nevertheless, we believe that the function of GBP7 and eEF1A1 is strongly supported by multiple independent lines of evidence presented in our work: 1. When we introduced another gD plasmid in place of the eEF1A1, into the translation system, no significant enhancement of gD protein translation by GBP7 was observed in the absence of exogenously added eEF1A1 (**Revised Fig 5G**). It indicates that GBP7-mediated translational enhancement is markedly restricted in the absence of eEF1A1. 2. As shown in our previous translation assays, eEF1A1 protein was barely detectable in samples without the eEF1A1 plasmid, indicating that the endogenous eEF1A1 level in the translation system is minimal (Fig 5G). A GBP7 mutant without GTPase did not enhance translation in the same system, indicating that the binding interface with eEF1A1 is essential for the pro‑translation activity (Fig 5H). This provides a functional link that is independent of depletion. 3. In cells, siRNA‑mediated knockdown of eEF1A1 significantly blunted the stimulatory effect of GBP7 on global or reporter protein synthesis (S8 Fig). These cellular data mirror the in vitro observations and argue that eEF1A1 is the principal mediator of GBP7’s action. Given these converging results, while we acknowledge that the current Transcription/Translation experiment alone cannot establish strict exclusive dependency, the ensemble of biochemical, mutational and cell‑based data provides compelling evidence that the enhanced translation is specifically mediated by the GBP7 and eEF1A1.

2. The authors claimed that SAH is a "GBP7-targeting inhibitor", but provided no direct biophysical evidence of a direct interaction between SAH and GBP7. Molecular docking and functional assays alone are insufficient to validate target engagement in a top-tier journal. The authors could perform direct binding assays to confirm that SAH directly binds to GBP7, and validate that the binding occurs at the predicted R48/K51 active site via mutagenesis.

**Response:** We appreciate the reviewer’s strict requirement for direct binding evidence. We have now performed complementary biophysical and biochemical assays:

1. Surface plasmon resonance (SPR): Concentration‑gradient SPR analysis revealed dose‑dependent binding of SAH to immobilized recombinant GBP7. This directly demonstrates a physical SAH–GBP7 interaction（**Revised Fig 6C**）.

2. Drug affinity responsive target stability (DARTS): SAH protected recombinant GBP7 against pronase digestion in a dose‑dependent manner, confirming high‑affinity target engagement in a near‑native state (**Revised Fig 6B**).

3. Binding‑site validation by mutagenesis: In DARTS assays, the GBP7^R48A^ and GBP7^K51A^ double mutant exhibited markedly reduced protection by SAH compared with wild‑type GBP7, validating that the predicted R48/K51 pocket is the critical binding site (**Revised S11 Fig**).

Collectively, these new results provide direct biophysical evidence that SAH specifically binds GBP7 at the R48/K51 active site, firmly supporting its designation as a GBP7‑targeting inhibitor.

**Reviewer #2:**

1. The study only shows that GBP7 promotes viral protein translation (such as gD) but does not exclude global effects on host translation in Fig.5. The author needs to provide evidence that GBP7 preferentially enhances viral translation with minimal impact on host translation, supporting target safety and mechanistic specificity. The author can perform Puromycin Incorporation Assay to measure overall protein synthesis rate.

**Response:** We thank the reviewer for this important mechanistic question. We agree that distinguishing between effects on viral translation versus global host translation is critical for establishing both the specificity of GBP7 function and the safety of targeting this factor. Our previous observations indicate that GBP7 is expressed at very low basal levels in uninfected cells, and its knockdown or overexpression does not appreciably affect normal cellular physiology. Notably, GBP7 expression is robustly induced upon viral infection, suggesting that its function is tightly linked to the infectious state rather than to routine host translation. In our cellular and in vitro assays, we observed that GBP7 not only promotes viral protein synthesis but also moderately influences the expression of certain host proteins, consistent with its role as a general positive modulator of translation (**Revised Fig 5**). However, this modulation does not compromise overall cell viability or proliferation, as demonstrated by our cytotoxicity and cell growth assays in both GBP7‑KO and SAH‑treated cells (**Revised S3 and S12 Figs**).

While the puromycin incorporation assay could provide a direct readout of nascent protein synthesis, it does not inherently distinguish between viral and host translation, as both depend on the same host translational machinery. Thus, the functional specificity of GBP7 is best reflected by its infection‑dependent expression pattern and the observation that its modulation exerts minimal impact on host cell fitness while significantly suppressing viral replication. Furthermore, the safety of GBP7 targeting is supported by both in vitro cytotoxicity data and in vivo tolerability assessments, reinforcing its potential as a selective antiviral target.

2. Figure 6 serves as the core panel for drug screening and target validation in this study. It was only shown a in silico docking prediction in Fig. 6A. No experimental data (MST/SPR/ITC) are provided to confirm direct physical binding of SAH to GBP7, which is a critical gap in target validation. Direct binding assays must be added and included in the figure. Besides, functional validation using R48A or K51A mutants need to show that SAH loses inhibitory activity against GTPase dead variants, confirming on target activity.

**Response:** We appreciate the reviewer’s strict requirement for direct binding evidence. To address this, we have now performed complementary biophysical and biochemical assays to rigorously validate the SAH–GBP7 interaction. First, surface plasmon resonance (SPR) analysis with concentration gradients revealed dose‑dependent binding of SAH to immobilized recombinant GBP7, directly demonstrating a physical interaction between the compound and the protein (**Revised Fig 6C**). Additionally, drug affinity responsive target stability (DARTS) assays showed that SAH protected recombinant GBP7 against pronase digestion in a dose‑dependent manner, confirming high‑affinity target engagement under near‑native conditions (**Revised Fig 6B**). Finally, to validate the predicted binding site, we generated GBP7^R48A^ and GBP7^K51A^ double mutants and found that they exhibited markedly reduced protection by SAH in DARTS assays compared with wild‑type GBP7, confirming that the R48/K51 pocket serves as the critical binding interface (**Revised S11 Fig**). Collectively, these new results provide direct biophysical evidence that SAH specifically binds GBP7 at the R48 or K51 active site, firmly supporting its designation as a GBP7‑targeting inhibitor.

3. The study only shows that HSV‑1 induces GBP7 expression but does not explore whether IFN‑α/β/γ, NF‑κB, or JAK‑STAT pathways mediate this induction, leaving the regulatory mechanism unclear.

**Response:** We thank the reviewer for raising the upstream regulatory aspect of GBP7 induction. While our initial focus was on HSV-1, our extended analysis of GBP7's antiviral spectrum revealed that infection with VSV, IAV, DENV, and ZIKV also robustly upregulates GBP7 expression, and that GBP7 knockout consistently attenuates replication across these diverse viruses (Fig 8). This broad activation pattern suggests a common, virus‑triggered host response rather than a pathogen‑specific event. In agreement with this, it is well established in the literature that several members of the guanylate‑binding protein (GBP) family are interferon‑stimulated genes, predominantly induced via the IFN‑γ/JAK‑STAT axis [(https://rupress.org/jem/article/216/3/482/120360/Interferon-induced-guanylate-binding-proteins)](https://rupress.org/jem/article/216/3/482/120360/Interferon-induced-guanylate-binding-proteins) <(https://www.sciencedirect.com/science/article/pii/S0952791523000924?via%3Dihub).> Given that this upstream signaling cascade has been extensively characterized, our study deliberately focused on the downstream effector function of GBP7—specifically its role in translational regulation—rather than reiterating the well‑documented mechanisms governing its transcriptional activation. We hope that our responses have satisfactorily addressed your queries. Given that the present study is primarily focused on establishing GBP7 as an antiviral target and elucidating its mechanism of action, the involvement of GBP7 in immune signal transduction pathways warrants further investigation and is therefore not extensively discussed in this manuscript.

**Reviewer #3:**

Experiments to show the cytotoxic/cytostatic effects related to blocking GBP7 should be provided, along clear IC_50_ and CC_50_ curves of SAH. Moreover, antiviral activity should be shown using viral protein measurements or CPE rather than PCRs.

**Response:** We thank the reviewer for the suggestions. We have now provided the IC50 of SAH. (**Revised Fig 6G**), and blockade of GBP7 did not significantly affect cell proliferation efficiency (**Revised S3 Fig**). We previously assessed the cytotoxicity of SAH in A549 cells, and have now extended these measurements to include Hela and Vero cells (**Revised S12 Fig**). Owing to solubility limitations, the maximum concentration of SAH examined was 200 μM. In the revised manuscript, we have performed additional Western blotting analyses to confirm that SAH treatment inhibits viral protein expression across multiple viruses, including VSV, IAV, DENV, and ZIKV, which supports the initial exploration of SAH and GBP7 as potential broad‑spectrum antiviral candidates (**Revised Fig 6D**).

**Part III – Minor Issues: Editorial and Data Presentation Modifications**

Please use this section for editorial suggestions as well as relatively minor modifications of existing data that would enhance clarity.

**Reviewer #1:**

1. The observation that GBP7 knockout or SAH treatment also inhibits VSV, IAV, DENV, and ZIKV is intriguing, but the authors do not demonstrate that the GBP7-eEF1A1 axis mediates these effects. Therefore, the claim of a "broad-spectrum" mechanism should be tempered. Please revise the text to present this as an initial or preliminary observation that requires future validation.

**Response:** We thank the reviewer for this constructive suggestion and fully agree with the assessment. We have revised the text throughout the manuscript to temper our claims regarding the broad-spectrum mechanism. We have also explicitly noted that future studies are needed to determine whether the GBP7-eEF1A1 axis mediates the antiviral effects against these viruses.

2. The manuscript states survival analysis was performed via the Kaplan–Meier method, but does not report the statistical test used for between-group survival comparisons (the gold standard is the log-rank test). This detail can be added to the main text and figure legend, along with details of normality and variance homogeneity testing for all statistical analyses.

**Response:** We thank the reviewer for this important methodological suggestion. We have now added the statistical details throughout the manuscript. In the Methods section, we now state that between-group survival comparisons were analyzed using the log-rank (Mantel-Cox) test. We have also added descriptions of normality testing (Shapiro-Wilk test) and variance homogeneity testing (Levene's test) for all statistical analyses, and clarified that for normally distributed data with equal variances, two-tailed unpaired Student's t-test or one-way ANOVA followed by Tukey's post-hoc test was used; for non-normally distributed data or unequal variances, the Mann-Whitney U test or Kruskal-Wallis test was applied. These details have also been incorporated into the relevant figure legends.

3. Figure 2 shows that the inhibition of virus replication is not significant at the early time point, but is more obvious at the later time point, which may be the result of enhanced antiviral signal transduction.

**Response:** We thank the reviewer for this insightful comment. We fully agree that the more pronounced antiviral effect observed at later time points may reflect enhanced host antiviral responses. GBP7 is a pro‑viral host factor that promotes HSV‑1 replication by enhancing viral protein translation. Therefore, in the absence of GBP7, viral protein synthesis is reduced, and the antiviral signaling pathways that are normally suppressed by GBP7 become de‑repressed, leading to a stronger antiviral state at later stages of infection. Given that GBP7 functions primarily during the late phase of the viral replication cycle, the antiviral effect of GBP7 knockout is expected to become more evident after 24 hours, when late gene expression and viral production are most active.

4. Lines 37-38: The authors incorrectly group cidofovir (a nucleotide analog active against TK-deficient HSV) and pritelivir (a helicase-primase inhibitor) with TK-dependent nucleoside analogues, and include an unapproved drug "anisuvir" with no clinical indication for HSV. These errors should be corrected to ensure accurate background context.

**Response:** We thank the reviewer for catching these inaccuracies. We have corrected the text to accurately describe cidofovir as a nucleotide analog active against TK-deficient HSV strains, and pritelivir as a helicase-primase inhibitor. We have also removed the reference to anisuvir, as it is not an FDA-approved drug for HSV infection (**Revised** **Lines 34-38**).

5. Line 67-68: The manuscript includes an internal revision comment ("This revision more clearly communicates the discovery and the functional role of eEF1A1") in the main body of the Introduction. This non-scientific text can be deleted immediately.

**Response:** We apologize for this oversight. The internal comment has been removed from the revised manuscript.

6. Line 160: The manuscript uses the incorrect term "GTPase effect domain" (standard nomenclature is "GTPase effector domain, GED"), and repeatedly misclassifies eEF1A1’s function as "initiating translation" (eEF1A1 mediates translation elongation, not initiation).

**Response:** We thank the reviewer for these important terminological corrections. We have revised the manuscript throughout to use the standard term "GTPase effector domain (GED)" and have corrected all references to eEF1A1 function from "translation initiation" to "translation elongation."

7. Line 356: The reported puromycin selection concentration of "5 mg/ml" is ~1,000-fold higher than the standard working concentration (1–10 μg/ml) and will cause universal cell death; this is almost certainly a critical typo that must be verified and corrected.

**Response:** We thank the reviewer for catching this critical error. We have verified the correct concentration and corrected the typo from "5 mg/ml" to "5 μg/ml" in the revised manuscript (**Revised** **Line 487**).

**Reviewer #2:** 1. In Figure 1, GBP7 induction by HSV‑1 is only validated in A549 cells, without confirmation in HSV‑1‑permissive cells such as Vero, Hela, or primary keratinocytes. The author only showed qualitative IF images without quantification of fluorescence intensity, so the difference in GBP7 expression cannot be objectively evaluated in Fig. 1C and Fig.1G.

**Response:** We thank the reviewer for this valuable suggestion. To address this, we have extended our analysis to include Vero cells and Vero cells. GBP7 induction upon HSV-1 infection was consistently observed across all three cell types, and these results are now presented in **Revised S1 Fig**. In addition, we have performed quantitative analysis of the immunofluorescence images shown in Fig. 1C and Fig. 1G. Using ImageJ, we measured the mean fluorescence intensity per cell from three independent experiments. (**Revised Figs 1C and G, Fig2 J, S2 and S4 Figs**). These quantifications confirm that the observed differences in GBP7 expression are statistically significant and objectively evaluated.

2. In Figure 2, the author used single viral MOI lacking dose‑dependence validation (MOI=0.1). No low/medium/high viral gradient is included, so the dose‑dependent proviral effect of GBP7 cannot be confirmed, reducing the robustness of the conclusion. The quality of the fluorescence images in Figure 2K is very poor, the authors should provide clearer images. Also, Panel 2K only shows qualitative images without quantification of fluorescence intensity or GFP‑positive cell percentage, providing only descriptive rather than statistical evidence.

**Response:** We thank the reviewer for these important suggestions. To address the MOI dose‑dependence, we have performed additional experiments infecting with a gradient of viral MOIs (0.1, 1, 2). The proviral effect of GBP7 was consistently observed across all MOIs tested (**Revised S1B Fig)**. Due to limitations in confocal microscopy and the relatively weak GFP fluorescence of the virus, we were unable to obtain clearer images. But we have quantified both the percentage of GFP‑positive cells and the mean fluorescence intensity from three independent experiments, which are now presented as bar graphs in revised (**Revised Fig 2J)**. These quantitative data demonstrate a significant reduction in HSV-1/GFP in GBP7‑KO cells, providing statistical rather than merely descriptive evidence.

3. Current data identified the binding and interaction between GBP7 and eEF1A1 rely on IP-MS, Co-IP, co-localization, and docking in Fig. 4. In vitro pull‑down assay using purified proteins should be used to confirm direct binding.

**Response:** We appreciate the reviewer’s suggestion. While we agree that in vitro pull‑down assays would provide complementary evidence for a direct GBP7–eEF1A1 interaction, our current mechanistic model supports a functional association rather than a stable binary complex, given that GBP7 is thought to facilitate eEF1A1‑mediated translation elongation through its GTPase activity. Accordingly, the interaction is likely transient or indirectly mediated, and the precise molecular details remain to be defined. This point has been explicitly discussed in the revised Discussion as a priority for future investigation. We hope that the Co‑IP, co‑localization, and functional data collectively provide a solid basis for the GBP7 and eEF1A1 complex.

4. In Figure 5, the R48A and K51A mutants of GBP7 were only tested for GTPase activity and proviral function. Their binding ability to eEF1A1 was not examined, making it impossible to rule out indirect effects from altered interaction.

**Response:** We thank the reviewer for this critical point. To address this, we have performed Co‑IP experiments comparing the binding affinity of wild‑type, R48A, and K51A GBP7 mutants to eEF1A1. Both R48A and K51A mutants retained comparable binding to eEF1A1 relative to wild‑type GBP7 (Fig. 5D), indicating that the loss of GTPase activity and proviral function is due to impaired GTP hydrolysis rather than disrupted eEF1A1 binding. This is consistent with the structural prediction that R48 and K51 are located within the GTPase active site rather than the eEF1A1 interaction interface.

5. The author used only three concentrations without reporting EC50, CC50, or selectivity index (SI) in Fig. 6B–D, making it impossible to evaluate the antiviral potency and safety of SAH objectively. Full dose–response curves and calculated parameters are required.

**Response:** We thank the reviewer for this important methodological suggestion. We have now performed full dose–response curves using ten‑point serial dilutions of SAH in HSV‑1‑infected A549 cells. The EC_50_ was calculated as 3.491 µM, and the CC_50_ was determined as >200 µM, yielding a selectivity index (SI) of >57.29 (**Revised Fig 6G, S12 Fig**). These data provide a comprehensive evaluation of SAH's antiviral potency and safety profile.

6. In Figure7, the author demonstrated SAH effectively alleviated symptoms and improved survival rate of mice in HSV-1 infection. However, the in vivo pharmacokinetic (PK) data for SAH was loss, these plasma concentration, half‑life, and tissue distribution were missing, hindering interpretation of dose–response relationship. Meanwhile, H&E images in Fig.7H were only qualitative without lesion or inflammation scoring.

**Response:** We thank the reviewer for this valuable comment. We fully acknowledge the lack of PK data and quantitative histopathological scoring in the original figure, which limits the interpretation of the dose–response relationship. In this study, our primary objective is to establish GBP7 as a bona fide antiviral target, and the in vivo efficacy data are intended as proof‑of‑concept evidence to support this notion, rather than to fully characterize SAH as a lead candidate. Given that the current compound has not yet been optimized to an ideal activity level, we consider that comprehensive PK studies at this stage would be premature and could consume substantial resources with limited informative value. Regarding the H&E images, we agree with the reviewer that quantitative scoring would strengthen the analysis; however, we would like to note that the differences in inflammatory infiltration between groups were conspicuous. We hope that these revisions adequately address the reviewer’s concerns.

7. In Figure 8, the validation of broad‑spectrum antiviral activity is inadequate. The authors only detected GBP7 and viral gene expression at the mRNA level upon infection with diverse viruses but lacked confirmation of GBP7 protein induction and direct measurement of progeny virus titers. No dose–response curves or EC50 values were determined for SAH’s broad‑spectrum activity, making it impossible to objectively evaluate its antiviral potency. Ribavirin, used as a positive control, was only applied at a single dose without parallel comparative analysis. Furthermore, all experiments were performed in cancer cell lines without validation in primary cells or in vivo models. In addition, the common mechanism by which GBP7 regulates both DNA and RNA viruses was not thoroughly explored.

**Response:** We sincerely thank the reviewer for the valuable and detailed suggestions regarding the broad‑spectrum antiviral activity of SAH. In the revised manuscript, we have performed additional Western blotting analyses to confirm that SAH treatment inhibits viral protein expression across multiple viruses, including VSV, IAV, DENV, and ZIKV, which supports the initial exploration of SAH and GBP7 as potential broad‑spectrum antiviral candidates (**Revised Fig 6D**). The inclusion of ribavirin as a positive control was intended solely to validate the feasibility of the infection models, rather than to serve as a direct comparator for efficacy assessment. We fully acknowledge that the current dataset represents an initial assessment of broad‑spectrum activity, and we recognize the limitations in evaluating progeny virus titers, establishing dose‑response curves, and defining EC₅₀ values for each virus. In this study, we aimed to provide preliminary evidence for the broad‑spectrum potential of SAH, which we believe is supported by the consistent inhibition observed at the mRNA and protein levels. We also agree that validation in primary cells and in vivo models would significantly strengthen these findings. However, we would like to point out that establishing robust in vivo infection models for multiple viruses is technically challenging and resource‑intensive, and we consider this as an important direction for our future studies. We have revised the Discussion to clearly state these limitations and to outline our ongoing efforts to further dissect the common mechanisms by which GBP7 regulates both DNA and RNA viruses, as well as to develop more comprehensive pharmacological evaluations in appropriate models.

8. Provide quantification graphs for all Western blots, some panels currently show only bands without statistics.

**Response:** We thank the reviewer for this constructive suggestion. In the revised manuscript, we have performed densitometric quantification for selected Western blots, particularly where the GAPDH loading controls were less uniformly matched or where the differences were not directly discernible by visual inspection. However, we believe that including quantification graphs for all blots would make the figures excessively crowded and impede readability, especially for panels where the differences are visually unambiguous. We have therefore prioritized quantification for those panels where statistical support is most critical and have included the corresponding data in the revised figures or supplementary materials where appropriate. We trust that this approach appropriately balances scientific rigor with clarity of presentation. We will provide additional quantifications if the reviewer deems them necessary.

9. The author needs to conduct an in-depth discussion on why interferon-inducing genes have the evolutionary significance for viruses, addressing whether it represents a viral hijacking mechanism or a form of host immune homeostasis regulation. In addition, the authors should further compare the antiviral mechanisms of other GBP family members (GBP1/2/5), particularly in the herpesvirus infection, and explain the structural basis underlying the distinct functional properties of GBP7.

**Response:** Thank you for this insightful and constructive comment. The points raised are highly valuable and have helped improve the conceptual depth and mechanistic clarity of the revised manuscript. (1) Interferon-inducing genes should not be interpreted solely through a binary framework of either viral hijacking or host-beneficial immunity. Rather, their biological significance likely reflects a dynamic evolutionary balance between antiviral defense and immune homeostasis. On the one hand, viruses have evolved diverse strategies to exploit, evade, or reprogram interferon-related pathways for their own replication and persistence. This supports the concept that some interferon-associated host factors may become functionally co-opted during infection, thereby contributing to a “viral hijacking” phenomenon in specific contexts. On the other hand, the induction of these genes is fundamentally part of the host innate immune architecture, which has evolved to detect infection rapidly, restrict pathogen spread, and limit excessive immunopathology. Therefore, the expression and function of interferon-inducing genes may also represent an adaptive host strategy to maintain immune equilibrium while preserving antiviral activity. (2) GBP1 and GBP2 are now discussed as classical interferon-inducible large GTPases that can localize to pathogen-associated membranous compartments and participate in intracellular antimicrobial defense. Their antiviral activities are generally linked to GTPase-dependent membrane remodeling, interference with intracellular trafficking, and promotion of innate immune signaling. In the setting of herpesvirus infection, these proteins have been implicated in restricting stages of viral replication through modulation of cellular membrane platforms, trafficking pathways, and inflammatory signaling networks, although the precise mechanisms may differ according to the virus and cell type studied. GBP5 is now discussed as a functionally distinct GBP family member whose antiviral properties have frequently been associated with regulation of host membrane-associated processes and innate immune activation. In several viral systems, GBP5 has been shown to interfere with viral protein processing, envelope maturation, or infectivity, and it may also shape antiviral immunity by modulating signaling cascades. In contrast, GBP7 appears to display a distinct functional profile within the GBP family. The revised Discussion now emphasizes that, unlike GBP1/2/5, whose antiviral effects are more often interpreted in relation to membrane targeting, inflammasome-associated signaling, or interference with viral maturation, GBP7 may exert its role through a different balance of subcellular localization, oligomeric behavior, or immune regulatory interactions. This distinction is now more clearly articulated to position GBP7 within the broader functional landscape of GBP-mediated antiviral immunity. (3) As with other guanylate-binding proteins, GBP7 contains a conserved N-terminal large GTPase domain that is expected to support nucleotide binding and hydrolysis, as well as a more variable C-terminal helical domain that may influence protein-protein interactions, conformational dynamics, subcellular targeting, and oligomerization behavior. However, compared with GBP1, GBP2, and GBP5, GBP7 appears to possess differences in sequence composition and/or domain organization that may alter these biochemical properties. We now propose that these structural characteristics may provide a mechanistic explanation for why GBP7 exhibits functional properties that are not fully overlapping with those of other GBP family members. In particular, if GBP7 differs in membrane-binding determinants, post-translational modification sites, or interfaces that mediate intermolecular interactions, this could account for distinct antiviral specificity, signaling outputs, or cellular localization patterns. Because high-resolution structural and biochemical studies of GBP7 remain limited, we have taken care to present this section in a cautious manner and to distinguish clearly between established knowledge from other GBP proteins and reasonable mechanistic inferences for GBP7.

We sincerely appreciate the reviewer's insightful comments. The above discussion was to fully address the reviewer's concerns regarding the evolutionary significance of interferon-inducing genes, the comparative antiviral mechanisms of GBP family members, and the structural basis of GBP7. However, we respectfully propose that this content not be incorporated into the revised manuscript, as the primary focus of the present study is to investigate the feasibility and mechanism of action of GBP7 as a host target for antiviral therapy, rather than a systematic review of GBP7-mediated immune signaling or the evolutionary dynamics of the GBP family. These topics have been extensively addressed in prior publications (Legewie et al., Biochem J, 2019; Braun et al., Cell Rep, 2019; Cui et al., PNAS, 2021). Incorporating such content would risk shifting the thematic emphasis away from our core findings and expanding the Discussion beyond the scope of the original study. Should the reviewer consider it necessary, we are willing to add a brief acknowledgment of these broader aspects with appropriate citations in the revised manuscript.

**Reviewer #3**: (No Response)
