# Supplementary material for: GTPase GBP7 promotes HSV-1 infection by interacting with and activating eEF1A1 to accelerate the protein translation process
Source: PLoS Pathog. 2026 Oct 12;22(10):e1014652. doi: 10.1371/journal.ppat.1014652 (PMC13759050; doi:10.1371/journal.ppat.1014652)
Supplement: Peer review history

# Peer review: Decision Letter 0

**DOI:** 10.1371/journal.ppat.1014652.r001

**Authors:** Alison McBride, Kai Zheng

21 May 2026

PPATHOGENS-D-26-00828

GTPase GBP7 promotes HSV-1 infection by interacting with and activating eEF1A1 to

accelerate the protein translation process

PLOS Pathogens

Dear Dr. Liu,

Thank you for submitting your manuscript to PLOS Pathogens. After careful consideration, we feel that it has merit but does not fully meet PLOS Pathogens's publication criteria as it currently stands. Therefore, we invite you to submit a revised version of the manuscript that addresses the points raised during the review process.

Please submit your revised manuscript by Jul 20 2026 11:59PM. If you will need more time than this to complete your revisions, please reply to this message or contact the journal office at plospathogens@plos.org. When you're ready to submit your revision, log on to https://www.editorialmanager.com/ppathogens/ and select the 'Submissions Needing Revision' folder to locate your manuscript file.

Please include the following items when submitting your revised manuscript:

* A letter that responds to each point raised by the editor and reviewer(s). You should upload this letter as a separate file labeled 'Response to Reviewers'. This file does not need to include responses to any formatting updates and technical items listed in the 'Journal Requirements' section below.

* A marked-up copy of your manuscript that highlights changes made to the original version. You should upload this as a separate file labeled 'Revised Manuscript with Track Changes'.

* An unmarked version of your revised paper without tracked changes. You should upload this as a separate file labeled 'Manuscript'.

If you would like to make changes to your financial disclosure, competing interests statement, or data availability statement, please make these updates within the submission form at the time of resubmission. Guidelines for resubmitting your figure files are available below the reviewer comments at the end of this letter. As the corresponding author, your ORCID iD is verified in the submission system and will appear in the published article. PLOS supports the use of ORCID, and we encourage all coauthors to register for an ORCID iD and use it as well. Please encourage your coauthors to verify their ORCID iD within the submission system before final acceptance, as unverified ORCID iDs will not appear in the published article. Only the individual author can complete the verification step; PLOS staff cannot verify ORCID iDs on behalf of authors.

We look forward to receiving your revised manuscript.

Kind regards,

Kai Zheng

Academic Editor

PLOS Pathogens

Alison McBride

Section Editor

PLOS PathogensSumita Bhaduri-McIntosh

Editor-in-Chief

PLOS Pathogens

orcid.org/0000-0003-2946-9497

Michael Malim

Editor-in-Chief

PLOS Pathogens

orcid.org/0000-0002-7699-2064

**Journal Requirements:**

If the reviewer comments include a recommendation to cite specific previously published works, please review and evaluate these publications to determine whether they are relevant and should be cited. There is no requirement to cite these works unless the editor has indicated otherwise.

1) Please ensure that the CRediT author contributions listed for every co-author are completed accurately and in full.

At this stage, the following Authors/Authors require contributions: Jinshen Wang, Binhao Rong, Liangbing Man, Kunyu Lu, Luping Cheng, Jianfu He, Haowei Li, Hao Su, Xinxin Lin, Ru Li, Min Zou, Chan Yang, and Shuwen Liu. Please ensure that the full contributions of each author are acknowledged in the "Add/Edit/Remove Authors" section of our submission form.

The list of CRediT author contributions may be found here: https://journals.plos.org/plospathogens/s/authorship#loc-author-contributions

2) We ask that a manuscript source file is provided at Revision. Please upload your manuscript file as a .doc, .docx, .rtf or .tex. If you are providing a .tex file, please upload it under the item type u2018LaTeX Source Fileu2019 and leave your .pdf version as the item type u2018Manuscriptu2019.

3) We do not publish any copyright or trademark symbols that usually accompany proprietary names, eg ©,  ®, or TM  (e.g. next to drug or reagent names). Therefore please remove all instances of trademark/copyright symbols throughout the text, including:

- ® on pages: 20, 23, 24, 44, 47 line no 404, 465, 467, 468, and 484.

4) Please upload all main figures as separate Figure files in .tif or .eps format. For more information about how to convert and format your figure files please see our guidelines:

https://journals.plos.org/plospathogens/s/figures

5) Please upload a copy of Figure Figures 1-8 which you refer to in your text on pages 6, 7, 8, 10, 12, and 13. Or, if the figure is no longer to be included as part of the submission please remove all reference to it within the text.

6) We have noticed that you have uploaded Supporting Information files [Supplementary figures 8 and supplementary tables 6], but you have not included a list of legends. Please add a full list of legends for your Supporting Information files after the references list.

8) Please amend your detailed Financial Disclosure statement. This is published with the article. It must therefore be completed in full sentences and contain the exact wording you wish to be published.

1) State the initials, alongside each funding source, of each author to receive each grant. For example: "This work was supported by the National Institutes of Health (####### to AM; ###### to CJ) and the National Science Foundation (###### to AM)."

2) State what role the funders took in the study. If the funders had no role in your study, please state: "The funders had no role in study design, data collection and analysis, decision to publish, or preparation of the manuscript.".

If you did not receive any funding for this study, please simply state: u201cThe authors received no specific funding for this work.u201d

**Reviewers' Comments:**

Reviewer's Responses to Questions

**Part I - Summary**

Please use this section to discuss strengths/weaknesses of study, novelty/significance, general execution and scholarship.

Reviewer #1: This manuscript by Wang et al. delineates an unexpected proviral function of guanylate-binding protein 7 (GBP7). GBP7 promotes HSV-1 infection by interacting with and activating eEF1A1 to accelerate the protein translation process. This work addresses a critical gap in the functional characterization of the GBP family, and uncovers a novel mechanism by which HSV-1 hijacks the host innate immune response to support its replication. The identification of GBP7 as a host-directed antiviral target has important translational value for overcoming drug resistance in HSV-1 infection. It is of broad interest to the fields of virology, innate immunity and antiviral drug development, pending resolution of the following concerns.

Reviewer #2: This study systematically investigates the function and molecular mechanism of GBP7 during herpes simplex virus type 1 (HSV-1) infection using a comprehensive, multi‑level experimental design. The work reports for the first time that GBP7, a canonical interferon‑inducible GTPase, does not function as an antiviral factor but instead significantly promotes HSV-1 replication and infection. The authors further elucidate a novel regulatory axis: GBP7 directly binds eEF1A1, enhances GTP hydrolysis, and accelerates viral protein translation elongation. Additionally, virtual screening identifies SAH as a GBP7‑targeted inhibitor with potent anti‑HSV-1 and broad‑spectrum antiviral activity in cells and mice. Overall, this study features high novelty, rigorous logic, sufficient experimental evidence, and standardized methodology. It not only expands understanding of the GBP protein family but also provides a novel target and candidate molecule for host‑directed broad‑spectrum antiviral drug development, with important scientific significance and translational potential. However, there are deficiencies in aspects such as the rigor of experimental design, the depth of mechanism verification, the accuracy of result interpretation, and the expansion of significance, which need to be further improved to enhance the scientificity and influence of the research.

Reviewer #3: In this manuscript, Wang and collogues perform a detailed characterization of the role of the GBP7 GTPase in the activation of eEF1A1, showing how this interaction favors HSV-1 viral production. However, critical controls and clearer explanations are needed to improve the robustness of the data provided, as suggested in the key points of this revision. Findings are relevant as they reveal for the first time how GBP7 impacts the activity of eEF1A on protein translation, and how the modulation of GBP7 can lead to a different approach to develop broad spectrum therapies. This indeed aligns with prior evidence on eEF1A blockade, for which a broad-spectrum antiviral activity has been recently identified, what adds to the prior well-known activity of drugs targeting eEF1A that have shown antiviral activity in vitro and in vivo, being safe in several clinical trials. This information is currently missing from the manuscript and could help to strengthen the findings of this paper, contextualizing several assumptions of the authors in the discussion section, where there are several claims that are not sustained by the current available evidence.

Main comments to the authors:

1. In the introduction (lines 59-61), the authors state that eEF1A is a host factor required for the replication of different viruses, but they cite reviews, when there are primary references showing how antivirals targeting eEF1A inhibit the replication of multiple viruses, including HSV-1, which are the following:

*Molina Molina E, et al. Targeting eEF1A reprograms translation and uncovers broad-spectrum antivirals against cap or m6A protein synthesis routes. Nat Commun. 2025 Feb 7;16(1):1087. doi: 10.1038/s41467-025-56151-y. PMID: 39920115; PMCID: PMC11805953.

*Albericio G, Rodríguez-Martín D, Avilés P, Cuevas C, Guillén-Navarro MJ, Noriega MA, Flores S, Sánchez-Cordón PJ, Astorgano D, Pérez P, Esteban M, García-Arriaza J. Functional characteristics of plitidepsin as an antiviral treatment against monkeypox virus infection. Antiviral Res. 2025 Sep;241:106238. doi: 10.1016/j.antiviral.2025.106238. Epub 2025 Jul 15. PMID: 40675496.

In line 64 and 65, it is also stated that inhibition of eEF1A is a promising avenue for antiviral efficacy, but no references are given, when clinical trials have already shown safety and efficacy of molecules targeting this host factor:

*Varona, J.F., et al., 2022. Preclinical and randomized phase I studies of plitidepsin in adults hospitalized with COVID-19. Life Sci. Alliance 5, e202101200. https://doi.org/10.26508/lsa.202101200

*Landete, P., et al, J.-F., 2024. A phase III randomized controlled trial of plitidepsin, a marine derived compound, in hospitalized adults with moderate COVID-19. Clin Infect Dis ciae227. https://doi.org/10.1093/cid/ciae227

Finally, in lines 78-81 the authors mention the role of GBP7-eEFA1 axis as a broad spectrum antiviral approach, but this was already known for plitidepsin, a drug blocking eEFA1 that inhibits viral replication in vitro and in vivo of several viruses (please, see the above mentioned references) and has shown safety in clinical trials (see the above mentioned references).

2. The authors plot and compare viral and cellular mRNA transcripts in a linear scale in most of the figures of this manuscript, and they say that they refer it to GDPAH. This analysis can be biased, as blocking protein translation leads to a cytostatic effect that decreases cell division, and therefore, if no controls are provided to show that the same number of cells are added, interpretations can be misleading. Moreover, I am having a hard time to understand why an inhibitor of protein translation is having an impact on viral mRNA 24h pi, when most likely no new infections are taking place and similar viral mRNAs would be found in the cell. This is precisely what the authors see in the animal model in Fig 3G, where no change in RNA is detected. Indeed, if GBP7 is overexpressed between 24 to 48 hpi (Fig 1) and upon knockdown it can decrease viral mRNA 24 h pi (Fig 2), how do the authors explain that in the murine KO model, there is no decrease in viral mRNA 3 dpi (Fig. 3G) and no differences in viral infectivity measured as viral titers (Fig. 3G)?

While protein quantification experiments are clear to me and support the pathway involving GBP7 and eEF1A in boosting protein translation, experiments showing RNA levels are confusing, so the authors should focus on the effects observed at protein level, especially at short times post infection.

2. Do GBP7-KO cell lines have lower viability or cell division rates as compared to controls transduced with empty vectors? And is it similar to the viability and cell division rates observed for eEF1A KO cells, in Fig S6? eEF1A inhibitors are known to display a cytostatic effect that has been very useful in the cancer field, so this should be controlled for too, as this could explain decreases in mRNA.

4. Authors state that the complex formed by GBP7 and eEF1A is increased upon infection (4E), but this is not quantified and no colocalization data is provided.

5. In the antiviral screening, there is no calculation of the toxicity (CC50) of SAH, via MTT, cell titer glo or other method. No IC50 is provided either. In addition, whether blocking GBP7 has a cytostatic effect is not analyzed. Again, from a mechanistic point of view, it is not clear to me why the mRNA of HSV VP16 is decreased 24h post infection (Fig 6D). If we assume that GBP7 inhibition is blocking viral translation via inactivation of eEF1A, it should affect viral protein synthesis, not viral mRNA, as previously observed in the KO animal model, where differences were detected 5 dpi. Assessing protein quantification will provide more robust evidence. In addition, a comparison with known inhibitors that block eEF1A, such as plitidepsin, would be helpful to confirm these findings.

6. In lines 239-241, authors state that SAH not only downregulates the expression of GBP7 protein in cells but also impairs GTPs activity. However, if infection increases GBP7 and SAH blocks infection, it is expected that GBP7 would be expressed to a lower extent, but not due to a direct action of SAH. Most likely, dugs like ACV would lead to the same effect. This should be clarified.

7. If animals in figure 7 were followed over time, why do authors only show titration and VP16 mRNA levels at one timepoint? What is the kinetics of SAH viral inhibition, measured by protein quantification? Given that the best response is obtained using 200 mg/Kg, which is a very high drug dose, what are the chances to move the use of this antiviral further for clinical use? This should be discussed as a limitation.

8. Broad spectrum activity of SAH is only based on mRNA quantification 24h post infection without any control for cytotoxicity and cytostatic effects of the drug. Additional evidence, such as measuring the impact on viral protein production, which is the expected MoA of SAH when blocking GBP7, will be needed to demonstrate the MoA. Moreover, a side-by-side comparison with plitidepsin, that blocks eEF1A will reinforce this MoA. Complementary assays to show the impact on viral replication, including CPE measurement, will be needed to show the broad-spectrum activity of SHA beyond RNA levels. In addition, calculation of IC50 and CC50 for SAH in each viral model should be provided.

9. In the discussion section, authors state “However, the translation elongation process is indispensable for intracellular protein production. Targeting eEF1A1 may affect the normal vital activities of cells and even lead to cell death and, potentially, the death of individuals”. This statement completely ignores current evidence, as several studies have confirmed that targeting eEF1A with plitidepsin does not affect cell viability at the nM concentrations where antiviral effect is achieved. This is because protein translation is not completely shut down, as alternative m6A protein translation routes are not affected by blocking eEF1A and cells can synthesize cellular proteins in the presence of plitidepsin, as shown in:

*Molina Molina E, et al. Targeting eEF1A reprograms translation and uncovers broad-spectrum antivirals against cap or m6A protein synthesis routes. Nat Commun. 2025 Feb 7;16(1):1087. doi: 10.1038/s41467-025-56151-y. PMID: 39920115; PMCID: PMC11805953.

There are also several clinical trials showing the safety of plitidepsin in hospitalized COVID-19 individuals, where no death was reported, as published in:

*Varona, J.F., et al., 2022. Preclinical and randomized phase I studies of plitidepsin in adults hospitalized with COVID-19. Life Sci. Alliance 5, e202101200. https://doi.org/10.26508/lsa.202101200

*Landete, P., et al, J.-F., 2024. A phase III randomized controlled trial of plitidepsin, a marine derived compound, in hospitalized adults with moderate COVID-19. Clin Infect Dis ciae227. https://doi.org/10.1093/cid/ciae227

Moreover, comments such as “Considering the challenges associated with directly targeting eEF1A1” in line 321 are rather confusing as the authors propose to target this factor indirectly by blocking GBP7. Thus, to correctly interpret their data, they should use the paralelism of the observations they found here (broad spectrum activity by indirect blocking of eEF1A via GBP7 inhibition) with those reported earlier by directly targeting eEF1A.

Minor comments:

-Please review English style in lines 26-28

-Line 60, Not sure if actin binding to eEF1A enables its function as a cofactor in viral replication, the reference 22 does not work with viruses.

-Please include a biosafety statement and procedure approval.

**********

**Part II – Major Issues: Key Experiments Required for Acceptance**

Please use this section to detail the key new experiments or modifications of existing experiments that should be absolutely required to validate study conclusions.

Generally, there should be no more than 3 such required experiments or major modifications for a "Major Revision" recommendation. If more than 3 experiments are necessary to validate the study conclusions, then you are encouraged to recommend "Reject".

Reviewer #1: 1. The authors used a rabbit reticulocyte lysate in vitro translation system, which contains endogenous eEF1A and GBP family proteins. The current experiments cannot confirm that the enhanced translation is specifically mediated by the GBP7-eEF1A1 axis. It would be convinced that repeating the assay in an eEF1A1-depleted translation system to demonstrate that GBP7's pro-translation effect is strictly dependent on eEF1A1.

2. The authors claimed that SAH is a "GBP7-targeting inhibitor", but provided no direct biophysical evidence of a direct interaction between SAH and GBP7. Molecular docking and functional assays alone are insufficient to validate target engagement in a top-tier journal. The authors could perform direct binding assays to confirm that SAH directly binds to GBP7, and validate that the binding occurs at the predicted R48/K51 active site via mutagenesis.

Reviewer #2: 1. The study only shows that GBP7 promotes viral protein translation (such as gD) but does not exclude global effects on host translation in Fig.5. The author needs to provide evidence that GBP7 preferentially enhances viral translation with minimal impact on host translation, supporting target safety and mechanistic specificity. The author can perform Puromycin Incorporation Assay to measure overall protein synthesis rate.

2. Figure 6 serves as the core panel for drug screening and target validation in this study. It was only shown a in silico docking prediction in Fig. 6A. No experimental data (MST/SPR/ITC) are provided to confirm direct physical binding of SAH to GBP7, which is a critical gap in target validation. Direct binding assays must be added and included in the figure. Besides, functional validation using R48A/K51A mutants need to show that SAH loses inhibitory activity against GTPase dead variants, confirming on target activity.

3. The study only shows that HSV‑1 induces GBP7 expression but does not explore whether IFN‑α/β/γ, NF‑κB, or JAK‑STAT pathways mediate this induction, leaving the regulatory mechanism unclear.

Reviewer #3: Experiments to show the cytotoxic/cytostatic effects related to blocking GBP7 should be provided, along clear IC50 and CC50 curves of SAH. Moreover, antiviral activity should be shown using viral protein measurements or CPE rather than PCRs.

**********

**Part III – Minor Issues: Editorial and Data Presentation Modifications**

Please use this section for editorial suggestions as well as relatively minor modifications of existing data that would enhance clarity.

Reviewer #1: 1. The observation that GBP7 knockout or SAH treatment also inhibits VSV, IAV, DENV, and ZIKV is intriguing, but the authors do not demonstrate that the GBP7-eEF1A1 axis mediates these effects. Therefore, the claim of a "broad-spectrum" mechanism should be tempered. Please revise the text to present this as an initial or preliminary observation that requires future validation.

2. The manuscript states survival analysis was performed via the Kaplan–Meier method, but does not report the statistical test used for between-group survival comparisons (the gold standard is the log-rank test). This detail can be added to the main text and figure legend, along with details of normality and variance homogeneity testing for all statistical analyses.

3. Figure 2 shows that the inhibition of virus replication is not significant at the early time point, but is more obvious at the later time point, which may be the result of enhanced antiviral signal transduction.

4. Lines 37-38: The authors incorrectly group cidofovir (a nucleotide analog active against TK-deficient HSV) and pritelivir (a helicase-primase inhibitor) with TK-dependent nucleoside analogues, and include an unapproved drug "anisuvir" with no clinical indication for HSV. These errors should be corrected to ensure accurate background context.

5. Line 67-68: The manuscript includes an internal revision comment ("This revision more clearly communicates the discovery and the functional role of eEF1A1") in the main body of the Introduction. This non-scientific text can be deleted immediately.

6. Line 160: The manuscript uses the incorrect term "GTPase effect domain" (standard nomenclature is "GTPase effector domain, GED"), and repeatedly misclassifies eEF1A1’s function as "initiating translation" (eEF1A1 mediates translation elongation, not initiation).

7. Line 356: The reported puromycin selection concentration of "5 mg/ml" is ~1,000-fold higher than the standard working concentration (1–10 μg/ml) and will cause universal cell death; this is almost certainly a critical typo that must be verified and corrected.

Reviewer #2: 1. In Figure 1, GBP7 induction by HSV‑1 is only validated in A549 cells, without confirmation in HSV‑1‑permissive cells such as Vero, Hela, or primary keratinocytes. The author only showed qualitative IF images without quantification of fluorescence intensity, so the difference in GBP7 expression cannot be objectively evaluated in Fig. 1C and Fig.1G.

2. In Figure 2, the author used single viral MOI lacking dose‑dependence validation (MOI=0.1). No low/medium/high viral gradient is included, so the dose‑dependent proviral effect of GBP7 cannot be confirmed, reducing the robustness of the conclusion. The quality of the fluorescence images in Figure 2K is very poor, the authors should provide clearer images. Also, Panel 2K only shows qualitative images without quantification of fluorescence intensity or GFP‑positive cell percentage, providing only descriptive rather than statistical evidence.

3. Current data identified the binding and interaction between GBP7 and eEF1A1 rely on IP-MS, Co-IP, co-localization, and docking in Fig. 4. In vitro pull‑down assay using purified proteins should be used to confirm direct binding.

4. In Figure 5, the R48A and K51A mutants of GBP7 were only tested for GTPase activity and proviral function. Their binding ability to eEF1A1 was not examined, making it impossible to rule out indirect effects from altered interaction.

5. The author used only three concentrations without reporting EC50, CC50, or selectivity index (SI) in Fig. 6B–D, making it impossible to evaluate the antiviral potency and safety of SAH objectively. Full dose–response curves and calculated parameters are required.

6. In Figure7, the author demonstrated SAH effectively alleviated symptoms and improved survival rate of mice in HSV-1 infection. However, the in vivo pharmacokinetic (PK) data for SAH was loss, these plasma concentration, half‑life, and tissue distribution were missing, hindering interpretation of dose–response relationship. Meanwhile, H&E images in Fig.7H were only qualitative without lesion or inflammation scoring.

7. In Figure 8, the validation of broad‑spectrum antiviral activity is inadequate. The authors only detected GBP7 and viral gene expression at the mRNA level upon infection with diverse viruses but lacked confirmation of GBP7 protein induction and direct measurement of progeny virus titers. No dose–response curves or EC50 values were determined for SAH’s broad‑spectrum activity, making it impossible to objectively evaluate its antiviral potency. Ribavirin, used as a positive control, was only applied at a single dose without parallel comparative analysis. Furthermore, all experiments were performed in cancer cell lines without validation in primary cells or in vivo models. In addition, the common mechanism by which GBP7 regulates both DNA and RNA viruses was not thoroughly explored.

8. Provide quantification graphs for all Western blots, some panels currently show only bands without statistics.

9. The author needs to conduct an in-depth discussion on why interferon-inducing genes have the evolutionary significance for viruses, addressing whether it represents a viral hijacking mechanism or a form of host immune homeostasis regulation. In addition, the authors should further compare the antiviral mechanisms of other GBP family members (GBP1/2/5), particularly in the herpesvirus infection, and explain the structural basis underlying the distinct functional properties of GBP7.

Reviewer #3: (No Response)

**********

PLOS authors have the option to publish the peer review history of their article (what does this mean?). If published, this will include your full peer review and any attached files.

If you choose “no”, your identity will remain anonymous but your review may still be made public.

**Do you want your identity to be public for this peer review?**  For information about this choice, including consent withdrawal, please see our Privacy Policy.

Reviewer #1: No

Reviewer #2: No

Reviewer #3: No

[NOTE: If reviewer comments were submitted as an attachment file, they will be attached to this email and accessible via the submission site. Please log into your account, locate the manuscript record, and check for the action link "View Attachments". If this link does not appear, there are no attachment files.]

**Figure resubmission:**

While revising your submission, we strongly recommend that you use PLOS’s NAAS tool (https://ngplosjournals.pagemajik.ai/artanalysis) to test your figure files. NAAS can convert your figure files to the TIFF file type and meet basic requirements (such as print size, resolution), or provide you with a report on issues that do not meet our requirements and that NAAS cannot fix.

After uploading your figures to PLOS’s NAAS tool - https://ngplosjournals.pagemajik.ai/artanalysis, NAAS will process the files provided and display the results in the "Uploaded Files" section of the page as the processing is complete. If the uploaded figures meet our requirements (or NAAS is able to fix the files to meet our requirements), the figure will be marked as "fixed" above. If NAAS is unable to fix the files, a red "failed" label will appear above. When NAAS has confirmed that the figure files meet our requirements, please download the file via the download option, and include these NAAS processed figure files when submitting your revised manuscript.

**Reproducibility:**

To enhance the reproducibility of your results, we recommend that authors of applicable studies deposit laboratory protocols in protocols.io, where a protocol can be assigned its own identifier (DOI) such that it can be cited independently in the future. Additionally, PLOS ONE offers an option to publish peer-reviewed clinical study protocols. Read more information on sharing protocols at https://plos.org/protocols?utm_medium=editorial-email&utm_source=authorletters&utm_campaign=protocols

# Author comment: Author response to Decision Letter 1

**DOI:** 10.1371/journal.ppat.1014652.r002

13 Aug 2026

Attachment Submitted filename: 0718_Response to Reviewer.docx

# Peer review: Decision Letter 1

**DOI:** 10.1371/journal.ppat.1014652.r003

**Authors:** Alison McBride, Kai Zheng

22 Sep 2026

Dear Dr. Liu,

We are pleased to inform that your manuscript, "GTPase GBP7 promotes HSV-1 infection by interacting with and activating eEF1A1 to

accelerate the protein translation process", has been editorially accepted for publication at PLOS Pathogens.

Before your manuscript can be formally accepted and sent to production, you will need to complete our formatting changes, which you will receive by email within a week. Please note that your manuscript will not be scheduled for publication until you have made the required changes.

IMPORTANT NOTES

(1) Copyediting and Proofreading: The corresponding author will receive a typeset proof for review, to ensure errors have not been introduced during production. Please review the PDF proof of your manuscript carefully, as this is the last chance to correct any errors. Please note that major changes, or those which affect the scientific understanding of the work, will likely cause delays to the publication date of your manuscript.

(2) Appropriate Figure Files: Please remove all name and figure # text from your figure files. Please also take this time to check that your figures are of high resolution, which will improve the readbility of your figures and help expedite your manuscript's publication. Please note that figures must have been originally created at 300dpi or higher. Do not manually increase the resolution of your files. For instructions on how to properly obtain high quality images, please review our Figure Guidelines, with examples at: http://journals.plos.org/plospathogens/s/figures.

We encourage you to use PACE (http://pace.apexcovantage.com/), a figure-checking tool which helps authors ensure all figures meet PLOS requirements so that figures can publish in the highest quality. PACE is a digital diagnostic and conversion tool for figure files. It will provide information about any failed check(s) and, if able, will automatically convert the figure into an acceptable file that passes quality checks.

To use PACE, you must first register as a user. Then, login and navigate to the UPLOAD tab, where you will find detailed instructions on how to use the tool.

(3) Striking Image: Please upload a striking still image to accompany your article if one is available (you can include a new image or an existing one from within your manuscript). Should your paper be accepted, this image will be considered for our monthly issue image and may also appear on our website to feature your article. Please upload this as a separate file, selecting "striking image" as the file type upon upload. Please also include a separate "Other" file with a caption, including credits and any potential copyright information. Please do not include the caption in the main article file. If your image is from someone other than yourself, please ensure that the artist has read and agreed to the terms and conditions of the Creative Commons Attribution License at http://journals.plos.org/plospathogens/s/content-license. Please note that PLOS cannot publish copyrighted images.

(4) Press Release or Related Media: If you or your institution will be preparing press materials for this manuscript, please inform our press team in advance at plospathogens@plos.org as soon as possible. We ask that you contact us within one week to plan ahead of our fast Production schedule. If you need to know your paper's publication date for related media purposes, you must coordinate with our press team, and your manuscript will remain under a strict press embargo until the publication date and time.

(5) Update your Profile Information: Now that your manuscript has been provisionally accepted, please log into Editorial Manager and update your profile, if needed. Go to http://www.editorialmanager.com/ppathogens, log in, and click on the "Update My Information" link at the top of the page. Please update your user information to ensure an efficient production and billing process.

(6) LaTeX users only: Our staff will ask you to upload a TEX file in addition to the PDF before the paper can be sent to typesetting, so please carefully review our Latex Guidelines http://journals.plos.org/plospathogens/s/latex in the meantime.

Thank you again for supporting Open Access publishing; we are looking forward to publishing your work in PLOS Pathogens.

Best regards,

Kai Zheng

Academic Editor

PLOS Pathogens

Alison McBride

Section Editor

PLOS Pathogens

Dominique Soldati-Favre

Editor-in-Chief

PLOS Pathogens

***********************************************************

Reviewer's Responses to Questions

**Part I - Summary**

Please use this section to discuss strengths/weaknesses of study, novelty/significance, general execution and scholarship.<br/><br/>

Reviewer #1: (No Response)

Reviewer #2: The authors have addressed all my comments.

Reviewer #3: I thank the authors for clarifying most of my comments. However, they did not describe the limitations related to the high dose needed to reach efficacy with SAH. I think the paper would benefit if they provide the arguments included in the rebuttal letter in the discussion section.

The authors also state that “plitidepsin can selectively inhibit SARS-CoV-2 virus translation and preserve cell viability at low concentrations[20]” but they cite a report that shows that plitidepsin has a broad-spectrum activity against different viruses, including HSV.

--------------------

**Part II – Major Issues: Key Experiments Required for Acceptance**

Please use this section to detail the key new experiments or modifications of existing experiments that should be absolutely required to validate study conclusions.<br/><br/>Generally, there should be no more than 3 such required experiments or major modifications for a "Major Revision" recommendation. If more than 3 experiments are necessary to validate the study conclusions, then you are encouraged to recommend "Reject".<br/><br/>

Reviewer #1: (No Response)

Reviewer #2: The authors answered all my major questions.

Reviewer #3: (No Response)

--------------------

**Part III – Minor Issues: Editorial and Data Presentation Modifications**

Please use this section for editorial suggestions as well as relatively minor modifications of existing data that would enhance clarity.<br/><br/>

Reviewer #1: (No Response)

Reviewer #2: The authors have addressed all minor issues.

Reviewer #3: (No Response)

--------------------

PLOS authors have the option to publish the peer review history of their article (what does this mean?). If published, this will include your full peer review and any attached files.

If you choose “no”, your identity will remain anonymous but your review may still be made public.

**Do you want your identity to be public for this peer review?**  For information about this choice, including consent withdrawal, please see our Privacy Policy.

Reviewer #1: No

Reviewer #2: No

Reviewer #3: No

# Editor report: Acceptance letter

**DOI:** 10.1371/journal.ppat.1014652.r004

**Authors:** Alison McBride, Kai Zheng

Dear Dr. Liu,

We are delighted to inform you that your manuscript, "GTPase GBP7 promotes HSV-1 infection by interacting with and activating eEF1A1 to

accelerate the protein translation process," has been formally accepted for publication in PLOS Pathogens.

We have now passed your article onto the PLOS Production Department who will complete the rest of the pre-publication process. All authors will receive a confirmation email upon publication.

Thank you again for supporting open-access publishing; we are looking forward to publishing your work in PLOS Pathogens.

Best regards,

Dominique Soldati-Favre

Editor-in-Chief

PLOS Pathogens
